# *Wolbachia* outcompetes mosquito-borne viruses by reprogramming host ATPSβ1-driven ATP metabolism toward symbiotic proliferation

**DOI:** 10.1371/journal.ppat.1014692

**Published:** 2026-10-21

**Authors:** Hao Yuan, Yalan Zhang, Yaqi Zhao, Chaojun Wang, Yuanyuan Zhou, Qian Ni, Xiaodan Li, Bo Zhang, Yong Wang, Liang Cai, Hong Liu, Jinglin Wang, Juntao Gong, Meichun Zhang, Zhiyong Xi, Xiaoling Pan

**Affiliations:** 1 Department of Medical Laboratory Science, School of Medical Technology and Translational Medicine, Hunan Normal University Health Science Center, Changsha, Hunan, P. R. China; 2 Key Laboratory of Special Pathogens and Biosafety, Wuhan Institute of Virology, Center for Biosafety Mega-Science, Chinese Academy of Sciences, Wuhan, P. R. China; 3 Department of Forensic Science, School of Basic Medical Science, Central South University, Changsha, Hunan, P. R. China; 4 Hunan Provincial Center for Disease Control and Prevention, Changsha, Hunan, P. R. China; 5 School of Life Sciences and Medicine, Shandong University of Technology, Zibo, P. R. China; 6 Kunming Medical University, Kunming, Yunnan, P. R. China; 7 Guangzhou Wolbaki Biotech Co., Ltd., Guangzhou, Guangdong, P. R. China; 8 Jiangsu Institute of Parasitic Diseases, Wuxi, Jiangsu, P. R. China; 9 Department of Microbiology, Genetics, & Immunology, Michigan State University, East Lansing, Michigan, United States of America; Pennsylvania State University - Main Campus: The Pennsylvania State University - University Park Campus, UNITED STATES OF AMERICA

## Abstract

*Wolbachia* endosymbionts confer broad-spectrum resistance to mosquito-borne viruses. While the antiviral metabolites in *Wolbachia*-*Aedes* have been identified during viral replication, the core metabolic gene underlying this defense across the entire viral infection cycle remains undefined. Here, we demonstrated for the first time that *Wolbachia* strongly suppressed the emerging alphavirus Venezuelan equine encephalitis virus, extending its known antiviral spectrum. Transcriptomic and functional analyses revealed ATP synthase beta subunit 1 (*ATPSβ1*) as a key metabolic hub hijacked by both MBV and the symbiont. Without *Wolbachia* symbiosis, dengue virus and VEEV induced ATPSβ1 to promote viral binding via the viral envelope protein and increased intracellular ATP production for viral replication. Conversely, *Wolbachia* overrode *ATPSβ1*-mediated viral binding and redirected *ATPSβ1*-driven ATP synthesis to fuel its proliferation, strengthening antiviral activity. Pharmacologically, adenosine increased *Wolbachia w*AlbB density and potently suppressed viral infection in *Ae. aegypti* and *Ae. albopictus* cells. Oligomycin reduced intracellular ATP and inhibited viral infection in *Wolbachia*-free *Ae. aegypti* cells. Collectively, *Wolbachia* repurposes dual-functional *ATPSβ1*, which governs MBV binding and replication, into a potent antiviral effector through a metabolic tug-of-war over host ATP during symbiont-virus competition. Moreover, we highlight interventions targeting the *ATPSβ1*-driven metabolism as promising strategies to curb MBV transmission.

## Author summary

Mosquito-borne viruses (MBVs), such as dengue virus (DENV) and Venezuelan equine encephalitis virus (VEEV), pose serious threats to global public health. The endosymbiotic bacterium *Wolbachia* has been developed as a powerful tool for blocking virus transmission. However, the core metabolic mechanisms underlying *Wolbachia-*induced broad-spectrum antiviral resistance, especially across the complete viral infection cycle, remain unclear. In this study, we uncover ATP synthase beta subunit 1 (ATPSβ1) as a previously unrecognized metabolic hub that governs both viral infection and *Wolbachia* symbiosis. Importantly, we demonstrate that pharmacological modulation of ATPSβ1-driven metabolism potently suppresses MBV infection. Furthermore, we establish ATPSβ1 as a novel molecular target for developing small-molecule interventions that could recapitulate *Wolbachia*’s antiviral effect, even in the absence of the symbiont. Collectively, this work shifts the paradigm from viewing *Wolbachia* only as a blocker of viral transmission to understanding it as an active metabolic competitor. It also opens a new avenue for developing broad-spectrum antiviral strategies targeting host ATP metabolism, offering a transformative approach to mitigating the global public health burden of mosquito-borne diseases.

## Introduction

Recently, the geographical distribution of *Aedes aegypti* and *Aedes albopictus*, the primary vectors of several medically important mosquito-borne viruses (MBVs), has been progressively expanding into temperate regions, driven by climate change, globalization, and urbanization [1,2]. Consequently, MBVs, which are traditionally predominantly prevalent in tropical areas, have also spread to temperate zones [3,4]. Currently, more than half of the global population is at risk of infection with these viruses [5,6]. The epidemic spread of these viruses causes hundreds of millions of infections and millions of deaths annually, with estimated economic losses of approximately $12 billion USD [7–9], and poses a serious threat to human health associated with symptoms such as severe neurological sequelae [10,11]. Given the current absence of specific antiviral drugs for treating mosquito-borne diseases [12,13], novel strategies to effectively control MBV transmission by *Ae. aegypti* and *Ae. albopictus* are urgently needed.

The gram-negative intracellular endosymbiont *Wolbachia* has been utilized to develop innovative biological control tools that exploit its ability to strongly inhibit or even block host mosquitoes from transmitting various MBVs [14,15]. These cutting-edge tools have achieved remarkable success in field applications to control Dengue virus (DENV) transmission in *Aedes* mosquitoes, marking a major breakthrough in mosquito-borne disease prevention [16–19]. Particularly, the *Wolbachia* strain *w*AlbB confers broad-spectrum antiviral resistance on both *Ae. albopictus* and *Ae. aegypti* hosts, targeting MBVs including alphaviruses, including Sindbis virus, Balaton Forest virus, Semliki Forest virus, Chikungunya virus, and Mayaro virus [20–23], as well as the orthoflavivirus DENV and Zika virus (ZIKV) [24,25]. This prominent feature makes *w*AlbB the optimal *Wolbachia* strain for generating antiviral mosquito lines. Nevertheless, it remains completely uncharacterized whether this endosymbiont interferes with the transmission of Venezuelan equine encephalitis virus (VEEV), a highly pathogenic MBV zoonotic posing a severe public health threat. Recently, VEEV has caused large-scale outbreaks across the Americas, with up to 75,000 infections in a single outbreak [26,27]. Therefore, an in-depth investigation into the antiviral spectrum of *Wolbachia* is essential for comprehensively evaluating the feasibility of extending this pioneering biological control technology to other major vectors, particularly *Culex* mosquitoes, which are important global vectors of VEEV, thereby reinforcing global defenses against emerging high-risk MBVs.

Although substantial progress has been made in identifying antiviral metabolites in *Wolbachia*–*Aedes* symbiotic systems during viral replication [28–32], these metabolites cannot fully account for the broad-spectrum anti-MBV activity induced by *Wolbachia* throughout the viral life cycle. Specifically, the *Wolbachia*-manipulated metabolites characterized to date, including amino acids, lipids, carbohydrates, and cholesterol, exert phase-specific antiviral effects exclusively during the replication phase of DENV and ZIKV [28–34]. However, *Wolbachia* has been demonstrated to trigger potent antiviral inhibition as early as the binding phase of DENV and Zika virus infection in *Aedes* [35,36]. Accordingly, the core hub metabolic gene that underlies the robust inhibition of the entire MBV infection cycle mediated by *Wolbachia* remains poorly defined. Strikingly, nearly all phases of the viral infection cycle are strictly energy-dependent and rely heavily on adenosine triphosphate (ATP) [37]. This universal energetic dependency highlights ATP as a potential linchpin of antiviral defense, and a recent report further links *Wolbachia* to host ATP regulation in the small brown planthopper (*Laodelphax striatellus*), thereby facilitating host ovarian development [38]. Moreover, as the primary organelles responsible for ATP biosynthesis in host cells, mitochondria also exhibit significant transcriptional reprogramming in response to *Wolbachia* colonization [39]. Taken together, these findings indicate that *Wolbachia* most likely modulates ATP production and ATP-dependent metabolite synthesis via a pivotal energy-centered regulatory axis across the entire MBV infection cycle in *Aedes* hosts, thereby exerting its broad-spectrum antiviral effect. Elucidating this key mechanism will not only advance the application of *Wolbachia*-based mosquito control strategies but also provide critical targets for developing broad-spectrum anti-MBV drugs.

To address this hypothesis, in the present study, *Ae. aegypti* and *Ae. albopictus* carrying the *Wolbachia* strain *w*AlbB were used as cell models. DENV (Flaviviridae, *Orthoflavivirus* genus) and VEEV (Togaviridae, *Alphavirus* genus) pseudoviruses were selected as the representative MBVs. By comparing transcriptomic changes in *Wolbachia*-infected and *Wolbachia*-uninfected *Ae. aegypti* cells post MBV infection, we aimed to identify broad-spectrum energy metabolism molecules with anti-MBV activity in *Aedes* and to further investigate their antiviral effects and mechanisms during viral binding and early replication events. Building upon these identified mechanisms, we further assessed the ability of antiviral metabolite treatment to inhibit MBV infection in *Ae. aegypti* and *Ae. albopictus* cells. Collectively, our research provides new insights into the metabolic mechanisms underlying *Wolbachia*-mediated ATP regulation of *Aedes*-MBV interactions and offers new targets for the development of sustainable MBV control strategies.

## Results

### Broad-spectrum antiviral inhibition by *Wolbachia* extends to Venezuelan equine encephalitis virus in *Aedes aegypti* cells

To assess the impact of *Wolbachia* on mosquito-borne virus MBV infection in *Ae. aegypti*, DENV (an orthoflavivirus) and VEEV (an alphavirus) were selected as representative MBVs. First, the cytoplasmic localization of *Wolbachia w*AlbB and its increasing density from day 1 (D1) to day 3 (D3) in *Wolbachia-*infected (W+) *Ae. aegypti* cells were validated using fluorescence in situ hybridization (FISH; Figs 1A and 1B) and quantitative PCR (qPCR; Fig 1C). In contrast, no *Wolbachia* was detected in *Wolbachia*-free (W-) *Ae. aegypti* cells, which served as the negative control (Figs 1A-1C). Using these validated W- and W+ cells, *Wolbachia*-mediated inhibition of DENV was evaluated with DENV pseudoviruses generated by cotransfecting BHK-21 cells with a DENV replicon and a plasmid expressing the DENV structural protein (DENV-SP) (Figs 1D and S1). Following infection at a multiplicity of infection (MOI) of 0.1, DENV infection was consistently and significantly suppressed in W+ cells compared with W- cells across all time points, with peak inhibition (7.85-fold) observed at 24 h post-infection (hpi; Figs 1E and S1), further supporting the known anti-DENV ability of *Wolbachia* in *Aedes* cells and mosquitoes [26,41]. To address the knowledge gap regarding whether *Wolbachia* also inhibits VEEV, another medically important emerging MBV, a comparable pseudovirus infection assay was performed for VEEV (MOI = 0.1) using pseudoviruses produced as described above, with a VEEV replicon and a VEEV structural protein plasmid (VEEV-SP) (Figs 1F and S1). Notably, VEEV infection was significantly reduced in W+ cells at all time points tested, with the strongest suppression (4.94-fold) occurring at 12 hpi (Fig 1G). These results demonstrate for the first time that *Wolbachia* exerts antiviral activity against VEEV in *Ae. aegypti* cells, indicating a broader spectrum of inhibitory effects on MBVs than previously reported.

**Fig 1:**
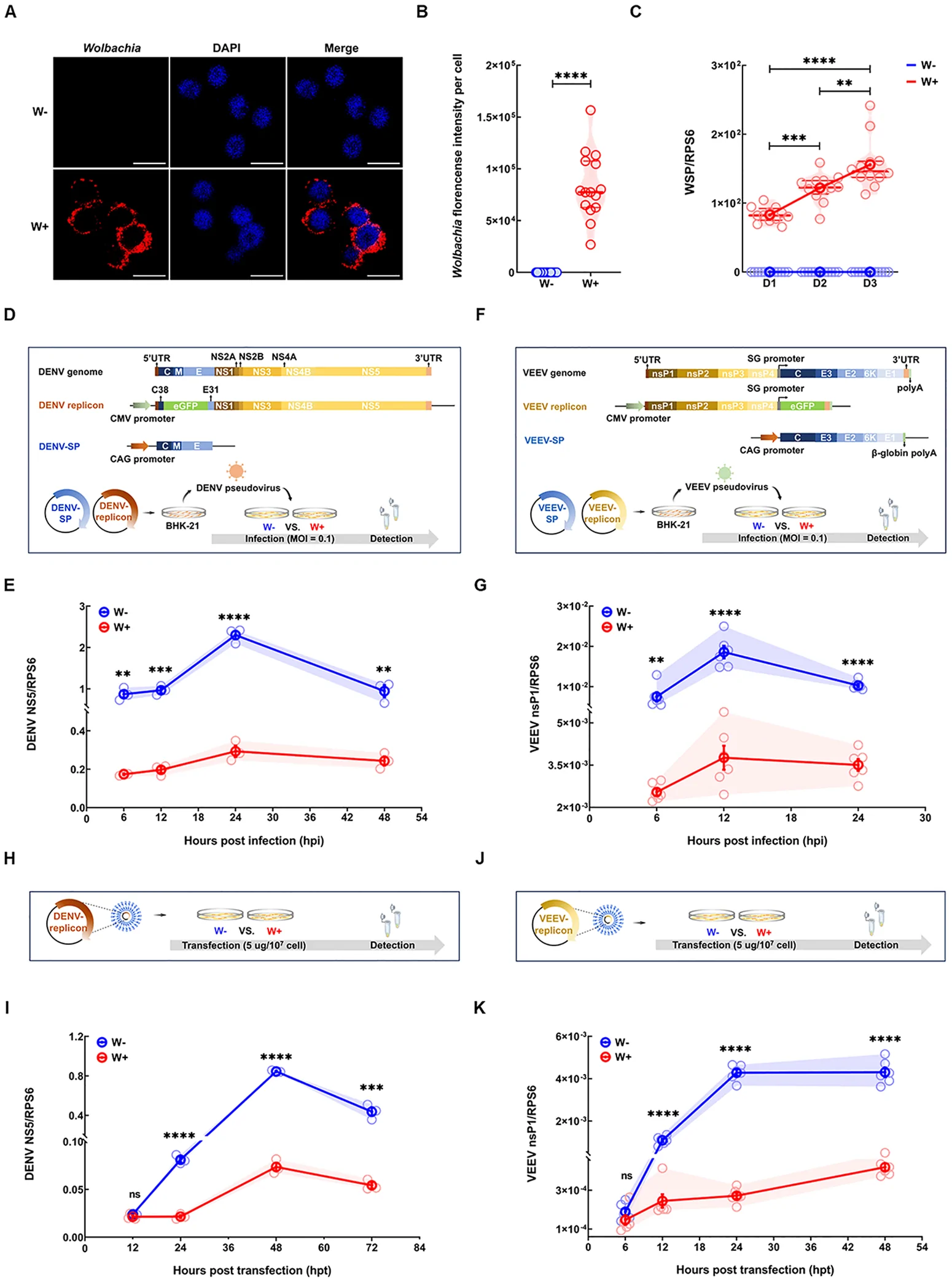
*Wolbachia* inhibits Venezuelan equine encephalomyelitis virus (VEEV) and dengue virus (DENV) infection and replication in *Aedes aegypti* cells. **(A)** Representative fluorescence images of *Wolbachia* in *Wolbachia*-infected (W+) and *Wolbachia*-free **(W-)** *Ae. aegypti* cells. The scale bar is 10 µm. **(B)** Quantification of *Wolbachia* fluorescence intensity in *Ae. aegypti* cells (two-sided t test, n = 15 per group, *P* = 8.864400 × 10^-8^). **(C)** *Wolbachia* density dynamics in W*+* cells on day 1 (D1), day 2 (D2), and day 3 (D3) post-cell seeding (one-way ANOVA, n = 12 per group; D1 vs. D2: *P* = 0.000518; D2 vs. D3: *P* = 0.002077; D1 vs. D3: *P* = 3.060600 × 10^-8^). **(D)** Schematic diagram of the DENV genome, pseudovirus production, and infection. The upper panel shows the DENV replicon and the DENV structural protein plasmid (DENV-SP) utilized for pseudovirus production. The lower panel illustrates the workflow for DENV pseudovirus production and subsequent infection of W- and W+ cells. **(E)** Dynamics of DENV infection levels in W- and W+ cells at 6 h post-infection (hpi) (repeated-measures ANOVA, n = 3 per group, *P* = 0.001336), 12 hpi (repeated-measures ANOVA, n = 3 per group, *P* = 0.000331), 24 hpi (repeated-measures ANOVA, n = 3 per group; fold change = -7.85; *P* = 0.000049), and 48 hpi (repeated-measures ANOVA, n = 3 per group, *P* = 0.008715). **(F)** Schematic of diagrams of VEEV pseudovirus production and infection. The upper panel depicts the VEEV replicon and VEEV structural protein plasmid (VEEV-SP) used for VEEV pseudovirus generation. The bottom panel shows the workflow of VEEV pseudovirus production and the subsequent viral infection of W- and W+ cells. **(G)** Comparison of VEEV infection levels between W- and W+ cells at 6 hpi (repeated-measures ANOVA, n = 6 per group, *P* = 0.001364), 12 hpi (repeated-measures ANOVA, n = 6 per group; fold change = -4.94; *P* = 0.000003), and 24 hpi (repeated-measures ANOVA, n = 6 per group, *P* = 5.698800 × 10^-8^). **(H)** Schematic of the DENV replication assay in W- and W+ cells. **(I)** Dynamics of DENV replication levels between W- and W+ cells at 12 h post-transfection (hpt) (repeated-measures ANOVA, n = 3 per group, *P* = 0.243429), 24 hpt (repeated-measures ANOVA, n = 3 per group, *P* = 0.000041), 48 hpt (repeated-measures ANOVA, n = 3 per group; fold change = -11.50; *P* = 5.219400 × 10^-8^), and 72 hpt (repeated-measures ANOVA, n = 3 per group, *P* = 0.000965). **(J)** Schematic of the VEEV replication assay in W- and W+ cells. **(K)** Comparison of VEEV replication levels between W- and W+ cells at 6 hpt (repeated-measures ANOVA, W-: n = 6, W + : n = 6, *P* = 0.186351), 12 hpt (repeated-measures ANOVA, W-: n = 6, W + : n = 6, *P* = 0.000002), 24 hpt (repeated-measures ANOVA, n = 6 per group; fold change = -15.77; *P* = 1.225600 × 10^-10^), and 48 hpt (repeated-measures ANOVA, n = 6 per group, *P* = 1.035600 × 10^-8^). The blue circles in the schematic diagram (**H** & **J**) indicate the particles with viral replicons encapsulated by the lipid bilayer of the transfection reagent. The horizontal lines within the scatter plots (**B** & **C**) from bottom to top indicate the 25^th^ (Q1), 50^th^ (median), and 75^th^ (Q3) percentiles. The error bars indicate the standard error of the mean (SEM). ****P < 0.0001, ***P < 0.001, **P < 0.01, and ns indicates nonsignificance.

Building on reports that *Wolbachia* inhibits DENV binding [35], we extended this investigation to determine its effect on MBV replication independently of viral binding. In the viral replication assay, a DENV replicon plasmid was directly transfected into W- and W+ cells, which bypasses viral binding (Fig 1H). Although DENV genomic RNA (gRNA) was comparable between W- and W+ cells at 12 h post-transfection (hpt), a significant reduction in DENV gRNA was observed in W+ cells, with an 11.50-fold decrease at 48 hpt compared with that in W- cells (Fig 1I). Similarly, even transfection with a VEEV replicon (Fig 1J) caused no significant difference in viral gRNA between W- and W+ cells at 6 hpt, it resulted in a marked 15.77-fold suppression of VEEV gRNA in W+ cells at 24 hpt (Fig 1K). Collectively, these data demonstrate that *Wolbachia* exerts broad-spectrum antiviral activity in *Ae. aegypti* cells that encompasses both the initial infection and the intracellular replication of diverse MBVs.

### *Wolbachia*-induced ATP synthase beta subunit 1 expression restricts MBV infection and replication in *Ae. aegypti* cells

To identify the key factors underlying *Wolbachia*-mediated MBV inhibition, the transcriptional profile alterations triggered by *Wolbachia* were compared between W+ and W- cells infected with VEEV (12 hpi) or DENV (24 hpi) via RNA sequencing. Applying a threshold of a false discovery rate (FDR) < 0.05 and a |log_2_ fold change (FC)| ≥ 1, we identified 2,026 differentially expressed genes (DEGs; 635 upregulated and 1,391 downregulated) in response to DENV pseudovirus infection and 1,860 DEGs (678 upregulated and 1,182 downregulated, S1 Table) in response to VEEV pseudovirus infection. Kyoto Encyclopedia of Genes and Genomes (KEGG) pathway analysis highlighted the top 5 activated pathways during MBV infection, which included oxidative phosphorylation, inositol phosphate metabolism, biosynthesis of amino acids, valine/leucine/isoleucine degradation, and the spliceosome (Fig 2A). Notably, among these pathways, the ATP synthase beta subunit 1 (*ATPSβ1*) gene (VectorBase ID: AAEL002827) exhibited the greatest fold change in the oxidative phosphorylation-related DEGs (*ATPSβ1*, *V-ATPase D1*, *Ndufa9*, and *Ndufs1*) during infection with either VEEV or DENV according to both the RNA sequencing and qPCR validation (Figs 2A-2D). *ATPSβ1* encodes a core component of mitochondrial respiratory complex V (ATP synthase), which is located primarily in the inner mitochondrial membrane [42,43] (Fig 2B). Consistent with the transcriptional upregulation, ATPSβ1 protein levels were also markedly elevated by *Wolbachia* during MBV infection (Figs 2E-2H). Given the concurrent upregulated expression of ATP hydrolysis-related *V-ATPase D1* and ATP synthesis-related *ATPSβ1* genes (Figs 2A-2D), we measured intracellular ATP levels and found that *Wolbachia* significantly increased *ATPSβ1* gene expression to promote ATP production in W+ cells pre- and post-infection with either VEEV or DENV (Figs 2I and 2J). These findings indicate that *Wolbachia* enhances both the transcriptional and translational expression of *ATPSβ1*, thereby increasing intracellular ATP production in *Ae. aegypti* cells during MBV infection.

**Fig 2:**
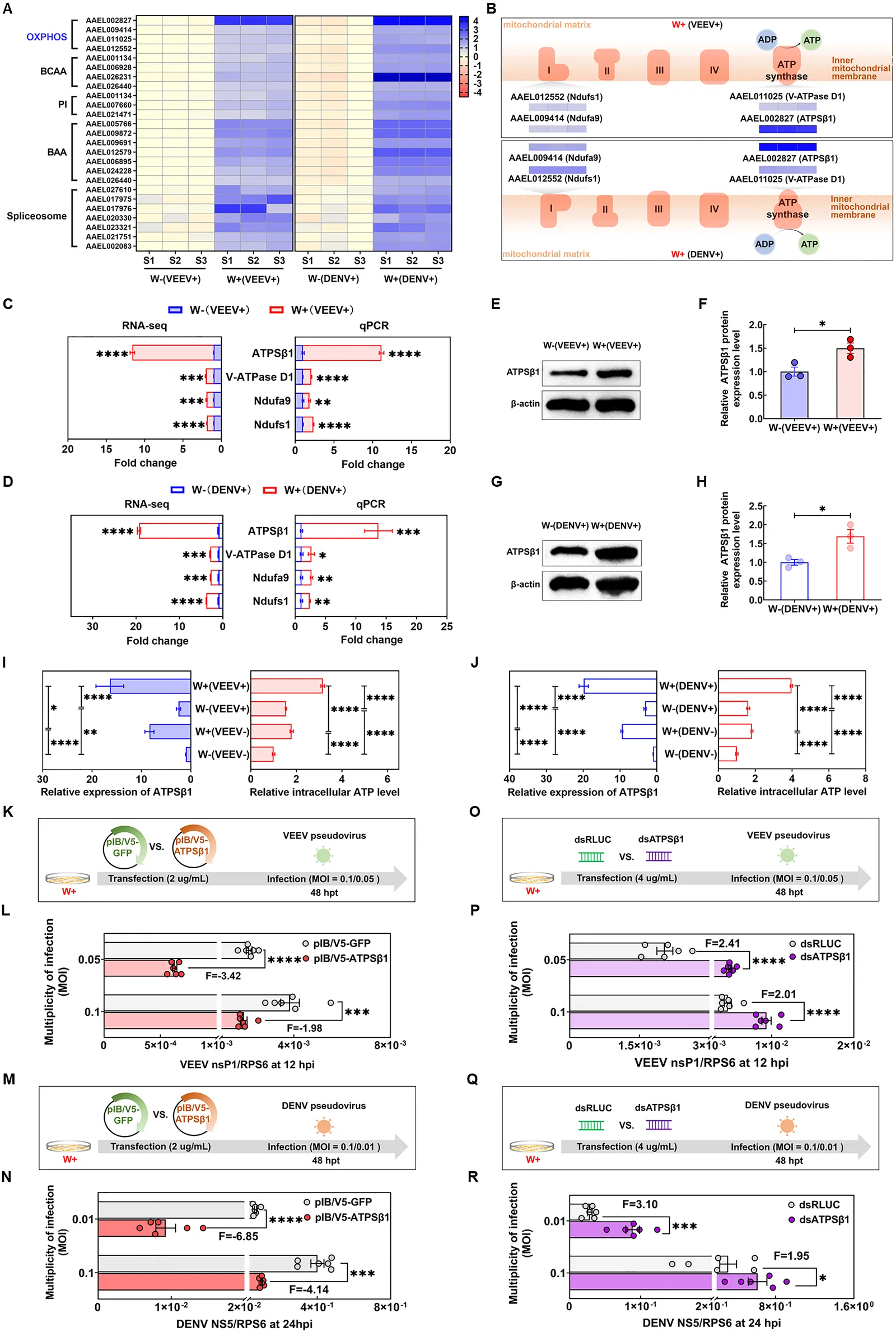
*Wolbachia*-induced ATP synthase beta subunit 1 (ATPSβ1) inhibits MBV infections in *Ae. aegypti* cells. **(A)** Top 5 pathways activated by *Wolbachia* at 12 hpi with VEEV or 24 hpi with DENV. The colors represent log_2_-fold change values that were calculated on the basis of fragments per kilobase of transcript per million mapped reads (FPKM). **(B)** Relative expression and subcellular localization of *Wolbachia*-activated oxidative phosphorylation pathway genes in the respiratory chain complex during VEEV or DENV infection. **(C)** Validation of *Wolbachia*-activated oxidative phosphorylation pathway genes at 12 hpi with VEEV. The left panel shows the RNA sequencing results (two-sided t test, n = 3 per group; *ATPSβ1*: *P* = 0.000004; *V-ATPase D1*: *P* = 0.000200; *Ndufa9*: *P* = 0.000586; *Ndufs1*: *P* = 0.000021), and the right panel shows the results of the quantitative PCR (qPCR) validation (two-sided t test, n = 3 per group; *ATPSβ1*: *P* = 0.000056; *V-ATPase D1*: *P* = 0.000026; *Ndufa9*: *P* = 0.008579; *Ndufs1*: *P* = 0.000081). **(D)** Verification of *Wolbachia*-activated oxidative phosphorylation pathway genes at 24 hpi with DENV. The left panel shows the RNA sequencing results (two-sided t test, n = 3 per group; *ATPSβ1*: *P* = 0.000002; *V-ATPase D1*: *P* = 0.000288; *Ndufa9*: *P* = 0.000187; *Ndufs1*: *P* = 0.000040), and the right panel represents the results of the qPCR validation (two-sided t test, n = 3 per group; *ATPSβ1*: *P* = 0.000496; *V-ATPase D1*: *P* = 0.029713; *Ndufa9*: *P* = 0.007818; *Ndufs1*: *P* = 0.008086). **(E)** Representative western blot images of ATPSβ1 protein in W- and W+ cells at 24 hpi with VEEV. **(F)** Quantification of ATPSβ1 protein levels in VEEV-infected W- and W+ cells at 24 hpi (two-sided t test, n = 3 per group, *P* = 0.027574). **(G)** Representative western blot images of ATPSβ1 protein in W- and W+ cells at 48 hpi with DENV. **(H)** Quantification of ATPSβ1 protein levels in DENV-infected W- and W+ cells at 48 hpi (two-sided t test, n = 3 per group, *P* = 0.024584). **(I)** Relative expression of the *ATPSβ1* gene and intracellular ATP levels in W- and W+ cells at 12 hpi with infectious VEEV pseudovirus (VEEV+) or heat-inactivated VEEV pseudovirus (VEEV-). The left panel shows relative expression of the *ATPSβ1* gene (one-way ANOVA, n = 3 per group; W-(VEEV-) vs. W+(VEEV-): *P* = 0.000007; W-(VEEV+) vs. W+(VEEV+): *P* = 0.000016; W-(VEEV-) vs. W-(VEEV+): *P* = 0.002684; W+(VEEV-) vs. W+(VEEV+): *P* = 0.012731), and the right panel shows intracellular ATP levels (one-way ANOVA, n = 8 per group; W-(VEEV-) vs. W+(VEEV-): *P* = 1.172400 × 10^-9^; W-(VEEV+) vs. W+(VEEV+): *P* = 5.248100 × 10^-17^; W-(VEEV-) vs. W-(VEEV+): *P* = 0.000001; W+(VEEV-) vs. W+(VEEV+): *P* = 3.765500 × 10^-15^). **(J)** Relative expression of the *ATPSβ1* gene and intracellular ATP levels in W- and W+ cells at 24 hpi with infectious DENV pseudovirus (DENV+) or heat-inactivated DENV pseudovirus (DENV-). The left panel shows relative expression of the *ATPSβ1* gene (one-way ANOVA, n = 3 per group; W-(DENV-) vs. W+(DENV-): *P* = 7.280400 × 10^-9^; W-(DENV+) vs. W+(DENV+): *P* = 3.654400 × 10^-8^; W-(DENV-) vs. W-(DENV+): *P* = 0.000001; W+(DENV-) vs. W+(DENV+): *P* = 0.000038), and the right panel shows intracellular ATP levels (one-way ANOVA, n = 8 per group; W-(DENV-) vs. W+(DENV-): *P* = 1.720400 × 10^-9^; W-(DENV+) vs. W+(DENV+): *P* = 1.036700 × 10^-20^; W-(DENV-) vs. W-(DENV+): *P* = 3.791500 × 10^-7^; W+(DENV-) vs. W+(DENV+): *P* = 1.143000 × 10^-19^). **(K)** Schematic of the VEEV infection assay following *ATPSβ1* overexpression in W+ cells. pIB/V5-ATPSβ1: *ATPSβ1* overexpression group; pIB/V5-GFP: control group. **(L)** Changes in the VEEV infection level in response to *ATPSβ1* overexpression in W+ cells at 12 hpi (two-sided t test, n = 6 per group; MOI = 0.1: *P* = 0.000862; MOI = 0.05: *P* = 0.000042). **(M)** Schematic of the DENV infection assay following *ATPSβ1* overexpression in W+ cells. **(N)** Changes in DENV infection levels in W+ *ATPSβ1*-overexpressing cells at 24 hpi (two-sided t test, n = 6 per group; MOI = 0.1: *P* = 0.000338; MOI = 0.01: *P* = 0.000008). **(O)** Schematic of the VEEV infection assay following *ATPSβ1* knockdown in W+ cells. dsATPSβ1: *ATPSβ1* knockdown group; dsRLUC: control group. **(P)** Alteration of VEEV infection levels triggered by *ATPSβ1* knockdown in W+ cells at 12 hpi (two-sided t test, n = 6 per group; MOI = 0.1: *P* = 0.000059; MOI = 0.05: *P* = 0.000001). **(Q)** Schematic of the DENV infection assay following *ATPSβ1* knockdown in W+ cells. **(R)** Changes in DENV infection level upon *ATPSβ1* knockdown in W+ cells at 24 hpi. (two-sided t test, n = 6 per group; MOI = 0.1: *P* = 0.040582; MOI = 0.01: *P* = 0.000137). OXPHOS: oxidative phosphorylation; BCAA: valine/leucine/isoleucine degradation; PI: inositol phosphate metabolism; BAA: biosynthesis of amino acids. VEEV + : infection with VEEV pseudovirus; DENV + : infection with DENV pseudovirus. *ATPSβ1*: ATP synthase beta subunit 1; *V-ATPase D1*: V-type proton ATPase subunit d1; *Ndufa9*: NADH dehydrogenase 1 alpha subcomplex subunit 9; *Ndufs1*: NADH-ubiquinone oxidoreductase 75 kDa subunit. dsATPSβ1: *ATPSβ1* knockdown group, dsRLUC: control group. The error bars indicate the SEMs. F indicates the fold change value. ****P < 0.0001, ***P < 0.001, **P < 0.01, *P < 0.05.

To evaluate the role of *ATPSβ1* in *Wolbachia*-mediated antiviral activity, parallel infections with MBVs at high and low MOIs were performed in W+ cells with manipulation of *ATPSβ1* expression. Specifically, a 2.16-fold increase in *ATPSβ1* expression achieved by transfection with the pIB/V5-ATPSβ1 plasmid had no significant effect on cell viability but consistently inhibited viral infection across all the MOIs tested (Figs 2K-2N, S2A, and S3A). Overexpression of *ATPSβ1* resulted in a 3.42-fold suppression of VEEV infection at an MOI of 0.05 and a 6.85-fold decrease in DENV infection at an MOI of 0.01 (Figs 2L-2N). Conversely, double-stranded RNA (dsRNA)-mediated depletion of *ATPSβ1* in W+ cells had no significant effect on cell viability but enhanced VEEV infection by 2.01-fold (MOI = 0.1) and 2.41-fold (MOI = 0.05) at 12 hpi and increased DENV infection by 1.95-fold (MOI = 0.1) and 3.10-fold (MOI = 0.01) at 24 hpi (Figs 2O-2R, S2B, and S3B). These results demonstrate that *ATPSβ1* is a key *Wolbachia*-induced factor that inhibits MBV infection in *Ae. aegypti* cells.

### An increase in intracellular ATP levels induced by *Wolbachia* via *ATPSβ1* promotes its symbiotic density to inhibit MBV replication

We next investigated how *Wolbachia* exploits *ATPSβ1* to inhibit MBVs. Overexpression of ATPSβ1 at both mRNA and protein levels via the pIB/V5-ATPSβ1 plasmid in W+ cells significantly increased ATP synthase activity and intracellular ATP levels at 48 hpt, which was accompanied by a 2.15-fold increase in *Wolbachia* density at 72 hpt (Figs 3A-3E). FISH analysis further confirmed markedly stronger *Wolbachia*-specific fluorescent signals in *ATPSβ1*-overexpressing cells (Figs 3F and 3G). Conversely, depletion of *ATPSβ1* via dsATPSβ1 in W+ cells reduced ATPSβ1 at mRNA and protein levels, thereby reducing both ATP synthase activity and intracellular ATP levels and subsequently decreasing *Wolbachia* density by 5.31-fold (Figs 3H-3L), as corroborated by weaker *Wolbachia*-specific fluorescent signals in FISH analysis (Figs 3M and 3N). These results indicate that upregulation of *ATPSβ1* expression increases ATP synthase activity and intracellular ATP production, thereby promoting *Wolbachia* proliferation in *Ae. aegypti* cells.

**Fig 3:**
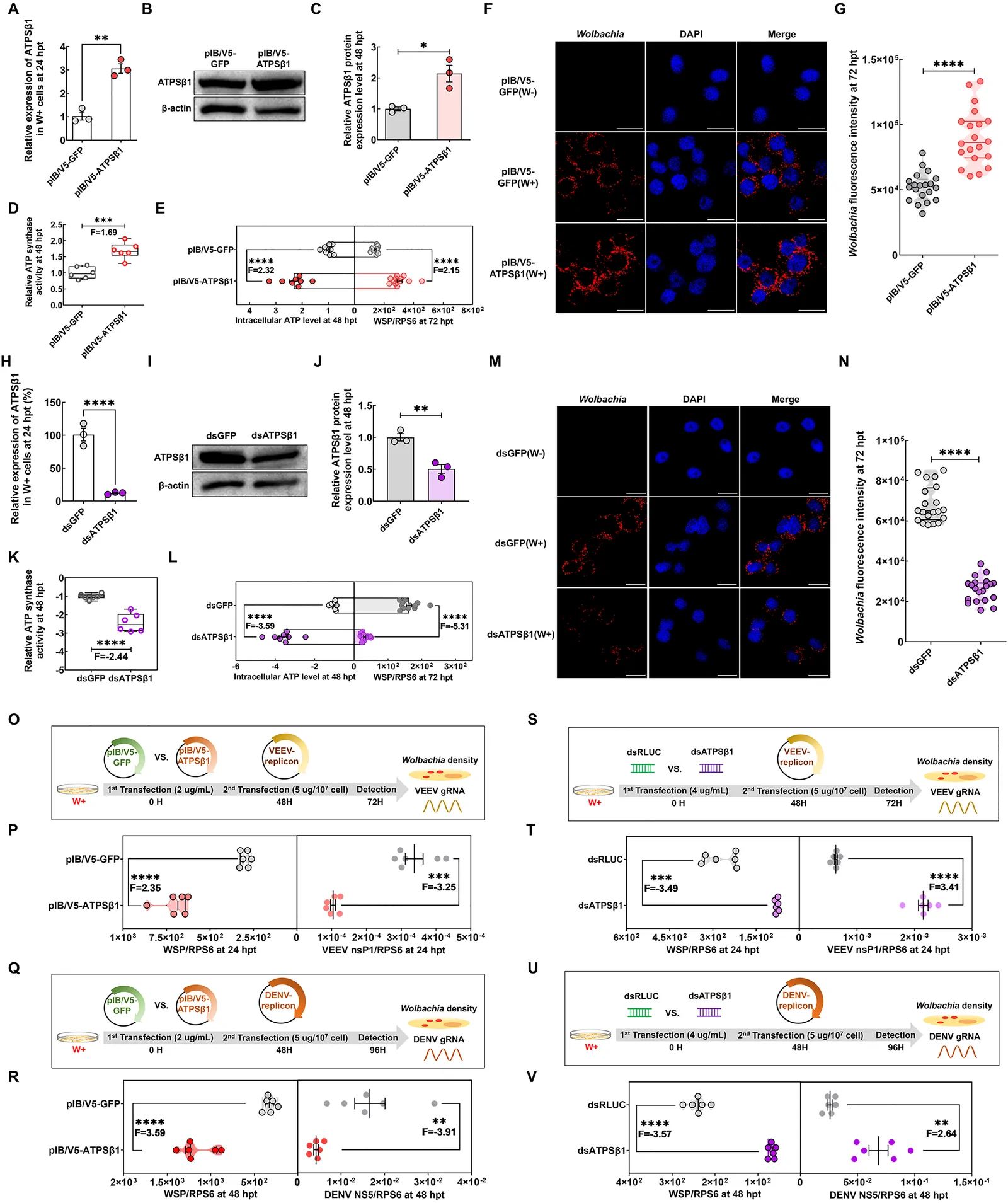
*ATPSβ1* inhibits MBV replication by increasing intracellular ATP levels and *Wolbachia* density in *Ae. aegypti* cells. **(A)** *ATPSβ1* mRNA expression in W+ cells transfected with pIB/V5-ATPSβ1 vs. pIB/V5-GFP at 24 hpt (two-sided t test, n = 3 per group; *P* = 0.002750). **(B)** Representative western blot images of ATPSβ1 protein in W+ cells correspond to *ATPSβ1* overexpression at 48 hpt. **(C)** Quantification of ATPSβ1 protein levels in W+ cells transfected with pIB/V5-ATPSβ1 vs. pIB/V5-GFP at 48 hpt (two-sided t test, n = 3 per group; *P* = 0.014395). **(D)** ATP synthase activity in response to *ATPSβ1* overexpression in W+ cells at 48 hpt (two-sided t test, n = 6 per group, *P* = 0.000317). **(E)** Intracellular ATP levels at 48 hpt (left panel; two-sided t test, n = 8 per group; *P* = 0.000006) and *Wolbachia* density at 72 hpt (right panel; two-sided t test, n = 12 per group; *P* = 6.532500 × 10^-7^) in W+ cells following ATPSβ1 overexpression. **(F)** Representative fluorescence images of *Wolbachia* in cells in response to *ATPSβ1* overexpression at 72 hpt. The scale bar is 10 µm. **(G)** *Wolbachia* fluorescence intensity in W+ cells transfected with pIB/V5-ATPSβ1 vs. pIB/V5-GFP at 72 hpt (two-sided t test; n = 20 per group; *P* = 1.120900 × 10^-7^). **(H)** *ATPSβ1* mRNA levels in W+ cells transfected with dsATPSβ1 vs. dsGFP at 24 hpt (two-sided t test, n = 3 per group, *P* = 0.000088). **(I)** Representative western blot images of ATPSβ1 protein in W+ cells correspond to *ATPSβ1* downregulation at 48 hpt. **(J)** Quantification of ATPSβ1 protein levels in W+ cells transfected with dsATPSβ1 vs. dsGFP at 48 hpt (two-sided t test, n = 3 per group, *P* = 0.005274). **(K)** ATP synthase activity in W+ cells post *ATPSβ1* knockdown at 48 hpt (two-sided t test, n = 6 per group, *P* = 0.000012). **(L)** Intracellular ATP levels at 48 hpt (left panel; two-sided t test, n = 8 per group; *P* = 1.973900 × 10^-10^) and *Wolbachia* density at 72 hpt (right panel; two-sided t test, n = 12 per group; *P* = 1.904700 × 10^-10^) in W+ cells following *ATPSβ1* knockdown. **(M)** Representative fluorescence images of *Wolbachia* in cells in response to *ATPSβ1* depletion. The scale bar is 10 µm. **(N)** *Wolbachia* fluorescence intensity in W+ cells transfected with dsATPSβ1 vs. dsGFP at 72 hpt (two-sided t test, n = 20 per group, *P* = 5.214100 × 10^-18^). **(O)** Schematic of *Wolbachia* density detection and VEEV replication assays in W+ cells after *ATPSβ1* overexpression. **(P)** *Wolbachia* density (left panel; two-sided t test, n = 6 per group; *P* = 7.109200 × 10^-7^) and VEEV gRNA levels (right panel; two-sided t test, n = 6 per group; *P* = 0.000147) in *ATPSβ1*-overexpressing W+ cells at 24 hpt with the VEEV replicon. **(Q)** Schematic of *Wolbachia* density detection and DENV replication assays in W+ cells following upregulation of *ATPSβ1* expression. **(R)** *Wolbachia* density (left panel; two-sided t test, n = 6 per group; *P* = 0.000070) and DENV gRNA levels (right panel; two-sided t test, n = 6 per group; *P* = 0.005640) in *ATPSβ1*-upregulated W+ cells at 48 hpt with the DENV replicon. **(S)** Schematic of *Wolbachia* density detection and VEEV replication assays in W+ cells following *ATPSβ1* knockdown. **(T)** *Wolbachia* density (left panel; two-sided t test, n = 6 per group; *P* = 0.000381) and VEEV gRNA levels (right panel; two-sided t test, n = 6 per group; *P* = 8.531300 × 10^-9^) in *ATPSβ1* knockdown W+ cells at 24 hpt with the VEEV replicon. **(U)** Schematic of *Wolbachia* density detection and DENV replication assays in W+ cells following *ATPSβ1* downregulation. **(V)** *Wolbachia* density (left panel; two-sided t test, n = 6 per group; *P* = 5.778500 × 10^-9^) and DENV gRNA levels (right panel; two-sided t test, n = 6 per group; *P* = 0.003248) in *ATPSβ1-*downregulated W+ cells at 48 hpt with the DENV replicon. The horizontal lines within the scatter plots (**D**, **G**, **K and N**) follow the same definitions as in Fig 1. The vertical lines within the scatter plots (**P**, **R, T and V**) from right to left indicate Q1, median, and Q3. The dsRLUC treated cells served as the control group in plots (**S**, **T**, **U** and **V**). pIB/V5-GFP(W-): W- cell transfected with pIB/V5-GFP1; pIB/V5-GFP(W+): W+ cell transfected with pIB/V5-GFP; pIB/V5-ATPSβ1(W+): W+ cell transfected with pIB/V5-ATPSβ1; dsGFP(W-): W- cell transfected with dsGFP1; dsGFP(W+): W+ cell transfected with dsGFP; dsATPSβ1(W+): W+ cell transfected with dsATPSβ1. The error bars indicate the SEMs. F indicates the fold change value. ****P < 0.0001, ***P < 0.001, **P < 0.01, *P < 0.05.

Because *Wolbachia* density positively correlates with antiviral efficacy, increased *Wolbachia* density would strengthen MBV inhibition [44–47]. Indeed, in *ATPSβ1*-upregulated W+ cells, the *Wolbachia* density increased 2.35-fold, and the VEEV gRNA level decreased 3.25-fold at 24 hpt (Figs 3O and 3P). Similarly, during DENV replication, the *Wolbachia* density increased 3.59-fold, whereas the DENV gRNA decreased 3.91-fold at 48 hpt (Figs 3Q and 3R). Conversely, *ATPSβ1* downregulation in W+ cells reduced *Wolbachia* density 3.49-fold and increased VEEV gRNA 3.41-fold at 24 hpt (Figs 3S and 3T). During DENV replication, the *Wolbachia* density decreased 3.57-fold, and viral gRNA increased 2.64-fold in *ATPSβ1*-depleted W+ cells at 48 hpt (Figs 3U and 3V). Taken together, these data support that *Wolbachia*-induced expression of *ATPSβ1* increases cellular ATP levels, facilitates *Wolbachia* proliferation, and thereby inhibits MBV replication in *Ae. aegypti* cells (Fig 3).

### MBV induces ATPSβ1 to promote viral binding and intracellular ATP production, increasing infection and replication in *Wolbachia*-free *Ae. aegypti* cells

We further investigated the role of *ATPSβ1* in MBV infection of W- cells. Surprisingly, a 2.18-fold *ATPSβ1* overexpression via the pIB/V5-ATPSβ1 plasmid in W- cells showed no significant effect on cell viability but exhibited a significant increase in VEEV infection by 1.36-fold (MOI = 0.1) at 12 hpi and even caused a 2.45-fold increase in the VEEV level at a low MOI of 0.05 (Figs 4A, 4B, S2C, and S3C). Similarly, DENV infection levels were significantly elevated by 1.45-fold (MOI = 0.1) and 2.70-fold (MOI = 0.01) in *ATPSβ1*-overexpressing cells at 24 hpi (Figs 4C, 4D, and S2C). Conversely, depletion of *ATPSβ1* via dsRNA in W- cells consistently reduced VEEV and DENV infection across each MOI tested (Figs 4E-4H, S2D). Notably, a 90.37% reduction in *ATPSβ1* expression without significant effect on cell viability caused suppression of DENV infection by 5.76-fold (MOI = 0.01) at 24 hpi and VEEV infection by 3.11-fold at 12 hpi (Figs 4F, 4H, and S3D). Together, these findings demonstrate that *ATPSβ1* functions as an MBV infection enhancer in *Ae. aegypti* cells in the absence of *Wolbachia*, in stark contrast to its role as an antiviral factor that inhibits MBV infection in *Wolbachia*-infected *Ae. aegypti* cells (Fig 4)*.*

**Fig 4:**
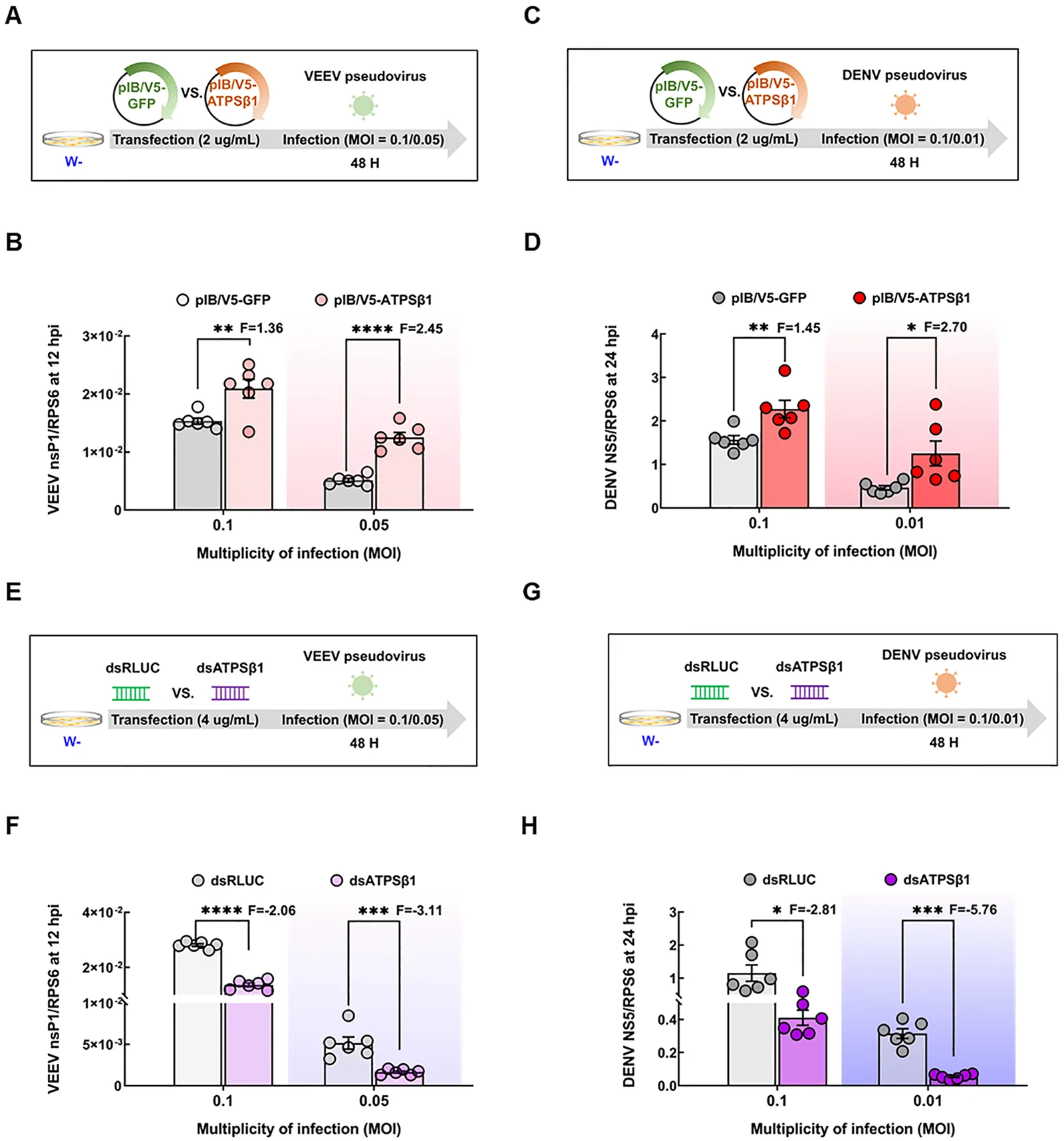
*ATPSβ1* promotes MBV infections in *Wolbachia*-free *Ae. aegypti* cells. **(A)** Schematic of the VEEV infection assay in W- cells following *ATPSβ1* overexpression. **(B)** VEEV infection levels in W- cells transfected with pIB/V5-ATPSβ1 vs. pIB/V5-GFP at 12 hpi (two-sided t test, n = 6 per group; MOI = 0.1: *P* = 0.008586; MOI = 0.05: *P* = 0.000012). **(C)** Schematic of the DENV infection assay in W- cells following *ATPSβ1* overexpression. **(D)** DENV infection levels in W- cells transfected with pIB/V5-ATPSβ1 vs. pIB/V5-GFP at 24 hpi (two-sided t test, n = 6 per group; MOI = 0.1: *P* = 0.009908; MOI = 0.01: *P* = 0.038415). **(E)** Schematic of the VEEV infection assay in W- cells following *ATPSβ1* knockdown. **(F)** VEEV infection levels in W- cells transfected with dsATPSβ1 vs. dsRLUC at 12 hpi (two-sided t test, n = 6 per group; MOI = 0.1: *P* = 7.934500 × 10^-9^; MOI = 0.05: *P* = 0.000916). **(G)** Schematic of the DENV infection assay in W- cells after *ATPSβ1* knockdown. **(H)** DENV infection levels in W- cells transfected with dsATPSβ1 vs. dsRLUC at 24 hpi (two-sided t test, n = 6 per group; MOI = 0.1: *P* = 0.028078; MOI = 0.01: *P* = 0.000215). The error bars indicate the SEMs. F indicates the fold change value. ****P < 0.0001, ***P < 0.001, **P < 0.01, *P < 0.05.

To elucidate the opposing effects of *ATPSβ1* on MBV infections in W*+* and W*-* cells, the potential binding interaction between *Ae. aegypti* ATPSβ1 and the envelope (E) protein of VEEV or DENV was predicted using AlphaFold3 and HDOCK. This approach was based on a report that cell-surface-localized ATPSβ1 in *Ae. albopictus* cells can bind to DENV structural proteins [48], and on immunofluorescence images showing wide distribution of ATPSβ1 fluorescence toward the cell periphery in both W- and W+ cells (S4 Fig). This broader pattern exceeded the distribution expected if ATPSβ1 were localized exclusively to the inner mitochondrial membrane (S4 Fig). These images support the possibility that cell-membrane localization of ATPSβ1 could facilitate its interaction with viral envelope proteins. The predicted ATPSβ1-VEEV E2 complex yielded a docking score of -235.98 and a confidence score of 0.8205, whereas the ATPSβ1-DENV E complex has a docking score of -240.27 and a confidence score of 0.8902. These results (docking score below -200 and confidence score above 0.7) indicate high confidence in the overall complex structure and the predicted protein‒protein interface [49,50], suggesting reliable binding between ATPSβ1 and VEEV or DENV through their respective E proteins. Specifically, ATPSβ1 was predicted to interact with VEEV E2 glycoprotein at GLU148, ASP157, ASN160, and ASP263 within the β-ribbon connector region (residues 230–248), and ARG337 in the domain C region (Figs 5A, 5B, and S5). These two surface-exposed regions of E2 are critical for host receptor binding and antigen recognition [26,51]. All the binding residues are fully conserved in the epizootic VEEV subtypes IAB and IC, the highly pathogenic lineages capable of infecting both horses and humans. (Figs 5A, 5B, and S5). In addition, ATPSβ1 was predicted to bind DENV E protein at SER431, LEU436, and HIS437 within the stem region (residues 395–450), a critical domain for flavivirus virion assembly and membrane fusion conformational rearrangement [52], as well as ASN194 in the Ectodomain II (EDII, residues 53–131 and 194–279, Figs 5A, 5B, and S5), the core domain responsible for DENV E protein dimerization and antigenic recognition [53,54]. These binding residues are strictly conserved across all six DENV-2 genotypes, including the globally prevalent Asian I, Asian II, Cosmopolitan, American, American-Asian, and sylvatic lineages. (Figs 5A, 5B, and S5).

**Fig 5:**
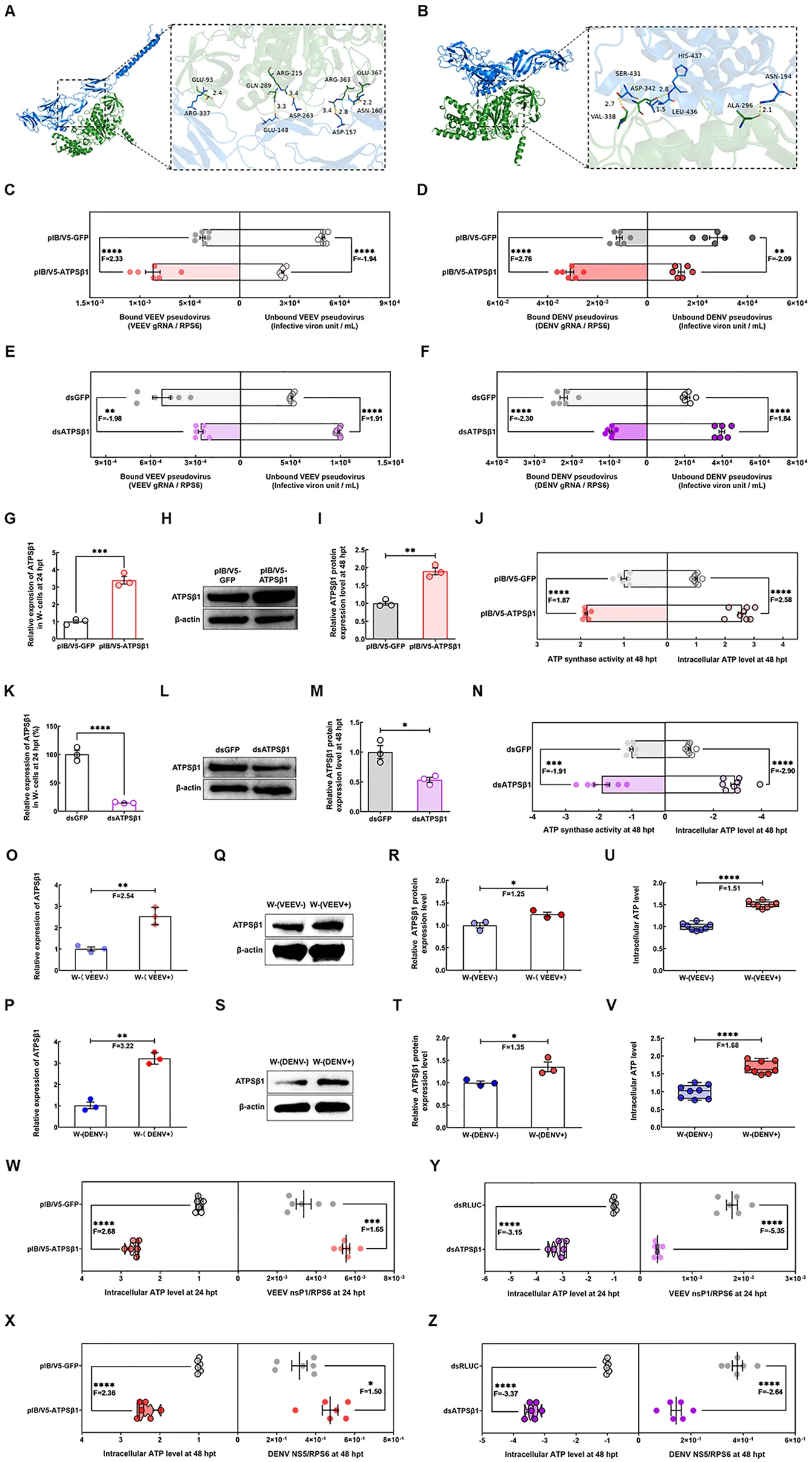
MBV-induced ATPSβ1 promotes viral binding and intracellular ATP production to increase viral infection in *Wolbachia*-free *Ae. aegypti* cells. **(A)** Structure of the ATPSβ1 (green) and VEEV E2 protein (blue) complex predicted by AlphaFold3 and HDOCK. The zoomed-in view shows key residues at the ATPSβ1–VEEV E2 binding interface and the hydrogen-bond distances (unit: Å) between adjacent residue pairs. **(B)** Structure of the ATPSβ1 (green) and DENV E protein (blue) complex predicted by AlphaFold3 and HDOCK. The residues at the ATPSβ1–DENV E binding interface and the hydrogen-bond distances between adjacent residue pairs are presented in the dashed box. **(C)** Cell-bound VEEV levels (left panel; two-sided t test, n = 6 per group; *P* = 0.000076) and unbound VEEV levels in the supernatant (right panel; two-sided t test, n = 6 per group; *P* = 5.522100 × 10^-10^) in W- cells transfected with pIB/V5-ATPSβ1 vs. pIB/V5-GFP. **(D)** Cell-bound DENV levels in W- cells (left panel; two-sided t test, n = 6 per group; *P* = 0.000001) and unbound DENV levels in the supernatant (right panel; two-sided t test, n = 6 per group; *P* = 0.002044) following ATPSβ1 overexpression. **(E)** Cell-bound VEEV levels (left panel; two-sided t test, n = 6 per group; *P* = 0.006676) in W- cells and unbound VEEV levels in the supernatant (right panel; two-sided t test, n = 6 per group; *P* = 2.582700 × 10^-12^) following ATPSβ1 knockdown. **(F)** Cell-bound DENV levels (left panel; two-sided t test, n = 6 per group; *P* = 3.460700 × 10^-7^) in W- cells and unbound virus levels in the supernatant (right panel; two-sided t test, n = 6 per group; *P* = 0.000002) following ATPSβ1 knockdown. **(G)** *ATPSβ1* mRNA levels in W- cells post-*ATPSβ1* overexpression at 24 hpt (two-sided t test, n = 3 per group, *P* = 0.000434). **(H)** Representative western blot images of ATPSβ1 protein in W- cells in response to *ATPSβ1* overexpression at 48 hpt. **(I)** Quantification of ATPSβ1 protein levels in W- cells transfected with pIBV5-ATPSβ1 vs. pIBV5-GFP at 48 hpt (two-sided t test, n = 3 per group; *P* = 0.001312). **(J)** ATP synthase activity (left panel; two-sided t test, n = 6 per group; *P* = 2.274000 × 10^-7^) and intracellular ATP levels (right panel; two-sided t test, n = 8 per group; *P* = 0.000002) in W- cells following *ATPSβ1* overexpression at 48 hpt. **(K)** Differences in *ATPSβ1* mRNA expression levels after *ATPSβ1* knockdown in W- cells at 24 hpt (two-sided t test; n = 3 per group, *P* = 0.000025). **(L)** Representative western blot images of ATPSβ1 protein in W- cells in response to *ATPSβ1* depletion at 48 hpt. **(M)** Quantification of ATPSβ1 protein levels in W- cells transfected with dsATPSβ1 vs. dsGFP at 48 hpt (two-sided t test, n = 3 per group; *P* = 0.016040). **(N)** ATP synthase activity (left panel; two-sided t test, n = 6 per group; *P* = 0.000856) and intracellular ATP levels (right panel; two-sided t test, n = 8 per group; *P* = 2.132100 × 10^-9^) in W- cells at 48 hpt with *ATPSβ1* knockdown. **(O)** *ATPSβ1* mRNA levels in W- cells infected with inactivated (VEEV-) or VEEV pseudovirus (VEEV+) at 12 hpi (two-sided t test, n = 3 per group, *P* = 0.002127). **(P)** *ATPSβ1* mRNA levels in W- cells infected with inactivated (DENV-) or DENV pseudovirus (DENV+) at 24 hpi (two-sided t test, n = 3 per group, *P* = 0.001648). **(Q)** Representative western blot images of ATPSβ1 protein in W- cells infected with VEEV+ vs. VEEV- at 24 hpi. **(R)** Quantified ATPSβ1 protein levels in W- cells in the VEEV- and VEEV+ groups at 24 hpi. (two-sided t test, n = 3 per group, *P* = 0.029858). **(S)** Representative western blot images of ATPSβ1 protein in W- cells in the DENV- and DENV+ groups at 48 hpi. **(T)** Quantified ATPSβ1 protein levels in the DENV- and DENV+ groups at 48 hpi (two-sided t test, n = 3 per group, *P* = 0.036092). **(U)** Intracellular ATP levels in W- cells post-VEEV infection at 24 hpi (two-sided t test, n = 8 per group, *P* = 2.730100 × 10^-9^). **(V)** Intracellular ATP levels in W- cells post-DENV infection at 48 hpi (two-sided t test, n = 8 per group, *P* = 0.000004). **(W)** Intracellular ATP levels (left panel; two-sided t test, n = 6 per group; *P* = 1.289500 × 10^-10^) and VEEV gRNA levels (right panel; two-sided t test, n = 6 per group; *P* = 0.000461) in W- cells following ATPSβ1 overexpression at 24 hpt. **(X)** Intracellular ATP levels (left panel; two-sided t test, n = 6 per group; *P* = 0.000020) and DENV gRNA levels (right panel; two-sided t test, n = 6 per group; *P* = 0.018364) in W- cells following ATPSβ1 overexpression at 48 hpt. **(Y)** Intracellular ATP levels (left panel; two-sided t test, n = 6 per group; *P* = 1.288100 × 10^-10^) and VEEV gRNA levels (right panel; two-sided t test, n = 6 per group; *P* = 0.000020) in W- cells following ATPSβ1 knockdown at 24 hpt. **(Z)** Intracellular ATP levels (left panel; two-sided t test, n = 6 per group; *P* = 1.764500 × 10^-12^) and DENV gRNA levels (right panel; two-sided t test, n = 6 per group; *P* = 0.000007) in W- cells following ATPSβ1 knockdown at 48 hpt. Box plots (**U** & **V**) follow the definitions in Fig 3D. The vertical lines (from right to left) within the left panel of the scatter plots (**W**, **X**, **Y**, and **Z**) indicate Q1, median, and Q3. The error bars indicate the SEMs. F indicates the fold change value. ****P < 0.0001, ***P < 0.001, **P < 0.01, *P < 0.05.

To verify the role of ATPSβ1 in viral binding, viral binding assays were performed in W- cells. *ATPSβ1* overexpression via pIB/V5-ATPSβ1 significantly increased cell-bound VEEV and DENV, while reducing the amount of unbound virus in the supernatants (Figs 5C and 5D). Conversely, depletion of *ATPSβ1* via dsATPSβ1 decreased the amount of VEEV or DENV bound to cells, with a corresponding increase in free virus in the supernatant (Figs 5E and 5F). These findings further confirm that ATPSβ1 serves as a receptor-like protein that promotes the binding of DENV and VEEV to *Ae. aegypti* cells.

Moreover, the role of *ATPSβ1* in ATP production in W- cells was evaluated. An increase in ATPSβ1 expression at both *ATPSβ1* mRNA and protein levels via pIB/V5-ATPSβ1 resulted in significant increases in ATP synthase activity and intracellular ATP levels at 48 hpt (Figs 5G-5J). Conversely, dsRNA-mediated suppression of *ATPSβ1* expression at both the transcriptional and translational levels markedly decreased ATP synthase activity and intracellular ATP levels (Figs 5K-5N). These results indicate that *ATPSβ1* increases ATP synthase activity and intracellular ATP levels in *Ae. aegypti* cells, regardless of the presence or absence of *Wolbachia*.

Next, we investigated alterations in *ATPSβ1* expression in response to MBV infections in W*-* cells. Comparisons between MBV-infected and uninfected W- cells revealed that *ATPSβ1* mRNA expression increased at 12 hpi with VEEV and at 24 hpi with DENV (Figs 5O and 5P). Consistent with these results, ATPSβ1 protein levels and intracellular ATP levels were increased at 24 hpi with VEEV or 48 hpi with DENV (Figs 5Q-5V). Taken together, these findings suggest that MBV infections induce *ATPSβ1* expression, which in turn increases intracellular ATP levels.

The effect of upregulated *ATPSβ1* expression on MBV replication was further examined. In W- cells, when *ATPSβ1* overexpression via the pIB/V5-ATPSβ1 plasmid increased intracellular ATP production, the viral gRNA levels were significantly increased in both VEEV and DENV replication assays (Figs 5W and 5X). In contrast, suppression of *ATPSβ1* by dsATPSβ1 reduced intracellular ATP levels, which was accompanied by a corresponding 5.35-fold decrease in VEEV gRNA and a 2.64-fold reduction in DENV gRNA (Figs 5Y and 5Z).

Collectively, these findings confirm that MBV-induced ATPSβ1 plays a dual role in *Wolbachia*-free *Ae. aegypti* cells by facilitating viral binding and increasing ATP production, thereby enhancing infection (Fig 5).

### *Wolbachia* overrides ATPSβ1-mediated viral binding to inhibit MBV infection

Given that MBV infection induces ATPSβ1 to promote viral infection by increasing viral binding, whereas *Wolbachia* induces ATPSβ1 but suppresses MBV infection, we speculated that *Wolbachia* might modulate the role of *ATPSβ1* in viral binding. To test this hypothesis, viral binding assays were performed in W- and W+ cells following *ATPSβ1* overexpression (Figs 6A and 6B). In both cell lines, overexpression of *ATPSβ1* significantly increased cell-bound VEEV and DENV compared with those in the controls (Figs 6C and 6D). Nevertheless, *Wolbachia* strongly inhibited MBV binding, as the levels of cell-bound virus in W+ cells were reduced by 2.55-fold for VEEV and 5.67-fold for DENV compared with those in W- cells (Figs 6C and 6D). Notably, even when *ATPSβ1*-mediated enhancement of viral binding and *Wolbachia*-mediated suppression of viral binding occurred together, W+ cells still had lower levels of cell-bound virus than W- cells (Figs 6C and 6D), suggesting that *Wolbachia*-mediated suppression of viral binding plays a major role. Conversely, the titer of unbound VEEV and DENV in the supernatant was reduced in *ATPSβ1*-upregulated groups relative to their respective controls in both cell lines (Figs 6E and 6F). Additionally, the unbound viral titer in the supernatant of W+ cells was significantly higher than that in W- cells (Figs 6E and 6F), consistent with the *ATPSβ1*-mediated enhancement of viral binding and *Wolbachia*-mediated suppression of viral binding observed above.

**Fig 6:**
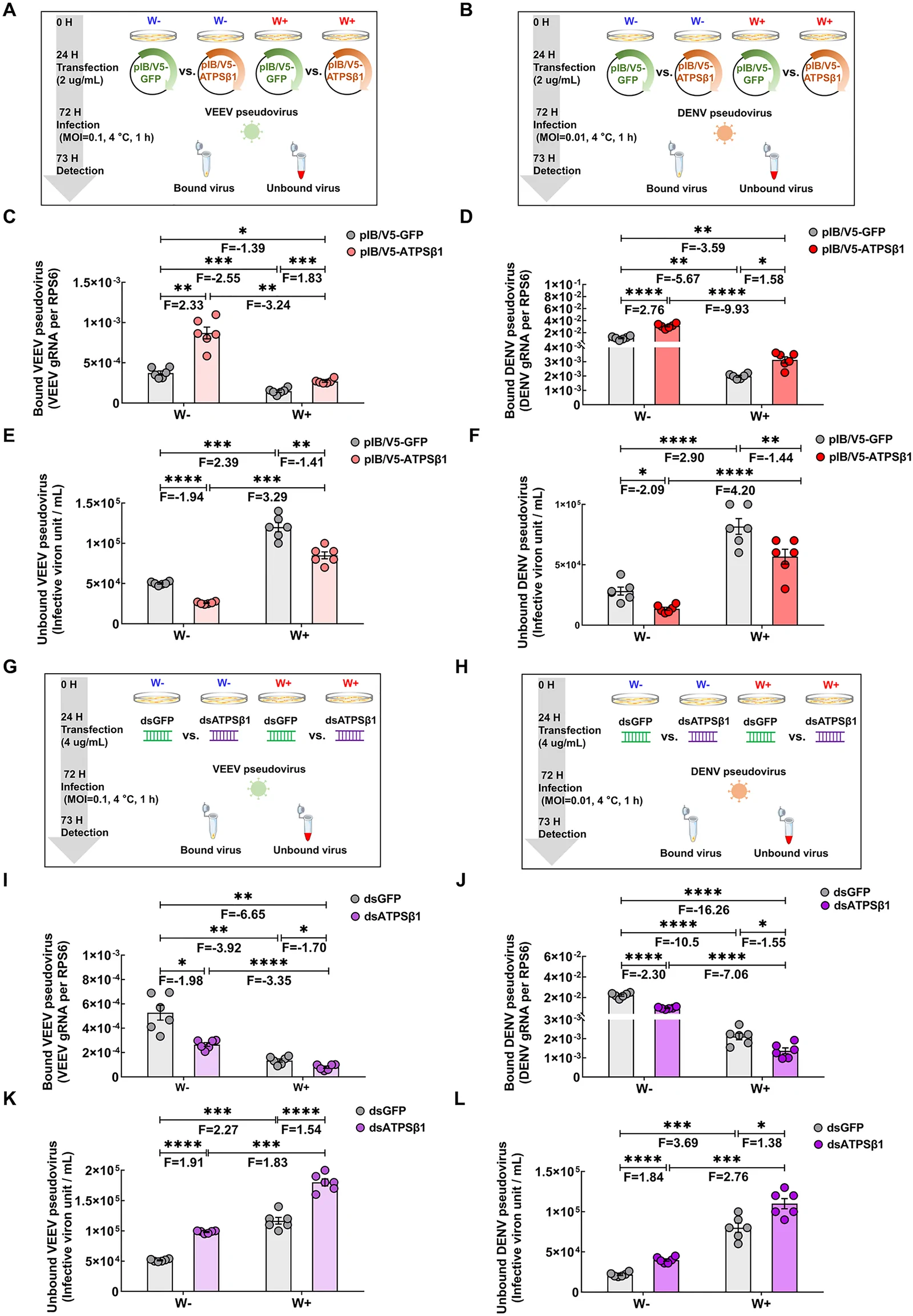
*Wolbachia* strongly inhibits MBV binding, even when ATPSβ1 increases MBV binding in both W- and W + *Ae. aegypti* cells. **(A)** Schematic of VEEV binding assays performed in W− and W+ cells following *ATPSβ1* overexpression. **(B)** Schematic of DENV binding assays performed in W− and W+ cells following *ATPSβ1* overexpression. **(C)** Cell-bound VEEV levels in W- and W+ cells transfected with pIB/V5-ATPSβ1 or pIB/V5-GFP (one-way ANOVA, n = 6 per group; W+(pIB/V5-ATPSβ1) vs. W+(pIB/V5-GFP): *P* = 0.000545; W-(pIB/V5-ATPSβ1) vs. W-(pIB/V5-GFP): *P* = 0.003574; W+(pIB/V5-GFP) vs. W-(pIB/V5-GFP): *P* = 0.000325; W+(pIB/V5-ATPSβ1) vs. W-(pIB/V5-ATPSβ1): *P* = 0.002195; W+(pIB/V5- ATPSβ) vs. W-(pIB/V5-GFP): *P* = 0.041724). **(D)** Cell-bound DENV levels in W- and W+ cells transfected with pIB/V5-ATPSβ1 or pIB/V5-GFP (one-way ANOVA, n = 6 per group; W+(pIB/V5-ATPSβ1) vs. W+(pIB/V5-GFP): *P* = 0.012555; W-(pIB/V5-ATPSβ1) vs. W-(pIB/V5-GFP): *P* = 0.000014; W+(pIB/V5-GFP) vs. W-(pIB/V5-GFP): *P* = 0.003259; W+(pIB/V5-ATPSβ1) vs. W-(pIB/V5-ATPSβ1): *P* = 0.000044; W+(pIB/V5- ATPSβ) vs. W-(pIB/V5-GFP): *P* = 0.005166). **(E)** Unbound VEEV titer in the supernatant (one-way ANOVA, n = 6 per group; W+(pIB/V5-ATPSβ1) vs. W+(pIB/V5-GFP): *P* = 0.004935; W-(pIB/V5-ATPSβ1) vs. W-(pIB/V5-GFP): *P* = 1.965000 × 10^-8^; W+(pIB/V5-GFP) vs. W-(pIB/V5-GFP): *P* = 0.000322; W+(pIB/V5-ATPSβ1) vs. W-(pIB/V5-ATPSβ1): *P* = 0.000170; W+(pIB/V5-ATPSβ1) vs. W-(pIB/V5-GFP): *P* = 0.002062). **(F)** Unbound DENV titer in the supernatant (one-way ANOVA, n = 6 per group; W+(pIB/V5-ATPSβ1) vs. W+(pIB/V5-GFP): *P* = 0.001545; W-(pIB/V5-ATPSβ1) vs. W-(pIB/V5-GFP): *P* = 0.044067; W+(pIB/V5-GFP) vs. W-(pIB/V5-GFP): *P* = 1.594800 × 10^-7^; W+(pIB/V5-ATPSβ1) vs. W-(pIB/V5-ATPSβ1): *P* = 0.000004; W+(pIB/V5-ATPSβ1) vs. W-(pIB/V5-GFP): *P* = 0.000466). **(G)** Schematic of VEEV binding assays performed in W− and W+ cells following *ATPSβ1* Knockdown. **(H)** Schematic of DENV binding assays performed in W− and W+ cells following *ATPSβ1* knockdown. **(I)** Cell-bound VEEV in W- and W+ cells transfected with dsATPSβ1 or dsGFP (one-way ANOVA, n = 6 per group; W+(dsATPSβ1) vs. W+(dsGFP): *P* = 0.044210; W-(dsATPSβ1) vs. W-(dsGFP): *P* = 0.039394; W+(dsGFP) vs. W-(dsGFP): *P* = 0.006233; W+(dsATPSβ1) vs. W-(dsATPSβ1): *P* = 0.000032; W+(ds ATPSβ) vs. W-(dsGFP): *P* = 0.003708). **(J)** Cell-bound DENV levels in W- and W+ cells transfected with dsATPSβ1 or dsGFP (one-way ANOVA, n = 6 per group; W+(dsATPSβ1) vs. W+(dsGFP): *P* = 0.048271; W-(dsATPSβ1) vs. W-(dsGFP): *P* = 0.000056; W+(dsGFP) vs. W-(dsGFP): *P* = 0.000019; W+(dsATPSβ1) vs. W-(dsATPSβ1): *P* = 0.000006; W+(ds ATPSβ) vs. W-(dsGFP): *P* = 0.000018). **(K)** Unbound VEEV titer in the supernatant following ATPSβ1 knockdown (one-way ANOVA, n = 6 per group; W+(dsATPSβ1) vs. W+(dsGFP): *P* = 0.000080; W-(dsATPSβ1) vs. W-(dsGFP): *P* = 1.786700 × 10^-11^; W+(dsGFP) vs. W-(dsGFP): *P* = 0.000387; W+(dsATPSβ1) vs. W-(dsATPSβ1): *P* = 0.000144; W+(dsATPSβ1) vs. W-(dsGFP): *P* = 0.000015). **(L)** Unbound DENV titer in the supernatant following ATPSβ1 knockdown (one-way ANOVA, n = 6 per group; W+(dsATPSβ1) vs. W+(dsGFP): *P* = 0.034105; W-(dsATPSβ1) vs. W-(dsGFP): *P* = 0.000023; W+(dsGFP) vs. W-(dsGFP): *P* = 0.000730; W+(dsATPSβ1) vs. W-(dsATPSβ1): *P* = 0.000369; W+(dsATPSβ1) vs. W-(dsGFP): *P* = 0.000146). The error bars indicate the SEMs. F indicates the fold change value. ****P < 0.0001, ***P < 0.001, **P < 0.01, *P < 0.05.

Furthermore, viral binding assays were performed, followed by downregulation of *ATPSβ1* expression in W- and W+ cells (Figs 6G and 6H). *ATPSβ1* depletion reduced cell-bound VEEV and DENV in both cell lines (Figs 6I and 6J), with a corresponding increase in unbound virus in the supernatants (Figs 6K and 6L). Notably, although *Wolbachia* alone mediated a 3.92-fold inhibition of VEEV binding and a 10.5-fold suppression of DENV binding in W+ cells, along with a 2.27-fold increase in unbound VEEV and a 3.69-fold increase in unbound DENV in the supernatant compared with those in corresponding W- cells, *ATPSβ1* suppression further reduced VEEV and DENV binding in W+ cells (Figs 6I and 6J). These results indicate that even though *Wolbachia*-induced *ATPSβ1* expression promotes VEEV and DENV binding, *Wolbachia* exerts a dominant overall strong inhibitory effect on MBV binding in W+ cells (Fig 6).

### Adenosine suppresses MBV infection and replication by increasing intracellular ATP-dependent *Wolbachia w*AlbB proliferation

To exploit *ATPSβ1* for antiviral activity without inadvertently promoting viral binding in *Wolbachia-*infected *Ae. aegypti* cells, we used adenosine, an ATP metabolite that readily enters cells via adenosine receptors (Fig 7A), whereas extracellular ATP cannot enter cells via plasma membrane transporters [55]. In W+ cells, adenosine treatment increased intracellular ATP levels in a dose-dependent manner at 12 h post-treatment, with the greatest increase observed in response to 10 μM adenosine treatment (Fig 7B). Similarly, 10 μM adenosine treatment also induced intracellular ATP level in W- cells (S6A and S6B Figs). Subsequent experiments using 10 μM adenosine showed a 2.19-fold increase in *Wolbachia* density by qPCR (Fig 7C), and FISH analysis further confirmed stronger *Wolbachia*-specific fluorescent signals (Figs 7D and 7E). These results indicate that adenosine promotes *Wolbachia* proliferation through increased intracellular ATP levels.

**Fig 7:**
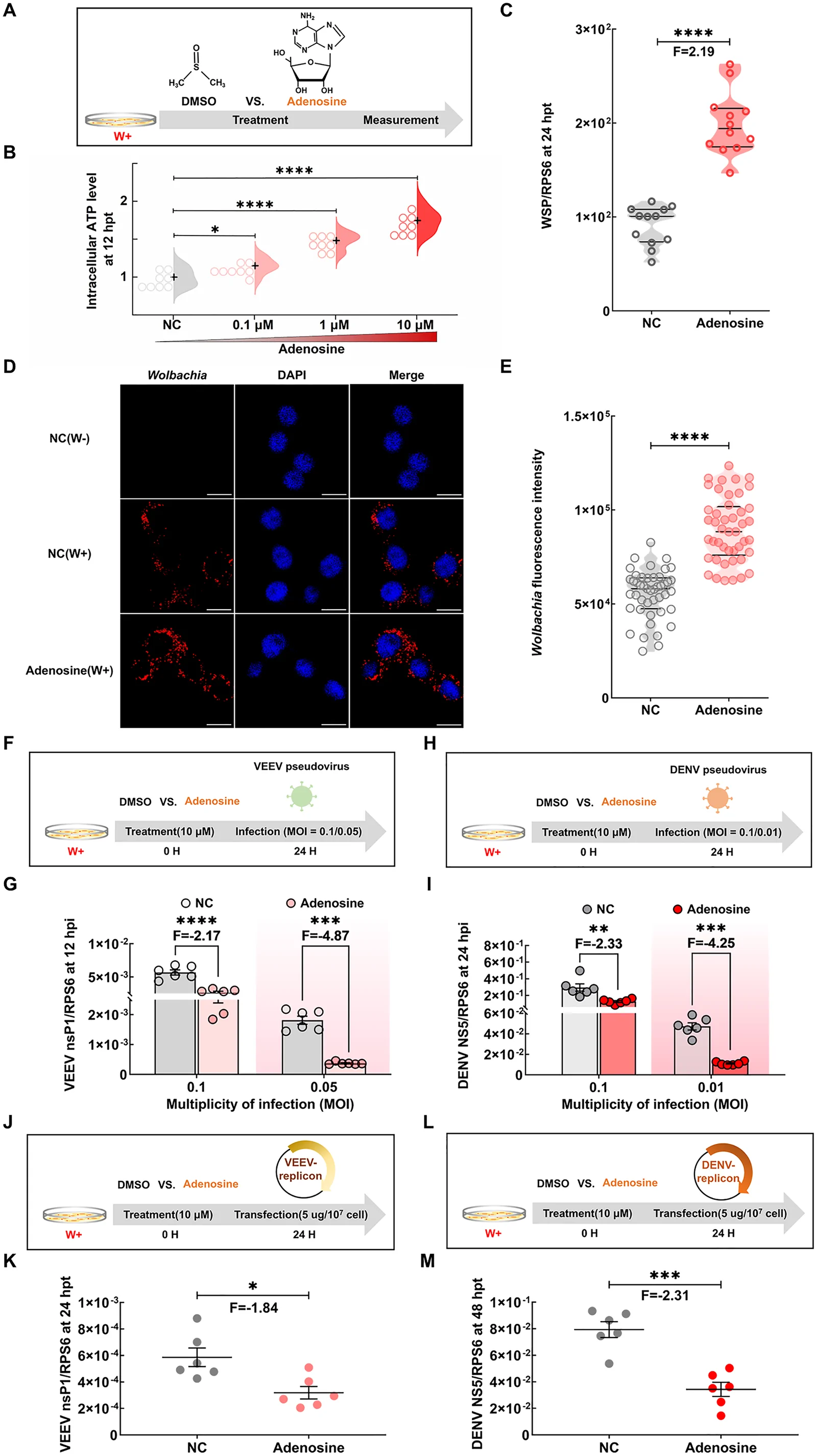
Adenosine inhibits MBV infection and replication via ATP-dependent increase in *Wolbachia w*AlbB proliferation in *Ae. aegypti* cells. **(A)** Schematic of adenosine treatment in W+ cells. **(B)** Intracellular ATP levels in W+ cells at 12 h post-treatment with the indicated adenosine concentrations (one-way ANOVA, n = 8 per group; 0.1 μM vs. negative control (NC): *P* = 0.020839; 1 μM vs. NC: *P* = 1.450400 × 10^-8^; 10 μM vs. NC: *P* = 1.024100 × 10^-12^; 1 μM vs. 0.1 μM: *P* = 0.000009; 10 μM vs. 0.1 μM: *P* = 1.706200 × 10^-10^; 10 μM vs. 1 μM: *P* = 0.000173). **(C)** *Wolbachia* density in W+ cells at 24 h post-treatment with adenosine (two-sided t test, n = 12 per group, *P* = 3.148000 × 10^-9^). **(D)** Representative images of *Wolbachia* in cells at 24 h post-treatment with adenosine vs. NC. The scale bar is 10 µm. **(E)** *Wolbachia* fluorescence intensity in W+ cells at 24 h post-treatment with adenosine vs. NC (two-sided t test, n = 45 per group, *P* = 4.490800 × 10^-17^). **(F)** Schematic of the VEEV infection assay in W+ cells following 10 μM adenosine treatment. **(G)** VEEV infection levels in W+ cells treated with adenosine or NC at 12 hpi (two-sided t test; n = 6 per group; MOI = 0.1: *P* = 0.000046; MOI = 0.05: *P* = 0.000114). **(H)** Schematic of the DENV infection assay after adenosine treatment in W+ cells. **(I)** DENV infection levels in W+ cells treated with adenosine compared with those treated with the NC at 24 hpi (two-sided t test, n = 6 per group; MOI = 0.1: *P* = 0.004087; MOI = 0.01: *P* = 0.000106). **(J)** Schematic of the VEEV replication assay in W+ cells after adenosine treatment. **(K)** VEEV gRNA levels in W+ cells treated with adenosine vs. NC at 24 hpt (two-sided t test, n = 6 per group; *P* = 0.010176). **(L)** Schematic of the DENV replication assay in W+ cells after adenosine treatment. **(M)** DENV gRNA levels in W+ cells treated with adenosine vs. NC at 48 hpt (two-sided t test, n = 6 per group, *P* = 0.000224). NC(W-): W- cells treated with DMSO as a negative control; NC(W+): W+ cells treated with DMSO as a negative control; Adenosine (W+): W+ cells treated with adenosine. The horizontal lines within the scatter plots (**C** & **E**) follow the definitions in Fig 1B. The error bars indicate the SEMs. F indicates the fold change value. ****P < 0.0001, ***P < 0.001, **P < 0.01, *P < 0.05.

We subsequently examined the effect of adenosine on MBV infection. In W+ cells, adenosine significantly reduced VEEV infection levels by 2.17-fold (MOI = 0.1) and 4.87-fold (MOI = 0.05) at 12 hpi (Figs 7F and 7G). Similarly, adenosine markedly suppressed DENV infection, resulting in 2.33-fold (MOI = 0.1) and 4.25-fold (MOI = 0.01) reductions at 24 hpi (Figs 7H and 7I). In contrast, adenosine significantly enhanced both VEEV and DENV infection in W- cells (S6C-S6F Figs). These results demonstrate that adenosine treatment effectively inhibits MBV infections in *Wolbachia*-infected *Ae. aegypti* cells, whereas adenosine-induced ATP promotes MBV infection in *Ae. aegypti* cells without *Wolbachia* symbiosis.

Moreover, we further evaluated the potency of adenosine-mediated inhibition of MBV replication. Consistent with the results described above, adenosine treatment significantly reduced both VEEV gRNA levels and DENV gRNA levels (Figs 7J-7M). Collectively, our data demonstrate that adenosine inhibits MBV infection and replication in *Wolbachia*-infected *Ae. aegypti* cells through an increase in *Wolbachia* proliferation that is dependent on increased ATP levels.

### Oligomycin inhibits MBV infection and replication in *Ae. aegypti* cells without *Wolbachia* via intracellular ATP depletion

Building on the aforementioned mechanism by which ATP promotes MBV infection, we further detect the antiviral effect of an intracellular ATP inhibitor on MBV infection in *Ae. aegypti* W- cells without *Wolbachia*. To address this question, we used oligomycin, a specific inhibitor of mitochondrial ATP synthase [56], to impair ATP synthesis in W- cells (Fig 8A). As expected, oligomycin treatment resulted in a dose-dependent reduction in intracellular ATP levels at 12 h post-treatment, with the 20 μg/mL oligomycin group exhibiting a 5.95-fold decrease compared with that in the control group (Fig 8B).

**Fig 8:**
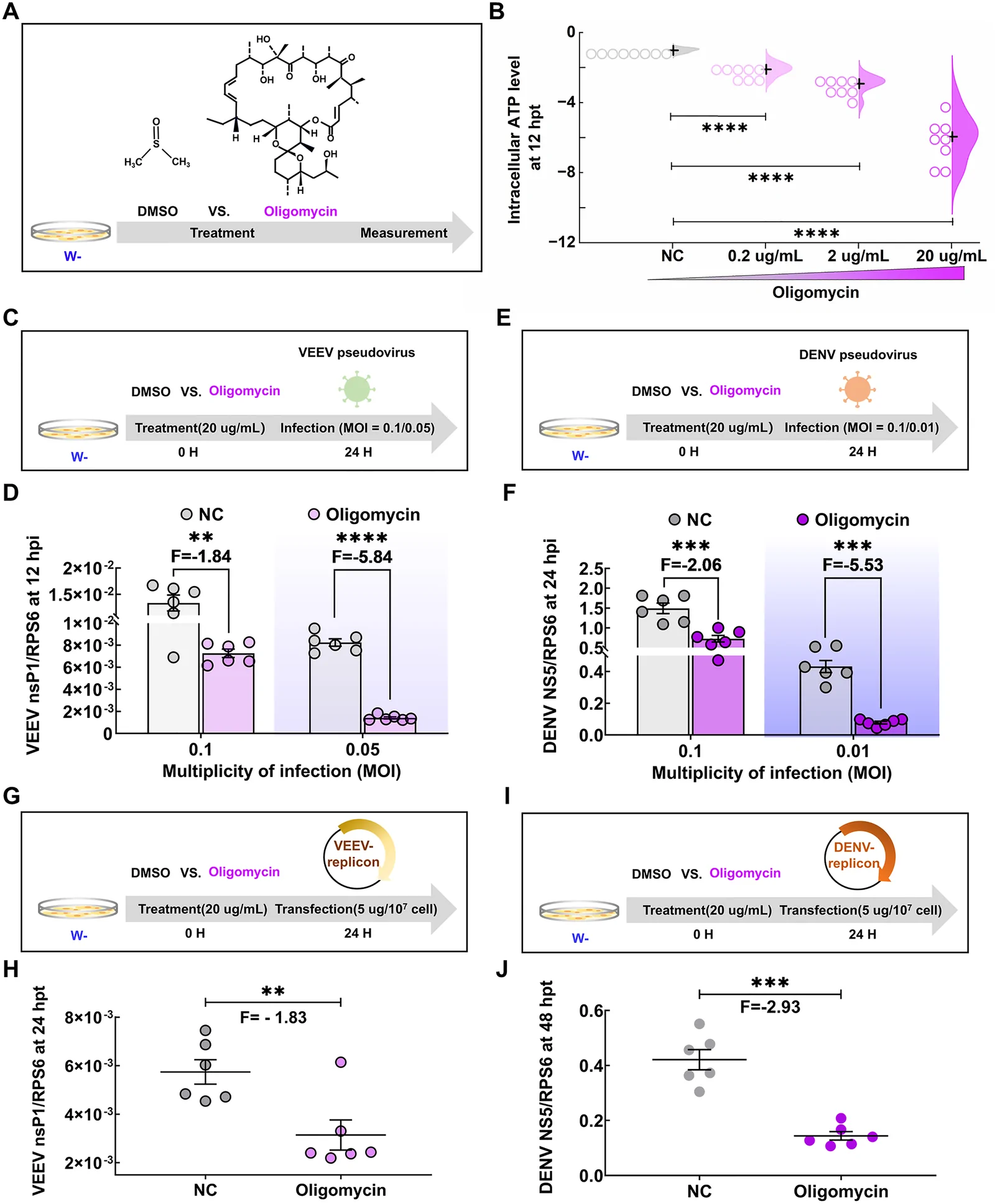
Oligomycin suppresses MBV infection and replication in *Wolbachia*-free *Ae. aegypti* cells. **(A)** Schematic of oligomycin treatment in W- cells. **(B)** Intracellular ATP levels in W- cells at 12 h post-treatment with the indicated concentrations of oligomycin (one-way ANOVA, n = 8 per group; 0.2 µg/mL vs. NC: *P* = 0.000002; 2 µg/mL vs. NC: *P* = 8.067000 × 10^-7^; 20 µg/mL vs. NC: *P* = 1.303000 × 10^-7^; 2 µg/mL vs. 0.2 µg/mL: *P* = 0.009484; 20 µg/mL vs. 0.2 µg/mL: *P* = 0.000009; 20 µg/mL vs. 2 µg/mL: *P* = 0.000017). **(C)** Schematic of the VEEV infection assay in W- cells after oligomycin treatment. **(D)** VEEV infection levels in W- cells treated with oligomycin vs. NC at 12 hpi (two-sided t test, n = 6 per group; MOI = 0.1: *P* = 0.009058; MOI = 0.05: *P* = 0.000002). **(E)** Schematic of the DENV infection assay in W- cells after oligomycin treatment. **(F)** DENV infection levels in W- cells treated with oligomycin vs. NC at 24 hpi (two-sided t test, n = 6 per group; MOI = 0.1: *P* = 0.000595; MOI = 0.01: *P* = 0.000153). **(G)** Schematic of the VEEV replication assay in W- cells after oligomycin treatment. **(H)** VEEV gRNA levels in W- cells treated with oligomycin vs. NC at 24 hpt (two-sided t test, n = 6 per group, *P* = 0.008706). **(I)** Schematic of the DENV replication assay in W- cells following oligomycin treatment. **(J)** DENV gRNA levels in W- cells treated with oligomycin vs. NC at 48 hpt (two-sided t test, n = 6 per group, *P* = 0.000268). The error bars indicate the SEMs. F indicates the fold change value. ****P < 0.0001, ***P < 0.001, **P < 0.01.

MBV infection assays were subsequently performed following ATP reduction with 20 µg/mL oligomycin. In W- cells, oligomycin treatment significantly reduced VEEV infection levels by 1.84-fold (MOI = 0.1) and 5.84-fold (MOI = 0.05) at 12 hpi (Figs 8C and 8D). Consistently, oligomycin treatment led to 2.06-fold (MOI = 0.1) and 5.53-fold (MOI = 0.01) decreases in DENV infection levels at 24 hpi (Figs 8E and 8F). To further characterize the cell-type-specific antiviral activity of oligomycin, we extended the above assays to W+ cells. Strikingly, in contrast to the robust antiviral effect observed in W- cells, oligomycin-mediated *Wolbachia* depletion in W+ cells significantly elevated VEEV and DENV infection levels (Fig S7). These results indicate that oligomycin’s antiviral efficacy, which reduces intracellular ATP to inhibit MBV infection, follows a cell-type-specific pattern. It exerts potent inhibitory effects in *Wolbachia*-free *Ae. aegypti* cells but completely fails to suppress MBV infection in *Wolbachia*-infected cells. Furthermore, the role of oligomycin on MBV replication in *Ae. aegypti* cells was tested. Corresponding viral replication assays revealed that oligomycin led to a 1.83-fold reduction in VEEV gRNA levels at 24 hpt and a 2.93-fold reduction in DENV gRNA levels at 48 hpt (Figs 8G-8J). Collectively, these findings demonstrate that oligomycin-mediated ATP depletion inhibits both MBV infection and replication exclusively in natural *Ae. aegypti* cells without *Wolbachia*, with no antiviral effect observed in *Wolbachia*-infected *Ae. aegypti* cells.

### Adenosine promotes *Wolbachia* proliferation via increased intracellular ATP levels to inhibit MBV infection and replication in *Ae. albopictus* cells

To investigate whether the adenosine-induced increase in *Wolbachia* density exhibited a *Wolbachia* strain-specific pattern, the naturally infected *Ae. albopictus* Aa23 cells carrying *Wolbachia w*AlbB were subjected to intracellular ATP modulation assays (Fig 9A). Adenosine treatment increased intracellular ATP levels in a dose-dependent manner at 12 h post-treatment (Fig 9B). Interestingly, following adenosine-mediated ATP elevation, the density of *Wolbachia* increased 1.75-fold at 24 h post-treatment (Fig 9C). FISH results further confirmed that the fluorescence signals corresponding to total *Wolbachia* were enhanced 2.05-fold following treatment with 10 μM adenosine (Figs 9D and 9E). Together, these findings consistently demonstrate that adenosine increases the level of intracellular ATP, which in turn stimulates the proliferation of *Wolbachia* in naturally infected *Ae. albopictus* cells.

**Fig 9:**
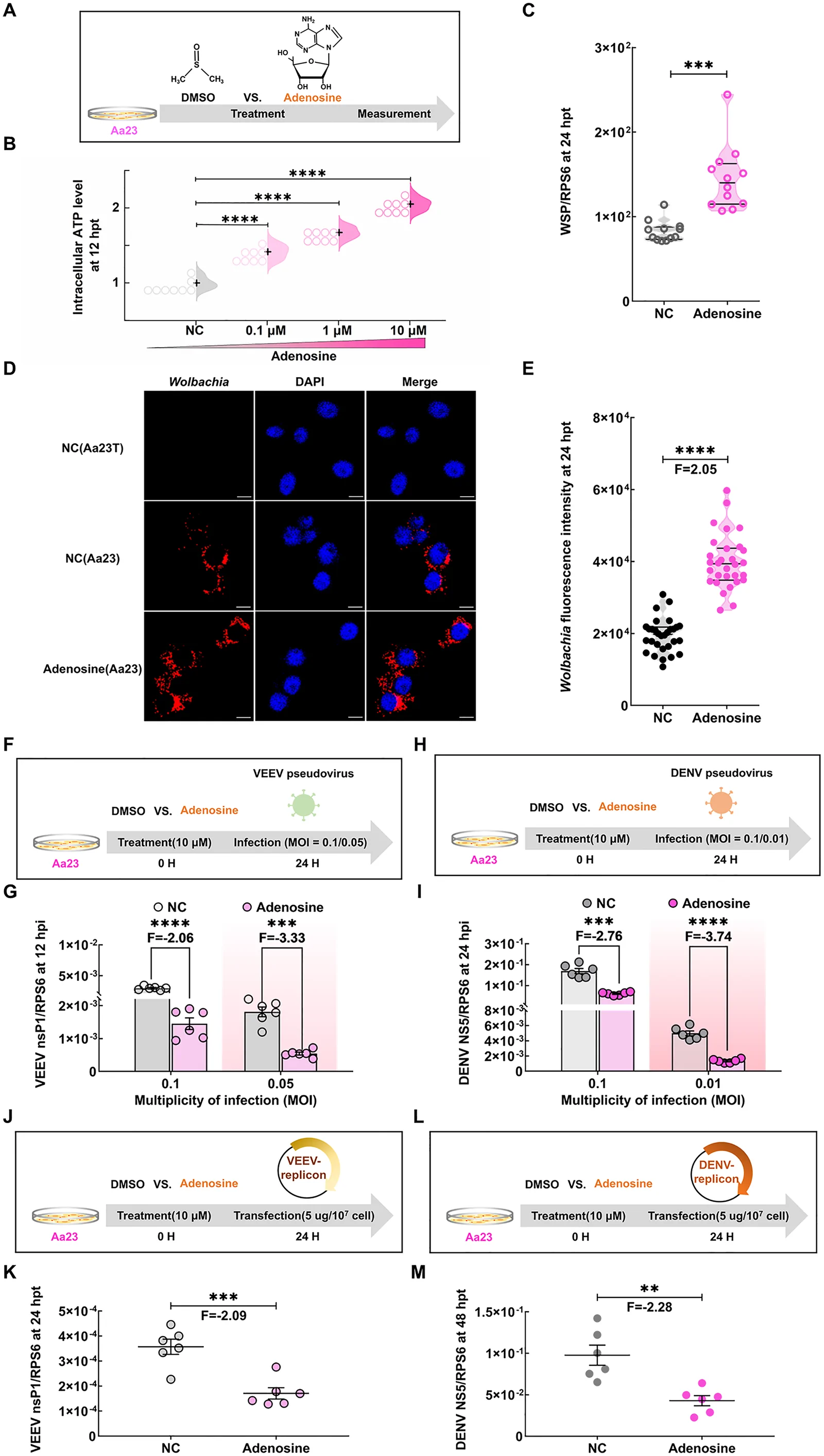
Adenosine inhibits MBV infection and replication in *Ae. albopictus* cells. **(A)** Schematic of adenosine treatment in Aa23 cells. **(B)** Intracellular ATP levels in Aa23 cells at 12 h post-treatment with the indicated concentrations of adenosine (one-way ANOVA, n = 8 per group; 0.1 μM vs. NC: *P* = 3.459700 × 10^-9^; 1 μM vs. NC: *P* = 1.474000 × 10^-14^; 10 μM vs. NC: *P* = 5.876900 × 10^-20^; 1 μM vs. 0.1 μM: *P* = 0.000002; 10 μM vs. 0.1 μM: *P* = 6.302300 × 10^-15^; 10 μM vs. 1 μM: *P* = 1.045000 × 10^-9^). **(C)** *Wolbachia* density in Aa23 cells at 24 h post-treatment with 10 μM adenosine (two-sided t test, n = 12 per group; *P* = 0.000133). **(D)** Representative fluorescence images of *Wolbachia* in Aa23 and Aa23T cells at 24 h post-treatment with adenosine vs. NC. The scale bar is 10 µm. **(E)** Total *Wolbachia* fluorescence intensity in Aa23 cells treated with adenosine vs. NC (two-sided t test, n = 30 per group, *P* = 3.097600 × 10^-18^). **(F)** Schematic of the VEEV infection assay in Aa23 cells following 10 μM adenosine treatment. **(G)** VEEV infection levels in Aa23 cells treated with adenosine vs. NC at 12 hpi (two-sided t test, n = 6 per group; MOI = 0.1: *P* = 0.000027; MOI = 0.05: *P* = 0.000187). **(H)** Schematic of the DENV infection assay in Aa23 cells after adenosine treatment. **(I)** DENV infection levels in Aa23 cells treated with adenosine compared with those treated with the NC at 24 hpi (two-sided t test, n = 6 per group; MOI = 0.1: *P* = 0.000209; MOI = 0.01: *P* = 6.102300 × 10^-7^). **(J)** Schematic of the VEEV replication assay in Aa23 cells following adenosine treatment. **(K)** VEEV gRNA levels in Aa23 cells treated with adenosine relative to those treated with the NC at 24 hpt (two-sided t test, n = 6 per group, *P* = 0.000622). **(L)** Schematic of the DENV replication assay in Aa23 cells after adenosine treatment. **(M)** DENV gRNA levels in Aa23 cells treated with adenosine vs. NC at 48 hpt (two-sided t test, n = 6 per group, *P* = 0.002390). NC(Aa23T): Aa23T cells treated with DMSO as a negative control; NC(Aa23): Aa23 cells treated with DMSO as a negative control; Adenosine (Aa23): Aa23 cells treated with adenosine. The horizontal lines within the scatter plot (E) follow the definitions in Fig 1. The error bars indicate the SEMs. F indicates the fold change value. ****P < 0.0001, ***P < 0.001, **P < 0.01.

We further determined whether the antiviral effect of adenosine is host-specific or broadly applicable. First, the effect of *Wolbachia* on VEEV infection was investigated, based on prior studies have shown multiple *Wolbachia* strains suppress MBV infection, most notably DENV infection, whereas their impact on VEEV in *Ae. albopictus* remains poorly defined [36,44]. Pseudovirus infection assays were performed in Aa23 and Aa23T cells, with the latter serving as a *Wolbachia*-negative control generated from Aa23 by removing *Wolbachia*. VEEV infection level was significantly lower in Aa23 cells relative to Aa23T cells (S8 Fig). Based on the proven inhibitory effect of *Wolbachia* on VEEV in Aa23 cells, the role of adenosine in MBV infection and replication was further characterized in Aa23 cells. The 10 μM adenosine significantly suppressed both VEEV and DENV infection. VEEV infection was reduced by 2.06-fold (MOI = 0.1) and 3.33-fold (MOI = 0.05) at 12 hpi (Figs 9F and 9G). Similarly, DENV infection decreased by 2.76-fold (MOI = 0.1) and 3.74-fold (MOI = 0.01) at 24 hpi (Figs 9H and 9I). Correspondingly, viral replicon assays revealed comparable reductions in VEEV gRNA and DENV gRNA levels (Figs 9J-9M), indicating that adenosine also restricts MBV replication. In summary, our results demonstrate that adenosine supplementation induces intracellular ATP accumulation, promotes *Wolbachia* proliferation, and thereby increases the inhibition of MBV infection and replication in natural *Ae. albopictus* cells.

## Discussion

Our study revealed that *Ae. aegypti* ATPSβ1 was hijacked by MBVs to facilitate infection in *Ae. aegypti*. ATPSβ1 acted as a receptor-like protein to increase viral binding and served as a key regulator of ATP synthesis, thereby establishing an intracellular microenvironment with elevated ATP levels. This molecule governs nearly all energy-dependent phases of viral infection. This dual function of *Ae. aegypti* ATPSβ1 ultimately enhanced the binding and replication of DENV and VEEV. Interestingly, during MBV infections, *Wolbachia* also induced *Ae. aegypti* ATPSβ1 expression but exploited it to combat MBV infections. Specifically, *Wolbachia* counteracted ATPSβ1-mediated viral binding, thereby inhibiting the binding of DENV and VEEV to *Ae. aegypti* cells. Furthermore, *Wolbachia* utilized ATPSβ1 to increase intracellular ATP synthesis and subsequently redirected ATP to support its own symbiotic proliferation. This process not only restricted MBV infection and replication but also ultimately reversed the promotional effect of the high-ATP intracellular microenvironment on these processes. On the basis of this mechanistic insight, we demonstrated that oligomycin, a specific inhibitor of mitochondrial ATP synthase, suppressed MBV infection and replication in *Wolbachia*-free *Ae. aegypti* by reducing the level of intracellular ATP. Conversely, adenosine, an ATP metabolite, significantly elevates intracellular ATP production in both *Ae. aegypti and Ae. albopictus* cells harboring *Wolbachia*, regardless of transfection or natural symbiosis. This increase in intracellular ATP enhances *Wolbachia* proliferation, which in turn strengthens the inhibition of MBV infection (Fig 10, S1 Raw Gel, and S1 Data).

**Fig 10:**
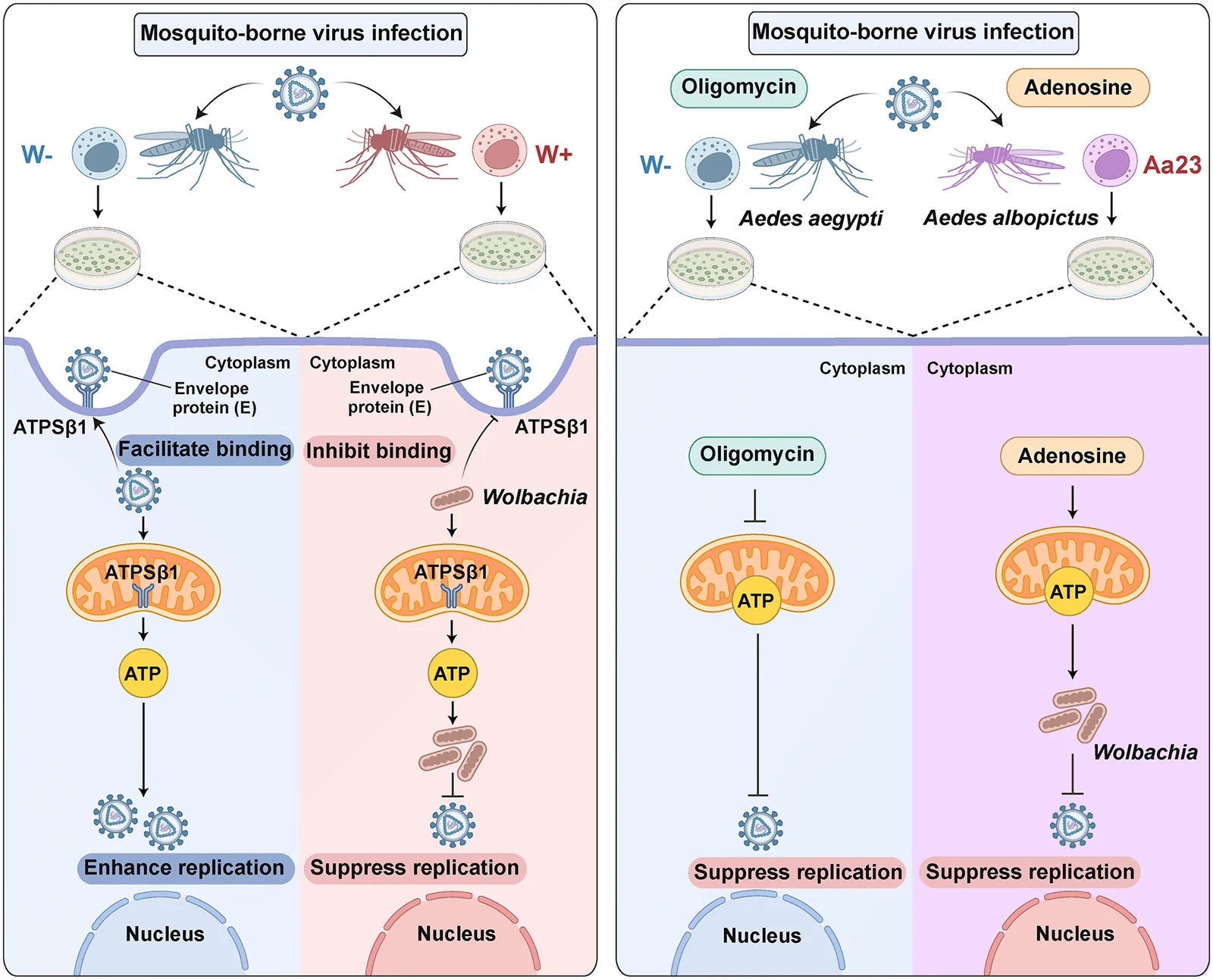
Schematic summary of key findings. The black sharp-ended arrows denote experimentally validated promoting effects, whereas the black flat-ended arrows indicate inhibitory effects confirmed in this study. W-: *Wolbachia*-free *Ae. aegypti* cell; W + : *Wolbachia*-infected *Ae. aegypti* cell; Aa23: Natural *Ae. albopictus* cell. *ATPSβ1*: ATP synthase beta subunit 1; ATP: adenosine triphosphate.

To our knowledge, the present research provides the first evidence that *Wolbachia w*AlbB significantly inhibit VEEV infection in both *Ae. aegypti* and *Ae. albopictus* cells, although the suppression or even blockage of MBV transmission by *Wolbachia* has been increasingly documented [40,57,58]. Notably, *Wolbachia w*AlbB exerted a potent inhibitory effect on VEEV, reducing both viral binding and intracellular replication even following viral entry into *Aedes* cells. These findings align with and extend current reports that *Wolbachia w*AlbB endows *Aedes* hosts with broad-spectrum resistance to MBV, including orthoflaviviruses and alphaviruses [21,24,59]. Together, our results highlight that *w*AlbB is a particularly promising *Wolbachia* strain for developing a novel *Culex* mosquito tool with a robust ability to counter MBVs, further supporting the great potential of *Wolbachia* in MBV prevention. Furthermore, this study provides critical experimental evidence for the design of novel *Wolbachia*-based vector control strategies targeting highly pathogenic VEEV. Given that most *Culex* mosquitoes are naturally infected with *Wolbachia* [60,61], future work should further investigate the efficacy of *Wolbachia*-mediated inhibition against diverse VEEV strains transmitted by *Culex* mosquitoes across different epidemic regions.

Our research revealed that ATP synthase beta subunit 1 (*ATPSβ1*, AAEL002827) serves as a hub metabolism gene supporting both DENV and VEEV early in infection in *Ae. aegypti*. Previous studies have reported that DENV activates oxidative phosphorylation in *Ae. aegypti* and employs a distinct *ATPSβ* isoform, the ATP synthase beta subunit 2 gene (*ATPSβ2*, AAEL003393), to promote viral infection [62]. However, the detailed mechanism remains poorly understood. In this study, upon infection with either DENV or VEEV, *ATPSβ1* was the most highly overexpressed at both the transcriptional and translational levels among the three known ATP synthase β-isoforms in *Ae. aegypti*, namely, *ATPSβ1*, *ATPSβ2*, and *ATPSβ3* (AAEL027636). Moreover, our results elucidated the phase-specific functions of ATPSβ1 in enhancing viral infection via its dual subcellular localization. Primarily localized to the F1 domain of ATP synthase in respiratory chain complex V on the mitochondrial inner membrane, ATPSβ1 critically regulates intracellular ATP synthesis, thereby energizing viral replication. This strategy of hijacking host ATP to facilitate viral replication is conserved across different types of viruses, including positive-sense single-stranded RNA viruses (Zika virus and West Nile virus), negative-sense single-stranded RNA viruses (influenza virus), and double-stranded DNA viruses (Epstein‒Barr virus and human papilloma virus) [63]. These findings indicate that ATPSβ1 might function as a universal replication enhancer for these major virus classes. Additionally, our data revealed that ATPSβ1, when partially trafficked to the cell membrane, acted as a viral receptor-like protein, promoting the binding of both VEEV and DENV to *Ae. aegypti* cells. The predicted ATPSβ1-binding residues in the VEEV E2 protein are fully conserved among the highly pathogenic VEEV subtypes IAB and IC, and the predicted ATPSβ1-binding residues in the DENV E protein are strictly conserved across all six DENV-2 genotypes. Notably, Leu436, one of the predicted ATPSβ1-binding residues in DENV E, has been previously documented to participate in DENV entry and spread [64]. Moreover, chikungunya virus adopts a similar binding-facilitating strategy by interacting with *Ae. albopictus* membrane-localized ATPSβ, a homolog of *Ae. aegypti* ATPSβ1 [65], indicating a conserved role for membrane-localized ATPSβ in viral binding. Collectively, our work identifies ATPSβ1 as a key host factor for the early infection phases of multiple MBVs and elucidates a biphasic mechanism whereby its subcellular localization dictates its functions in viral energy support and cellular entry, emphasizing its potential as a broad-spectrum target for antiviral intervention. However, further studies using fully replication-competent live viruses and investigating how to fine-tune ATPSβ1 expression are required to validate the strength of ATPSβ1-mediated antiviral activity throughout the entire viral infection cycle and to assess its relevance to different epidemic viral strains.

Notably, we found that even though *Wolbachia* induced host *ATPSβ1* expression upon infectious and inactive MBV infection, ultimately repurposing ATPSβ1 into a potent antiviral factor that inhibits viral replication. While *Wolbachia* is known to exploit insect host resources such as lipids, glucose, iron, and amino acids for symbiosis [28,66–68], these resources are also required for viral propagation, which depends on ATP-driven metabolism [69,70]. However, the key regulator that coordinates energy metabolism during antiviral defense remains unidentified. In this study, we revealed that *Wolbachia* strongly manipulated ATP production primarily via the key regulator *ATPSβ1* gene. Specifically, among the mitochondrial respiratory chain complex V-related genes, *Wolbachia* significantly increased ATPSβ1 expression to enhance ATP synthesis during MBV infections. Simultaneously, *Wolbachia* upregulated the expression of genes encoding ATP-binding cassette (ABC) transporters, including *AaeABCG8* (AAEL019463) and *AaeABCA7.2* (AAEL014699). These transporters function as ATP-dependent pumps that shuttle various metabolites, such as lipids, glucose, iron, and amino acids, across membranes in mammals and have previously been linked to arboviral infection in *Ae. aegypti* mosquitoes [71,72]. Although further investigation is needed to determine whether *Wolbachia* only harnesses host ABC transporters or uses its own ABC transporters to compete with MBVs for ATP, this coordinated metabolic reprogramming reflects an evolved strategy in which *Wolbachia* increases intracellular ATP levels for its own benefit while depleting ATP pools essential for MBV replication, thereby effectively restricting viral propagation. Furthermore, *Wolbachia* exerts a dominant, broad inhibitory effect on viral binding, reinforcing its overall antiviral strategy. We demonstrated that depletion of ATPSβ1 further increased *Wolbachia*-induced inhibition of both DENV and VEEV binding, even though overexpression of ATPSβ1 resulted in a slight increase in viral binding regardless of *Wolbachia* presence. These findings suggest that *Wolbachia* primarily uses ATPSβ1 as an ATP-synthesizing enhancer rather than as a viral binding protein. Taken together, these findings reveal that *Wolbachia*-induced *ATPSβ1* achieves a multilevel antiviral effect spanning genetic, proteomic, and metabolic levels by inhibiting viral binding and hijacking ATP, the energy metabolite essential for viral propagation. Given the increasing number of MBV outbreaks worldwide, how *Wolbachia* modulates *ATPSβ1*-mediated antiviral effects requires extensive validation in infectious and mock-infected experimental systems.

On the basis of this identified regulatory mechanism, our study is the first to report a link between adenosine supplementation and *Wolbachia* symbiosis, along with increased inhibition of MBV. This offers a novel and practical strategy for the prevention and control of MBV transmission. Specifically, exogenous adenosine supplementation not only significantly elevates intracellular ATP levels in both *Ae. aegypti* and *Ae. albopictus* cells, thereby facilitating *Wolbachia* symbiosis, but also inhibiting MBV replication in *Aede*s. While previous studies have reported that extracellular adenosine activates immune responses against pathogens through the adenosine signaling pathway in various species, such as *Drosophila*, *Spodoptera litura*, *Bombyx mori,* and mammals [73–76], this study highlights the particular potential of intracellular adenosine in boosting the *Wolbachia* growth by increasing intracellular ATP and subsequently reducing the vector competence of *Ae. aegypti* and *Ae. albopictus* for MBV transmission. We further propose that supplementing mosquito diets with adenosine, a metabolism enhancer compatible with the previously reported use of ATP as a phagostimulant [77], could provide an effective dietary formulation for the mass rearing of *Wolbachia*-infected *Aedes* mosquitoes*.* This approach is critical for successful population replacement and population suppression strategies aimed at collapsing wild, susceptible mosquito populations and reducing MBV prevalence. Although evaluating the long-term physiological effects of adenosine supplementation falls outside the scope of this study, it represents an important direction for future research.

This study revealed that in naturally *Wolbachia*-free *Ae. aegypti*, oligomycin, a specific inhibitor of ATP synthase [56], strongly inhibited the replication of MBVs, such as DENV and VEEV, by creating an energy-deficient microenvironment that was unfavorable for viral propagation. These results align with earlier reports that oligomycin suppresses the replication of both RNA viruses (severe acute respiratory syndrome coronavirus 2, human immunodeficiency virus, influenza A viruses, Sendai virus, and bovine enterovirus) and DNA viruses (bovine herpesvirus 1 and human cytomegalovirus), underscoring its broad-spectrum antiviral activity [78–83]. Collectively, our results indicate that oligomycin represents a promising candidate effector molecule for reducing the vector competence of naturally occurring *Ae. aegypti* in transmitting MBV. However, translating these findings into practical applications will require further investigation into the optimal dosing and effective delivery strategies for oligomycin, as well as evaluation of the long-term antiviral stability in *Aedes* mosquitoes under field conditions.

This study revealed the inhibitory effect of *Wolbachia* on VEEV infection and systematically elucidated the interaction mechanism between MBV and *Wolbachia* in *Aedes* cells. During early infection, MBVs induce the expression of the *Aedes ATPSβ1* gene to promote viral binding and subsequent replication by establishing a high-ATP microenvironment. Conversely, *Wolbachia* upregulates *ATPSβ1* expression to increase intracellular ATP production, resulting in the competitive acquisition of ATP to promote its own proliferation. This reverses the promoting effect of the high-ATP microenvironment, ultimately leading to reduced MBV binding and replication. Furthermore, these results highlight the translational potential of this antiviral mechanism. Adenosine could promote *Wolbachia* symbiosis in *Aedes* mosquitoes, increase their antiviral capacity, and aid in the development of dietary formulations for the mass rearing of *Wolbachia*-infected *Aedes* mosquitoes. Moreover, oligomycin could inhibit ATP synthesis in *Wolbachia*-free *Ae. aegypti*, effectively suppressing viral transmission. In addition, *w*AlbB *Wolbachia* possesses broad-spectrum antipathogenic properties, making it a promising candidate for constructing VEEV-resistant mosquito lines and providing a targeted strategy for controlling specific highly pathogenic MBVs.

## Materials and methods

### Ethics statement

All viral infection assays were conducted in strict accordance with protocols approved by the Biosafety and Ethics Committee for Biomedical Research of Hunan Normal University (approval nos. 2025–618, 2023–500, 2022–207, and 2018–023).

### Cells

*Wolbachia*-free Aag2 (W-), and *Wolbachia w*AlbB-infected Aag2 (W+) *Ae. aegypti* cells [25], *Wolbachia*-carrying *Ae. albopictus* (Aa23) cells, as well as *Wolbachia*-free *Ae. albopictus* (Aa23T) cells generated from Aa23 via tetracycline treatment [35] were grown in cell culture medium composed of Schneider’s Drosophila medium (Gibco, NY, USA), 10% (v/v) fetal bovine serum (FBS), and 1% (v/v) penicillin/streptomycin. All the mosquito cell lines were maintained at 26°C in a cell incubator (150i, Thermo, MA, USA). Baby hamster kidney (BHK-21) cells were cultured in Dulbecco’s modified Eagle medium (DMEM) supplemented with 10% (v/v) FBS and 1% (v/v) penicillin/streptomycin and maintained at 37°C and 5% CO_2_ in a cell culture incubator (3111, Thermo, MA, USA).

### Plasmid construction

The plasmids of the T7 promoter-driven dengue virus (DENV) serotype 2 TSV01 strain (GenBank accession number: AY037116.1) replicon containing a partial deletion of the C-prM-E genes (from 114 nt to 2233 nt) and a *Renilla* luciferase (*Rluc*) reporter gene (denoted as DENV-Rluc-rep), the vector containing the sequence of the C-prM-E gene (denoted as DENV-SP), the CMV promoter-driven Venezuelan equine encephalitis virus (VEEV) vaccine strain TC83 (GenBank accession number: L01443.1) replicon containing the enhanced green fluorescent protein (*eGFP*) reporter gene (denoted as VEEV-eGFP-rep), and the expression vector encoding the structural protein gene of VEEV (denoted as VEEV-SP) were kindly provided by Prof. Bo Zhang (Wuhan Institute of Virology, Chinese Academy of Sciences).

To generate a CMV promoter-driven DENV replicon expressing eGFP (designated DENV-eGFP-rep), we modified DENV-Rluc-rep by replacing the T7 promoter with the CMV promoter and the *Rluc* gene with the *eGFP* gene. Briefly, fragments containing the *Sac* II-CMV promoter, the 5’ terminal 210 nt sequence of the DENV genome, and the eGFP gene-*BsiW* I sequence were amplified using the VEEV-eGFP-rep and DENV-Rluc-rep plasmids as templates. These fragments were purified and subsequently fused by two rounds of overlap PCR to generate a single fragment containing the *Sac* II-CMV-DENV 5’ 210 nt-eGFP-*BsiW* I sequence. This fragment was subsequently inserted into the DENV-Rluc-rep plasmid between the restriction sites of *Sac* II and *BsiW* I **(**Fig 1D**)**.

The DENV-SP plasmid encoding the DENV structural proteins **(**Fig 1D**)** was constructed by cloning the DENV C-prM-E gene into the pCAGGS vector at the *Not* I and *Nhe* I restriction sites.

The ATP synthase β-subunit 1 (*ATPSβ1*) gene (VectorBase: AAEL002827-RA) or *GFP* gene (GenBank: X83959.1) sequences were inserted between the *Spe* I and *Not* I restriction sites located downstream of the OpIE2 promoter within the pIB/V5-His vector (HG-VKI0031, Honor Gene, Nanjing, China) to construct the pIB/V5-*ATPSβ1* and pIB/V5-*GFP* plasmids encoding *ATPSβ1* and *GFP*, respectively.

All the constructs were verified by DNA sequencing at Sangon Biotech (https://www.sangon.com).

### Pseudovirus production and titration

VEEV pseudovirus was generated by cotransfection of 1.5 µg of VEEV-eGFP-rep and 1.5 µg of VEEV-SP into BHK-21 cells (approximately 1 × 10^6^ cells) utilizing FuGENE HD Transfection Reagent (E2311, Promega, WI, USA) according to the manufacturer’s instructions. Similarly, DENV pseudovirus was produced by cotransfection of 3 µg of DENV-eGFP-rep and 3 µg of DENV-SP into BHK-21 cells (approximately 1 × 10^6^ cells) using FuGENE HD Transfection Reagent. At 4 h post-transfection (hpt), the transfection medium was replaced with maintenance medium consisting of DMEM supplemented with 2% (v/v) fetal bovine serum and 1% (v/v) penicillin/streptomycin. The cells were subsequently cultured at 37°C with 5% CO_2_. Supernatants containing pseudoviruses were harvested at 48 hpt for VEEV and 96 hpt for DENV. Freshly collected pseudoviruses were aliquoted and stored at -80°C for subsequent detection.

The titers of VEEV and DENV pseudoviruses were determined in BHK-21 cells and are expressed as infectious units per milliliter (IU/mL). Specifically, the pseudovirus-containing supernatants were subjected to 10-fold serial dilutions up to 1:100,000. Afterward, 100 μL of the diluted pseudovirus was inoculated onto BHK-21 cell monolayers in 24-well plates, with two replicates per dilution. The number of eGFP-positive cells from the highest serial dilutions with detectable fluorescence was counted under a fluorescence microscope (EVOS M5000, Invitrogen, CA, USA) at 24 h post infection (hpi). The average number of eGFP-positive cells was multiplied by the corresponding dilution factor to calculate the viral titer.

### Pseudovirus infection assay

To compare the differences in viral infection dynamics between W- and W+ cells, each cell group was infected with either VEEV or DENV pseudoviruses at a multiplicity of infection (MOI) of 0.1. VEEV pseudovirus infection was performed in Aa23T and Aa23 cells at an MOI of 0.1. At 1 hpi, the infected cells were washed three times with phosphate-buffered saline (PBS) to eliminate residual virus particles, and the medium was replaced with fresh cell culture medium. Viral infection from each group was observed via eGFP signals using fluorescence microscopy at 48 hpi. Cells infected with DENV pseudovirus (titer of 1.49 × 10^5^ IU/mL) were harvested at 6, 12, 24, and 48 hpi, with three biological replicates per group per time point for RNA and protein extraction. Cells infected with VEEV pseudovirus (titer of 3.65 × 10^5^ IU/mL) were harvested at 6, 12, and 24 hpi. There were six and three biological replicates per group per time point for RNA and protein extraction, respectively.

To investigate the differential susceptibility of *Aedes* cells to viral infection under different treatments, pretreated cells were infected with VEEV or DENV pseudoviruses at the indicated MOIs, either 48 h post dsRNA/plasmid transfection or 24 h post adenosine/oligomycin treatment. Cell samples were harvested at 12 hpi for VEEV pseudovirus infection and 24 hpi for DENV pseudovirus infection, with six biological replicates per group for RNA extraction.

To explore the genes and proteins that were differentially expressed in response to viral infection, W- cells were infected with either VEEV or DENV pseudoviruses at an MOI of 0.1. The negative control cell group was mock-infected with the corresponding pseudoviruses that had been inactivated by heating at 56°C for 30 min. The cell samples were harvested at designated times, with three replicates for each group for RNA and protein extraction.

### Viral replication assay

To evaluate the differences in viral replication dynamics between W- and W+ cells, each group was transfected with either VEEV-eGFP-rep (5 μg per 10^7^ cells) or DENV-eGFP-rep (5 μg per 10^7^ cells) using the Lipofectamine 2000 reagent (11668019, Invitrogen, CA, USA). The amount of viral replicon was calculated based on the cell number counted immediately prior to the transfection. At 6 hpt, the transfection medium with viral replicons was replaced with fresh cell culture medium. Cells transfected with VEEV-eGFP-rep were harvested at 6, 12, 24, and 48 hpt, with six biological replicates per group per time point, while cells transfected with DENV-eGFP-rep were collected at 12, 24, 48, and 72 hpt, with three biological replicates per group per time point.

To investigate the effects of different treatments on viral replication in *Aedes* cells, 5 µg of VEEV-eGFP-rep or DENV-eGFP-rep was transfected into 10^7^ cells of each group using Lipofectamine 2000 at either 48 h post dsRNA/plasmid transfection or 24 h post-treatment with adenosine/oligomycin. Cell samples were collected at 24 hpt for VEEV-eGFP-rep and 48 hpt for DENV-eGFP-rep, with six biological replicates per group.

### Quantification of mRNA using quantitative PCR

Total RNA was extracted using the FastPure Cell/Tissue Total RNA Isolation Kit V2 (RC112, Vazyme, Nanjing, China) according to the manufacturer’s instructions. RNA concentration and purity were determined using a NanoDrop One spectrophotometer (840–317400, Thermo, MA, USA). After the removal of genomic DNA, 1 µg of total RNA from each sample was reverse transcribed into complementary DNA (cDNA) using a PrimeScript RT Reagent Kit (RR047A, Takara, Kyoto, Japan). A total of 100 ng of cDNA from each sample was subjected to qPCR amplification, which was carried out on a LightCycler 480 Instrument II (LightCycler480, Roche, Basel, Switzerland), using TB Green Premix Ex Taq II (RR820A, Takara, Kyoto, Japan). The thermal cycling protocol consisted of initial denaturation at 95°C for 3 min, followed by 40 cycles of 95°C for 10 seconds and 60°C for 30 seconds. The relative transcript levels of oxidative phosphorylation pathway genes were normalized to the endogenous reference gene, ribosomal protein S6 (RPS6), and calculated using the 2^-ΔΔCt^ method. Additionally, to quantify viral infection and replication levels, the absolute copy numbers of the VEEV *nsP1* or DENV *NS5* genes were determined and normalized to the absolute copy number of the *RPS6 gene* in each sample. The standard curves were generated from tenfold serial dilutions (1 × 10^1^ to 1 × 10^8^ copies) of the corresponding plasmids containing the VEEV *nsP1*, DENV *NS5*, or *RPS6* genes. The primers used for the *RPS6*, DENV *NS5*, and VEEV *nsP1* genes were described previously [84–86]. All remaining primers were synthesized by Qingke Biotechnology Co., Ltd. (Beijing, China), and their sequences are listed in S2 Table.

### Analysis of transcript profile differences by mRNA sequencing

Total RNA was extracted from W- and W+ cells with three biological replicates per group at 12 hpi or 24 hpi following infection with VEEV or DENV pseudoviruses, respectively. RNA extraction was performed using TRIzol Reagent (15596026CN, Invitrogen, CA, USA) according to the manufacturer’s protocol. RNA purity and integrity were determined using a Bioanalyzer 2100 System with an RNA Pico 6000 Assay Kit (Agilent Technologies, CA, USA), and RNA concentration was measured with a Nanodrop2000 Spectrophotometer (Thermo, MA, USA). Subsequent construction of mRNA libraries and mRNA sequencing were performed at Biomarker Technologies (http://www.biomarker.com.cn). Briefly, 1.5 μg of qualified RNA per sample was subjected to ribosomal RNA depletion using the Ribo-Zero rRNA Removal Kit (Epicentre, WI, USA). The mRNA libraries were subsequently synthesized with the NEBNext Ultra Directional RNA Library Prep Kit (New England Biolabs, MA, USA) and purified with AMPure XP Beads (Beckman Coulter, MA, USA). Library quality was evaluated using both an Agilent Bioanalyzer 2100 (Agilent Technologies, CA, USA) and a Qubit 2.0 fluorometer (Thermo, CA, USA). Only qualified libraries were sequenced on the Illumina NovaSeq 6000 platform (Illumina, CA, USA) in paired-end 150 bp (PE150) mode.

Clean reads were obtained from the raw reads in fastq format by removing the adapters, poly-N sequences, and low-quality reads using in-house Perl scripts. Qualified clean reads were aligned to the reference genome of *Ae. aegypti* (GCF_002204515.2) from GenBank (https://www.ncbi.nlm.nih.gov/genbank/) via StringTie (v1.3.1) [87].

Gene expression levels were quantified using the fragments per kilobase of transcript per million fragments mapped (FPKM) method implemented in StringTie [88]. Differentially expressed genes (DEGs) were identified using the DESeq R package (v1.10.1) with the thresholds of an FDR < 0.05 and an absolute value of log2(fold change) ≥1 [89]. On the basis of the gene annotation of the Kyoto Encyclopedia of Genes and Genomes (KEGG) database (https://www.genome.jp/kegg/), the top 5 activated pathways were determined according to the proportion of upregulated DEGs in each pathway, and the results were visualized as a heatmap using GraphPad Prism software.

### Immunofluorescence assay

Cells seeded on chamber slides (Biologix, Shanghai, China) were fixed with 4% paraformaldehyde at 4 °C for 1 h, and then permeabilized with 0.1% Triton X-100 (CT1451, Coolaber, Beijing, China) for 15 min, followed by blocking with 5% BSA in PBS buffer containing 0.1% Tween-20 (PBS-T) for 2 h. Subsequently, the cells were incubated with a rabbit monoclonal anti-ATPSβ1 primary antibody (17247–1-AP, Proteintech, Wuhan, China) at a 1:500 dilution for 2 h, followed by incubation with DyLight 594-conjugated goat anti-rabbit IgG secondary antibody (A23420, Abbkine, Wuhan, China) at a 1:2,000 dilution for 1 h in the dark. After washing three times with PBS-T, the samples were stained with 1 μg/mL DAPI (Beyotime, Shanghai, China) for 5 min in the dark. Fluorescence signals were visualized using a Leica confocal microscope system (TCS SP8, Leica, Wetzlar, Germany). 14 individual images per experimental group were randomly captured under constant acquisition parameters, including laser intensity, power, master gain, offset, and digital gain.

### Binding relationship prediction

The three-dimensional structures of *Ae. aegypti ATPSβ1*, VEEV E2, and DENV E protein were predicted using AlphaFold3 (https://alphafoldserver.com/) with default parameters [49], based on amino acid sequences published in the National Center for Biotechnology Information (NCBI, https://www.ncbi.nlm.nih.gov/) and listed in S3 Table. Using AlphaFold3-derived structures with the highest predicted Template Modeling score (pTM) as receptor and ligand inputs, the binding relationships between ATPSβ1 and the VEEV or DENV E proteins were predicted using the HDOCK server (http://hdock.phys.hust.edu.cn/) in global docking mode [50]. The optimal complex structural models with top-ranked Docking Score, Confidence Score, and binding energy were visualized using PyMOL (version 3.1.0) [90]. Amino acid sequence alignment of the predicted ATPSβ1-binding residues in DENV envelope (E) and VEEV E2 proteins was conducted using protein sequences from representative strains of six genotypes within DENV serotype 2 (Asian I, Asian II, Cosmopolitan, American, Asian/American, and Sylvatic) as well as epidemic VEEV subtypes IAB and IC.

### Viral binding assay

Pseudovirus was added to each cell well at an MOI of 0.01 for DENV or an MOI of 0.1 for VEEV, then cells were incubated at 4°C for 1 hour to allow viral binding to the cell surface. At 1 hour post inoculation, supernatants containing unbound pseudovirus and cells with bound pseudovirus were collected separately from each well for quantification of unbound and bound viruses. The cells were washed three times with precooled PBS to remove residual unbound viral particles and then harvested in 200 µL of lysis buffer (Vazyme, Nanjing, China) per well. Bound pseudovirus was quantified by qPCR as described in the section on Quantification of mRNA using quantitative PCR. In parallel, supernatants containing unbound pseudovirus were serially diluted from 1:10–1:100,000. Aliquots of 100 μL of each dilution were inoculated onto BHK-21 cell monolayers in 24-well plates, with two replicates per dilution. At 24 h post-infection (hpi), the titration of unbound virus was determined by counting eGFP-positive cells in wells corresponding to the highest serial dilution that showed detectable fluorescence under a fluorescence microscope (EVOS M5000, Invitrogen, CA, USA). The viral titer was calculated as described in the section on Pseudovirus production and titration.

### Western blotting

Total protein was extracted from each cell group using the whole protein extraction reagent (KeyGEN, Jiangsu, China). The extracted proteins were subsequently quantified via a BCA protein assay kit (KeyGEN, Jiangsu, China) following the manufacturer’s instructions, as previously described [25]. Following incubation at 100°C for 10 min with protein loading buffer (Coolaber, Beijing, China), 8 µg of denatured protein per sample was subjected to 10% sodium dodecyl sulfate‒polyacrylamide (SDS‒PAGE) gel electrophoresis, initially at 80 V until the samples entered the separation gel and then at 120 V until electrophoresis was completed. After electrophoresis, the proteins were transferred onto polyvinylidene difluoride (PVDF) membranes (Millipore, MA, USA) at a constant current of 280 mA. The membranes were blocked with 5% nonfat milk for 1 h at room temperature and then incubated overnight at 4°C with primary antibodies, including a rabbit monoclonal anti-ATPSβ antibody (17247–1-AP, Proteintech, Wuhan, China; dilution 1:2000) and a rabbit monoclonal anti-β-actin antibody (20536–1-AP, Proteintech, Wuhan, China; dilution 1:2000). Following three 10-min washes with TBS-T (0.1% Tween-20) at room temperature, the membranes were incubated with a horseradish peroxidase (HRP)-labeled goat anti-rabbit IgG secondary antibody (A21020, Abbkine, Wuhan, China; dilution 1:5000) for 2 h at room temperature. The protein bands were visualized using an enhanced chemiluminescence (ECL) staining solution (BMU101, Abbkine, Wuhan, China) on a ChemiDoc MP Imaging System (Bio-Rad). The relative expression level of ATPSβ1 protein was quantified by analyzing the band gray values with ImageJ software, with the intensity of ATPSβ1 normalized to that of an internal reference protein, β-actin, in the same sample.

### Fluorescence in situ hybridization (FISH)

Cells from each treatment group were fixed on chamber slides (Biologix, Shanghai, China) using 4% paraformaldehyde (Biosharp, Beijing, China) at 4°C for 1 h. Subsequently, cell permeabilization and blocking were performed according to the manufacturer’s instructions provided with the RNA FISH Kit (GenePharma, Shanghai, China), as described previously [25]. Briefly, hybridization was carried out using a *Wolbachia* 16S rDNA probe (1 µM) labeled with Sulfo-Cyanine3 (Cy3) (GenePharma, Shanghai, China) in Buffer E at 37°C for 24 h in the dark. After being washed, the cell nuclei were stained with 1 µg/mL DAPI (Beyotime, Shanghai, China) at room temperature for 15 min in the dark. The cells were subsequently imaged on a Leica confocal microscope system (TCS SP8, Leica, Wetzlar, Germany). Through Leica Application Suite software, 5–15 images were randomly captured for each sample across different groups. All the images were acquired with identical imaging parameters, including laser intensity, laser power, master gain, digital offset, and digital gain values. The *Wolbachia* fluorescence intensity was quantified using ImageJ software and normalized to the number of cells.

### Detection of *Wolbachia* genomic DNA by qPCR

At the indicated time points, cells from each group were digested with Trypsin-EDTA solution (Biosharp, Beijing, China) and collected in 200 µL of cell culture medium. Subsequently, genomic DNA (gDNA) was extracted using the FastPure Blood/Cell/Tissue/Bacteria DNA Isolation Mini Kit (Vazyme, Nanjing, China) following the manufacturer’s instructions. A total of 100 ng of gDNA was used for qPCR performed on a LightCycler 480 II (Roche, Basel, Switzerland). Amplification was carried out using primers specific to the *Wolbachia* surface protein (*WSP*) gene and the *Aedes RPS6* gene, which served as an internal reference gene for both *Ae. aegypti* and *Ae. albopictus*, as described previously [84,91]. The qPCR program consisted of an initial denaturation step at 95°C for 3 min, followed by 40 cycles of denaturation at 95°C for 10 seconds, annealing at 60°C for 30 seconds, and extension at 72°C for 10 seconds. In *Ae. aegypti*, *Wolbachia* density was quantified by the absolute copy number of the *WSP* gene normalized to that of the *RPS6* gene. Standard curves were generated from tenfold serial dilutions (ranging from 1 × 10¹ to 1 × 10^-8^ ng/µL) of plasmids harboring the target *WSP* or *RPS6* gene sequences. In *Ae. albopictus*, the change in *Wolbachia* density was quantified by the relative expression levels of the *WSP* gene normalized to those of the *RPS6* gene and calculated using the 2^-ΔΔCt^ method.

### *ATPSβ1* overexpression assay

Cells were seeded into 96-well (W + : 1 × 10^5^ cells per well; W-: 8 × 10^4^ cells per well) or 6-well (W + : 1 × 10^6^ cells per well; W-: 8 × 10^5^ cells per well) plates. When reached approximately 80% confluence, cells were transfected with recombinant plasmid pIB/V5-ATPSβ1 (for ATPSβ1 overexpression) or pIB/V5-GFP (as a negative control) at a final concentration of 2 µg/mL using Lipofectamine 2000 (Invitrogen, CA, USA). The overexpression efficiency of *ATPSβ1* was evaluated via qPCR at 24 hpt and 48 hpt, with three biological replicates per group.

### RNA interference assay

Double-stranded RNA (dsRNA) was synthesized using the MEGAscript T7 Transcription Kit (AM1334, Invitrogen, CA, USA). Subsequent purification of the synthesized dsRNA was performed with the MEGAclear Kit (AM1908, Invitrogen, CA, USA), strictly following the manufacturer’s protocols, as previously described [25]. All primers used for dsRNA synthesis were designed to incorporate the T7 promoter sequence (5’-TAATACGACTCACTATAGGG-3’), and the detailed primer sequences are listed in S4 Table. To downregulate *ATPSβ1* gene expression, the cells were transfected with ATPSβ1-targeting dsRNA at a final concentration of 4 µg/mL using Lipofectamine 2000 (Invitrogen, CA, USA). The dsRNA of *GFP* was used as the negative control for *ATPSβ1* downregulation assay alone, and for viral binding assay performed after *ATPSβ1* silencing. While the dsRNA of *RLUC* served as the negative control for viral infection and replication assays conducted following *ATPSβ1* knockdown. Gene knockdown efficiency was evaluated at 24 hpt and 48 hpt. Each experimental group was established with three biological replicates.

### Detection of ATP synthase activity

ATP synthase (mitochondrial Complex V) activity was determined using a CheKine Mitochondrial Complex V Activity Assay Kit (KTB1890, Abbkine, Wuhan, China) according to the manufacturer’s instructions. Briefly, 5 × 10⁶ cells per sample were harvested in 1010 µL of freshly prepared collection buffer provided in the kit. Afterward, cell lysis was performed on ice using an ultrasonic cell crusher (SCIENTZ-IID, Ningbo, China) set at 200 W, with 30 cycles of 3-second sonication followed by 7-second intervals for cooling. The cell lysate was centrifuged and resuspended. A 50 µL aliquot of the supernatant was transferred to a 96-well white plate (PT06-96S, BIOLAND, Zhejiang, China). Following the addition of the assay substrate mix provided in the kit to each well, the absorbance of the samples at 660 nm was recorded using a multimode microplate reader (Synergy 2, BioTek, VT, USA). ATP synthase activity was quantified against a standard curve generated using serially diluted ATP standards with concentrations of 0.25 mM, 0.125 mM, 0.0625 mM, 0.03125 mM, and 0.015625 mM. All activity values were normalized to the initial cell count (5 × 10⁶ cells per sample). The relative ATP synthase activity is presented as the fold change in activity in the treatment group compared with that in the control group.

### Measurement of intracellular ATP levels

Intracellular ATP levels were quantified using an ATP Assay Kit (S0026, Beyotime, Shanghai, China) following the manufacturer’s standard protocol. For each experimental group, one sample was randomly selected for cell counting to determine the initial cell number per sample. The remaining samples were processed for ATP extraction by lysis in 50 µL of ice-cold lysis buffer. After centrifugation at 4°C, a 40 µL aliquot of the supernatant was transferred to a 96-well black plate (J09605, JingAn Biological, Shanghai, China). Following the addition of 50 µL of working-concentration ATP assay solution freshly prepared from the kit stock solution in the dark, the fluorescence intensity was immediately measured on a multimode microplate reader (Synergy 2, BioTek, VT, USA). The intracellular ATP concentration was determined by interpolating the sample fluorescence intensity values against a standard curve generated from serially diluted ATP standards (ranging from 10 nM to 2000 nM). All values were normalized to the corresponding cell counts, and relative ATP levels are expressed as fold changes compared with those in the control group.

### Adenosine and oligomycin treatments

Adenosine (CA1241, Coolaber, Beijing, China) was dissolved in dimethyl sulfoxide (DMSO; D8371, Solarbio, Beijing, China) as the vehicle. For adenosine treatment, 1 µL of adenosine solution was added to each well of a 96-well cell culture plate containing 100 µL of culture medium, resulting in final concentrations of 0.1 µM, 1 µM, and 10 µM. A vehicle control group was established by adding 1 µL of DMSO to wells with 100 µL of complete medium.

Oligomycin (HY-N6782, MCE, Shanghai, China) was similarly prepared in DMSO (Solarbio, Beijing, China). For oligomycin treatment, cells in 96-well plates containing 100 µL of culture medium were treated with 1 µL of oligomycin solution to achieve final concentrations of 0.2 µg/mL, 2 µg/mL, and 20 µg/mL, with a corresponding DMSO control group included.

### Assessment of cellular viability

At 48 hpt with plasmids or dsRNA to regulate ATPSβ1 expression, cell viability was evaluated using the cell counting kit-8 (CCK-8) assay (C0039, Beyotime, Shanghai, China) according to the manufacturer’s instructions. Briefly, 10 μL of CCK-8 buffer was added to each cell well of 96-well plate and then incubated at 26 °C for 2 h. Subsequently, the absorbance at 450 nm of each cell well was measured using a multimode microplate reader (Synergy 2, BioTek, VT, USA). Background absorbance from a blank well containing only cell culture medium and CCK-8 buffer was subtracted from all experimental readings. Cell viability in each treatment group was calculated relative to that of the control group (100% viability), with eight biological replicates per group.

### Statistical analysis

All the statistical analyses were performed using IBM SPSS Statistics 25 software (IBM Corp., Armonk, NY, USA). The normality of the data distribution and homogeneity of variances were assessed prior to analysis to ensure the appropriateness of the statistical methods. Repeated-measures ANOVA was used to assess differences in viral infection and replication levels between W- and W+ cells across multiple time points. One-way ANOVA was used to compare *Wolbachia* density on day 1, day 2, and day 3 post-seeding, the levels of bound and unbound virus in dsRNA/plasmid-transfected W- and W+ cells, and the effects of different concentrations of adenosine or oligomycin on intracellular ATP levels. Unpaired two-tailed t tests were used to evaluate differences between the control group and experimental group in terms of mRNA expression, protein levels, intracellular ATP synthase activity, intracellular ATP levels, *Wolbachia* density, *Wolbachia* fluorescence intensity, levels of bound and unbound virus, and viral infection/replication levels. A p value of less than 0.05 was considered to indicate statistical significance.

## Supporting information

S1 Fig The infection of VEEV or DENV pseudovirus in different cell types. Green fluorescence corresponds to the eGFP signal from the DENV or VEEV pseudovirus, which carries an eGFP reporter. The scale bar represents 300 μm. W-(DENV-) refers to W- cells infected with heat-inactivated DENV pseudovirus (negative control); W-(DENV+) and W+(DENV+) refer to W- and W+ cells infected with infectious DENV pseudovirus, respectively; BHK-21 (VEEV-) and BHK-21 (VEEV+) refer to BHK-21 cells infected with heat-inactivated and infectious VEEV pseudovirus, respectively; BHK-21 (DENV-) and BHK-21 (DENV+) refer to BHK-21 cells infected with inactivated and infectious DENV pseudovirus, respectively.
(TIF)

S2 Fig The *ATPSβ1* expression in W + / W- cells at 48 h post-transfection (hpt). (**A**) The *ATPSβ1* expression levels in pIB/V5-ATPSβ1 versus pIB/V5-GFP groups in W+ cells at 48 hpt (two-sided t-test, n = 3, fold change = 2.16, *P* = 0.005964). (**B**) The changes of *ATPSβ1* expression levels in W+ cells at 48 hours post dsATPSβ1 transfection (two-sided t test, n = 3, Knockdown efficiency = 86.41%, *P* = 0.000133). (**C**) The *ATPSβ1* expression levels between pIB/V5-ATPSβ1 group and pIB/V5-GFP group in W- cells (two-sided t-test, n = 3, fold change = 2.18, *P* = 0.004999). (**D**) Knockdown efficiency of *ATPSβ1* in W- cells (two-sided t test, n = 3, Knockdown efficiency = 90.37%, *P* = 0.000019). dsRLUC: control group. The error bars indicate the standard error of the mean (SEM). ****P < 0.0001, ***P < 0.001 and **P < 0.01.
(TIF)

S3 Fig Cell viability in W+ and W- cells following ATPSβ1 expression regulation. (**A**) Cell viability in W+ cells post ATPSβ1 overexpression at 48 hpt (two-sided t test, n = 8, *P* = 0.552598). (**B**) Cell viability in W+ cells post ATPSβ1 knockdown at 48 hpt (two-sided t test, n = 8, *P* = 0.400464). (**C**) Cell viability in W+ cells post ATPSβ1 overexpression at 48 hpt (two-sided t test, n = 8, *P* = 0.320068). (**D**) Cell viability in W+ cells post ATPSβ1 knockdown at 48 hpt (two-sided t test, n = 8, *P* = 0.170012). The error bars indicate the standard error of the mean (SEM), and ns indicates nonsignificance.
(TIF)

S4 Fig Representative fluorescence images of ATPSβ1 localization in W- and W+ cells. The red fluorescence indicates ATPSβ1 protein, blue fluorescence (DAPI) marks nuclei, and the scale bar represents 10 μm.
(TIF)

S5 Fig Amino acid sequence alignment of the predicted ATPSβ1-binding residues in VEEV E2 and DENV envelope (E) proteins across representative viral strains. (A) Alignment of predicted ATPSβ1-binding residues in VEEV E2 among epidemic strains of subtypes IAB (blue) and IC (green). The β-ribbon connector and Domain C regions are annotated above the alignment. For each strain, the isolate designation and the corresponding NCBI accession number are listed on the left. (B) Alignment of predicted ATPSβ1-binding residues in DENV E protein among six representative DENV-2 genotypes (Asian I, Asian II, Cosmopolitan, American, Asian/American, and Sylvatic). The Ectodomain II and Stem regions are annotated above the alignment. Amino acid positions are numbered below each block. Black triangles (▲) mark the positions of the predicted ATPSβ1-binding residues.
(TIF)

S6 Fig Adenosine increases intracellular ATP levels and enhances VEEV and DENV infection in W- cells. (**A**) Experimental schematic for intracellular ATP detection following adenosine treatment in W- cells. (**B**) Intracellular ATP levels in W- cells at 12 h post-treatment (two-sided t test, n = 8 per group, *P* = 6.038300 × 10^-9^). (**C**) Experimental schematic for VEEV pseudovirus infection assay after adenosine treatment in W- cells. (**D**) VEEV infection levels in in W− cells treated with adenosine or DMSO (NC) at 12 hpi (two-sided t-test, n = 6 per group, *P* = 0.000140). (**E**) Experimental schematic for DENV pseudovirus infection assay after adenosine treatment in W- cells. (**F**) DENV infection levels in W− cells treated with adenosine or DMSO (NC) at 24 hpi (two-sided t test, n = 6 per group, *P* = 0.000042). Error bars indicate the standard error of the mean (SEM). ****P < 0.0001 and ***P < 0.001.
(TIF)

S7 Fig Oligomycin reduces *Wolbachia* density and enhances VEEV and DENV pseudovirus infection in W+ cells. (**A**) Experimental schematic for Wolbachia density measurement following oligomycin treatment in W+ cells. (**B**) *Wolbachia* density in W+ cells at 24 h post-oligomycin treatment (two-sided t test, n = 6 per group, *P* = 0.000031). (**C**) Experimental schematic for VEEV pseudovirus infection assay after oligomycin treatment in W+ cells. (**D**) VEEV infection levels in W+ cells treated with oligomycin or DMSO negative control at 12 hpi. (two-sided t test, n = 6 per group, *P* = 0.038374). (**E**) Experimental schematic for DENV pseudovirus infection assay after oligomycin treatment in W+ cells. (**F**) DENV infection levels in W+ cells treated with oligomycin or negative control at 24 hpi (two-sided t test, n = 6 per group, *P* = 0.008005). Error bars indicate the standard error of the mean (SEM). ****P < 0.0001, **P < 0.01 and *P < 0.05.
(TIF)

S8 Fig Comparison of VEEV infection levels between W- and W+ cells at 12 hpi. Comparison of VEEV infection levels between Aa23 and Aa23T cells (two-sided t test, n = 6 per group, *P* = 1.410300 × 10^-10^). Aa23 cells are *Ae. albopictus* cells stably infected with *Wolbachia*, whereas Aa23T cells are the corresponding *Wolbachia*-cured control cell line. Error bars indicate the standard error of the mean (SEM). ****P < 0.0001.
(TIF)

S1 Table *Wolbachia*-induced *Ae. aegypti* transcriptional alterations during VEEV (12 hpi) or DENV (24 hpi) pseudovirus infection. (XLSX)

S2 Table The primer information used in quantitative PCR. Note: *ATPSβ1*: ATP synthase beta subunit 1; *V-ATPase D1*: V-type proton ATPase subunit d 1; *Ndufa9*: NADH dehydrogenase 1 alpha subcomplex subunit 9; *Ndufs1*: NADH-ubiquinone oxidoreductase 75 kDa subunit.
(DOCX)

S3 Table The sequences used for the prediction of complexes by AlphaFold3. (DOCX)

S4 Table The primers used for double-stranded RNA synthesis. Note: The T7 promoter sequence TAATACGACTCACTATAGGG was added to the 5’ end of the above sequence for primer synthesis.
(DOCX)

S1 Raw Gel Raw images in this study. (PDF)

S1 Data Supporting data underlying the figures. (XLSX)
