# Supplementary material for: Wolbachia outcompetes mosquito-borne viruses by reprogramming host ATPSβ1-driven ATP metabolism toward symbiotic proliferation
Source: PLoS Pathog. 2026 Oct 21;22(10):e1014692. doi: 10.1371/journal.ppat.1014692 (PMC13712716; doi:10.1371/journal.ppat.1014692)
Supplement: Supplementary file 1 [file ppat.1014692.s013.pdf]

Fig 1A

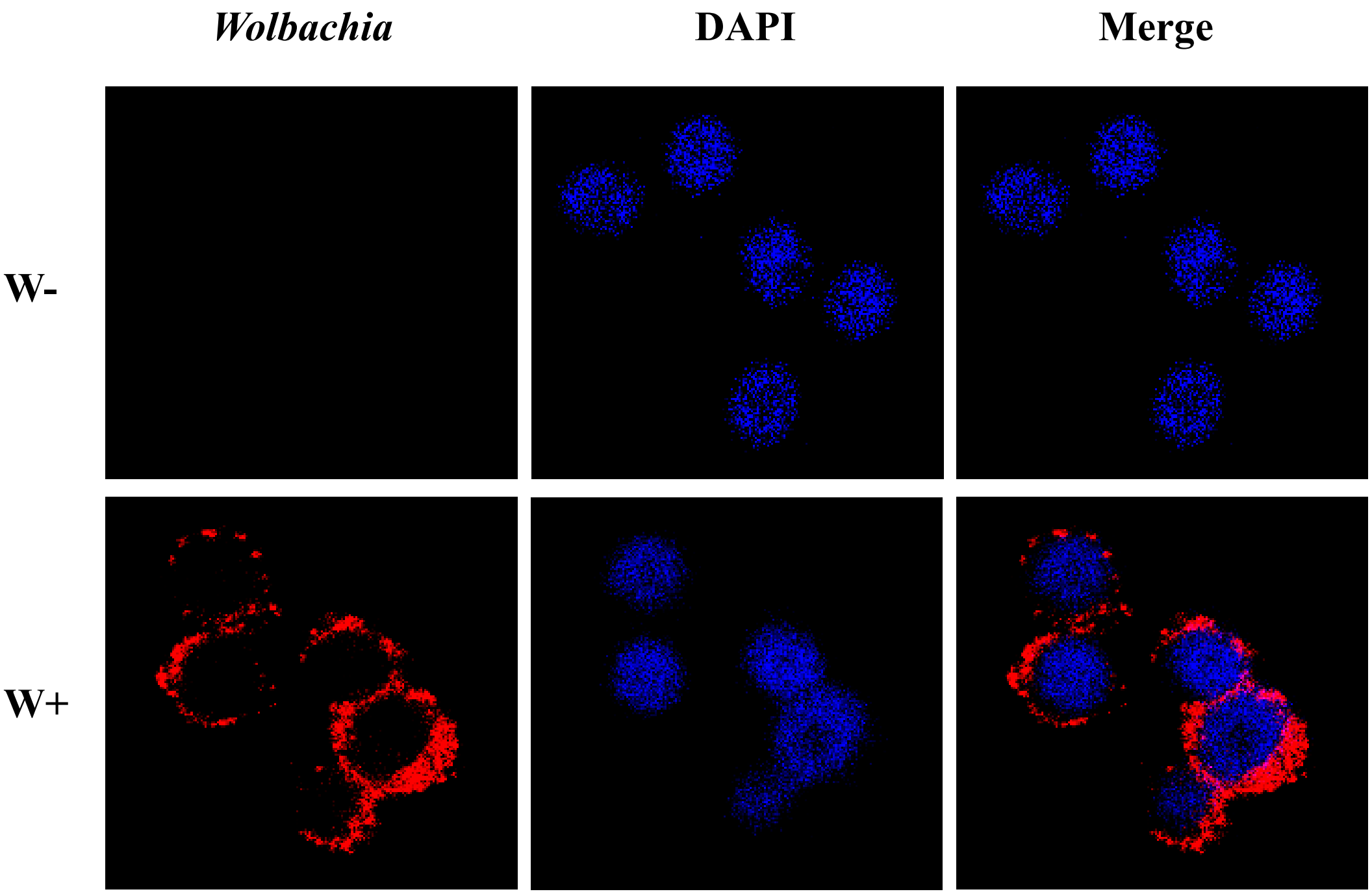

Note: W- means *Wolbachia* free *Ae. aegypti* cells, and W+ means *Wolbachia* *w*AlbB infected *Ae. aegypti* cells.

Fig 2E

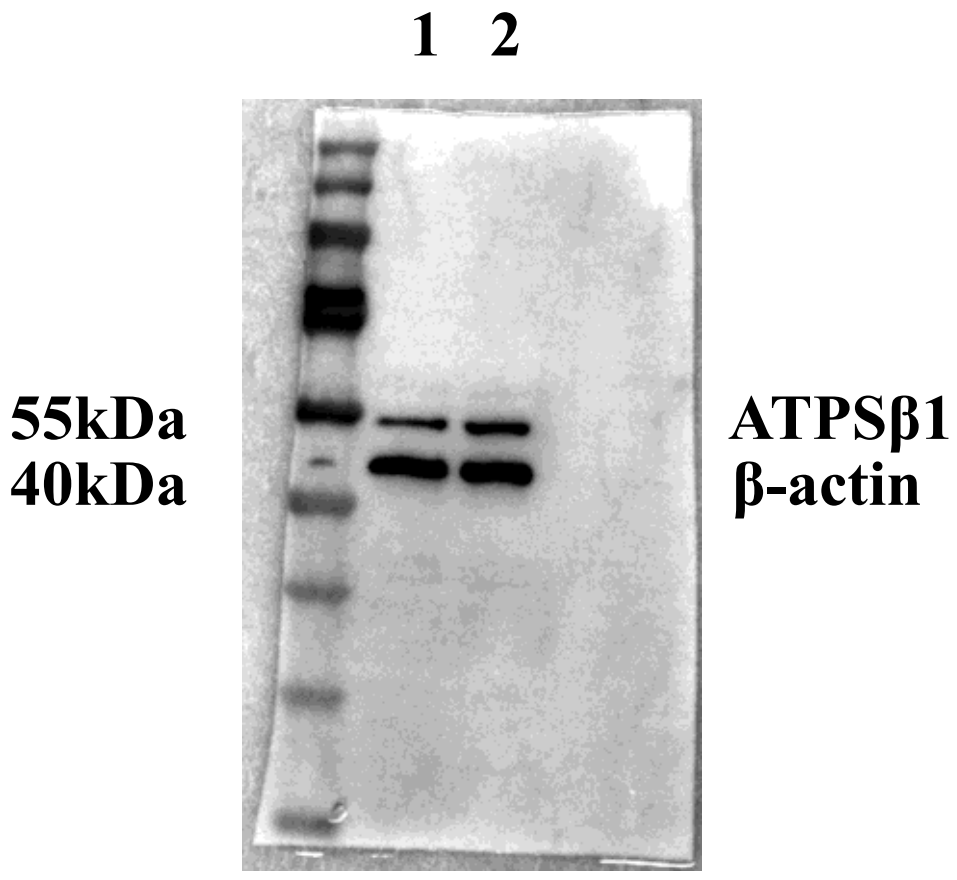

Note: Representative western blot image of ATPSβ1 protein in VEEV infection W- and W+ cells at 24 hpi. Lanes 1 and 2 of the above images represent the W-(VEEV+) and W+(VEEV+) groups, respectively (From left to right are marker, lanes 1 and 2). W-(VEEV+): VEEV-infected W- cells, W+(VEEV+): VEEV-infected W+ cells.

Fig 2F

S1

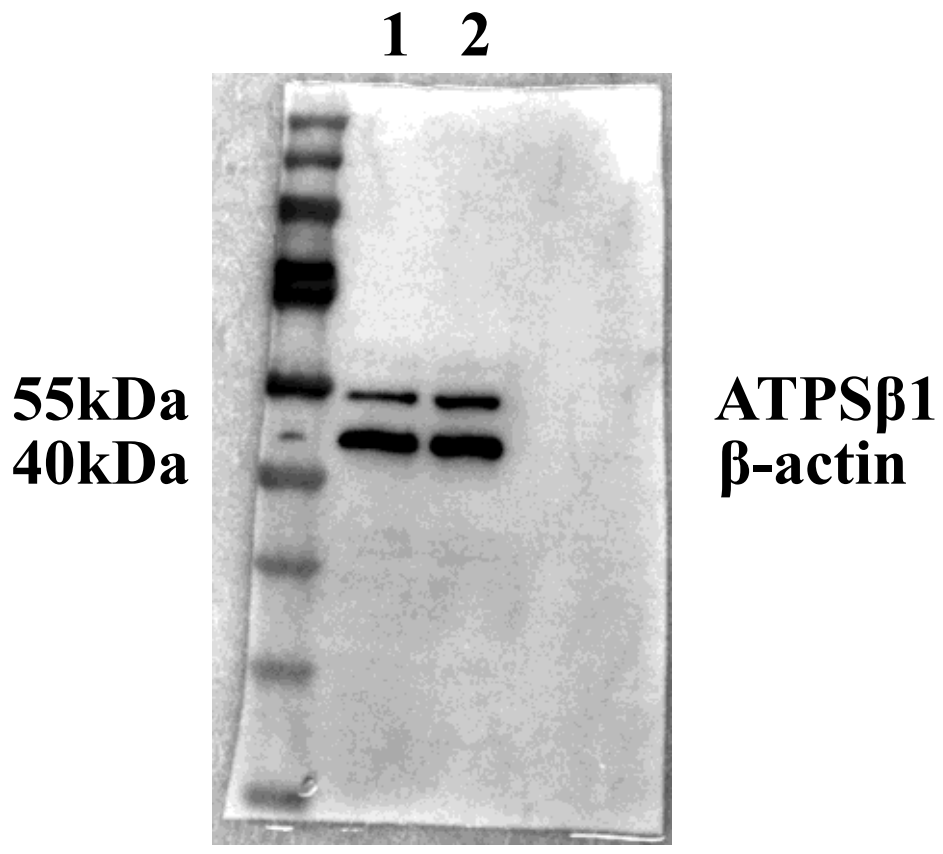

S2

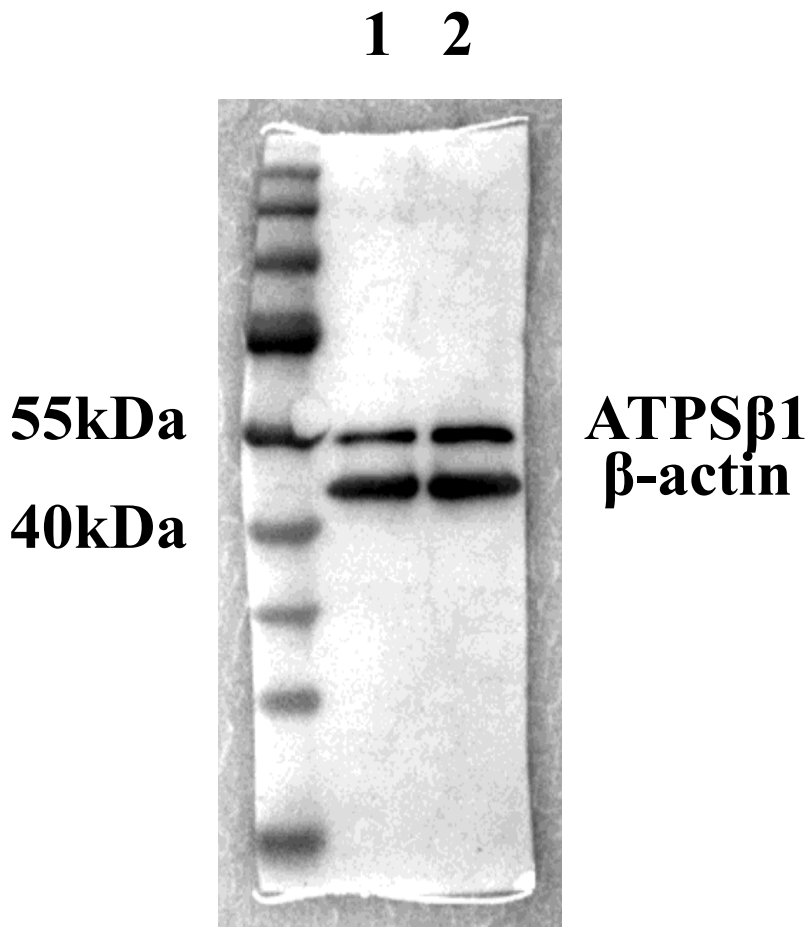

S3

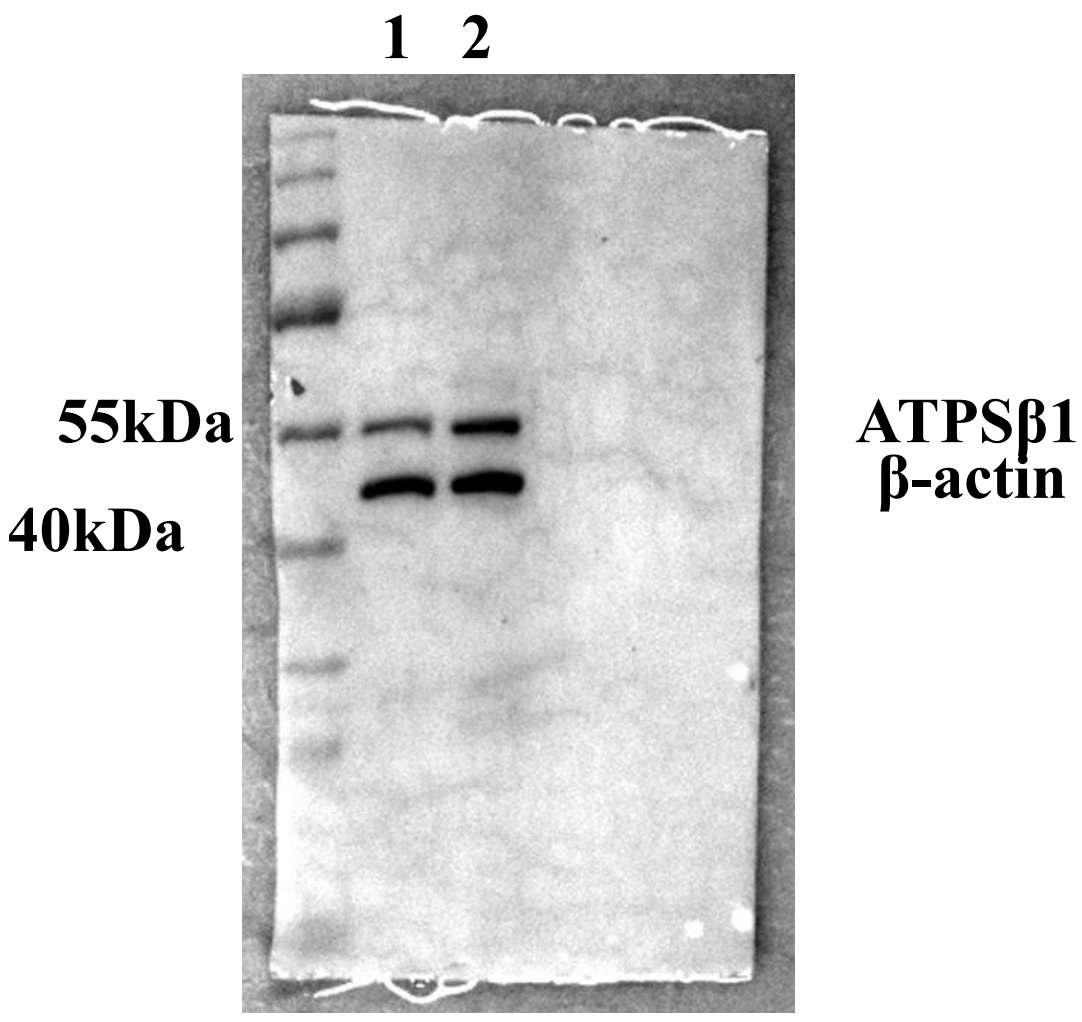

Note: Lanes 1 and 2 of the above images represent the W-(VEEV+) and W+(VEEV+) groups, respectively (From left to right are marker, lanes 1 and 2).

Fig 2G

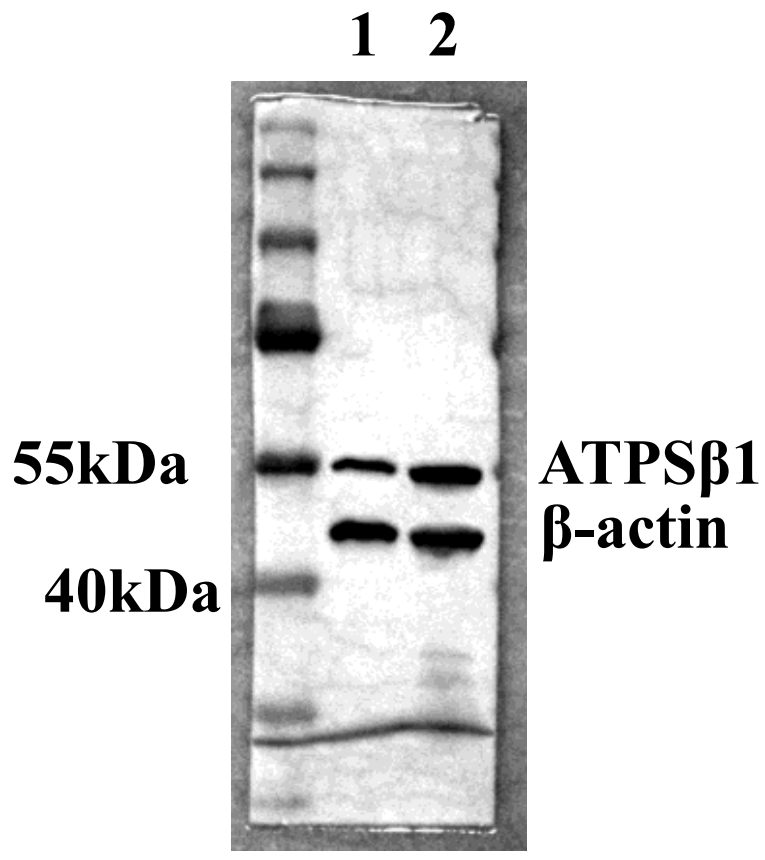

Note: Representative western blot image showing ATPSβ1 protein in DENV infection W- and W+ cells at 48 hpi. Lanes 1 and 2 of the above images represent the W-(DENV+) and W+(DENV+) groups, respectively (From left to right are marker, 1 and 2 lanes). W-(DENV+): DENV-infected W- cells, W+(DENV+): DENV-infected W+ cells.

Fig 2H

S1

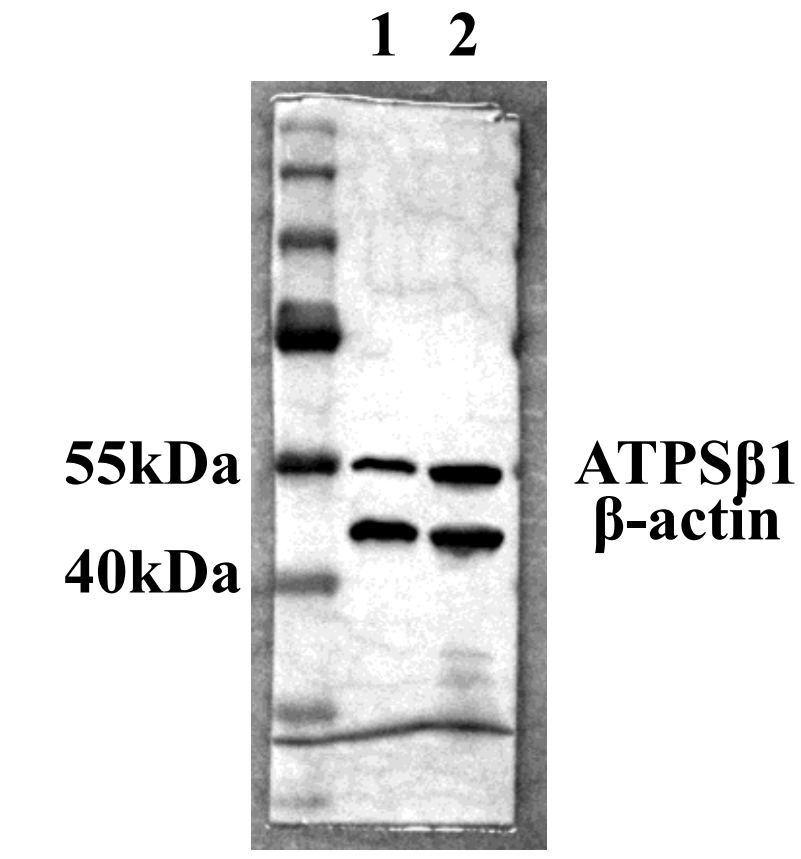

S2

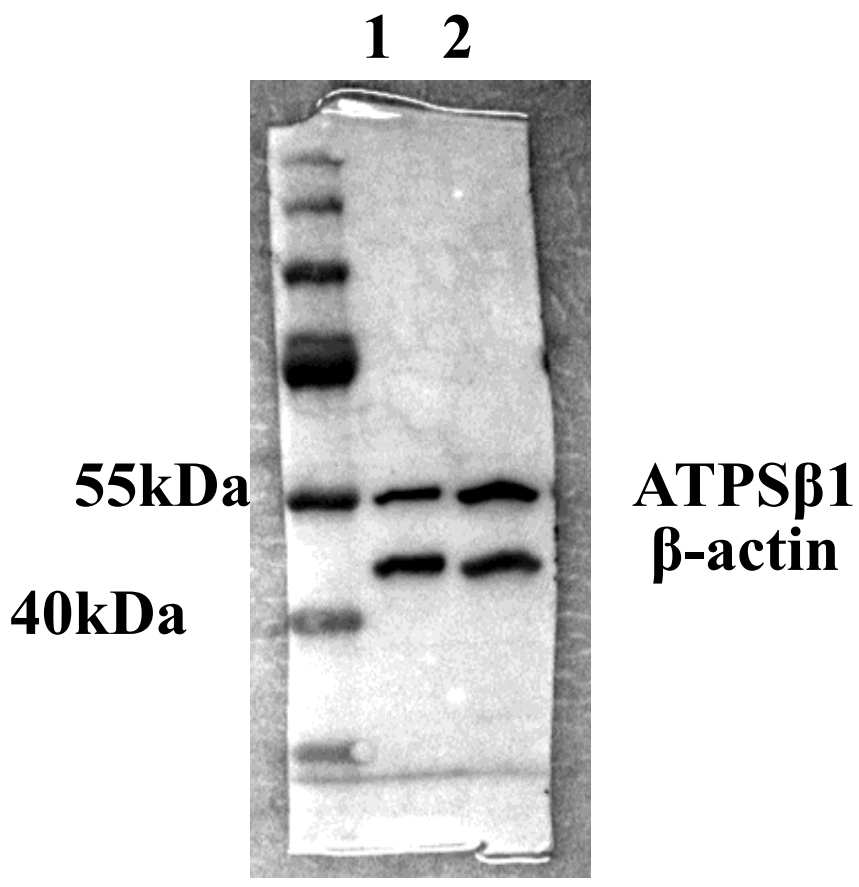

S3

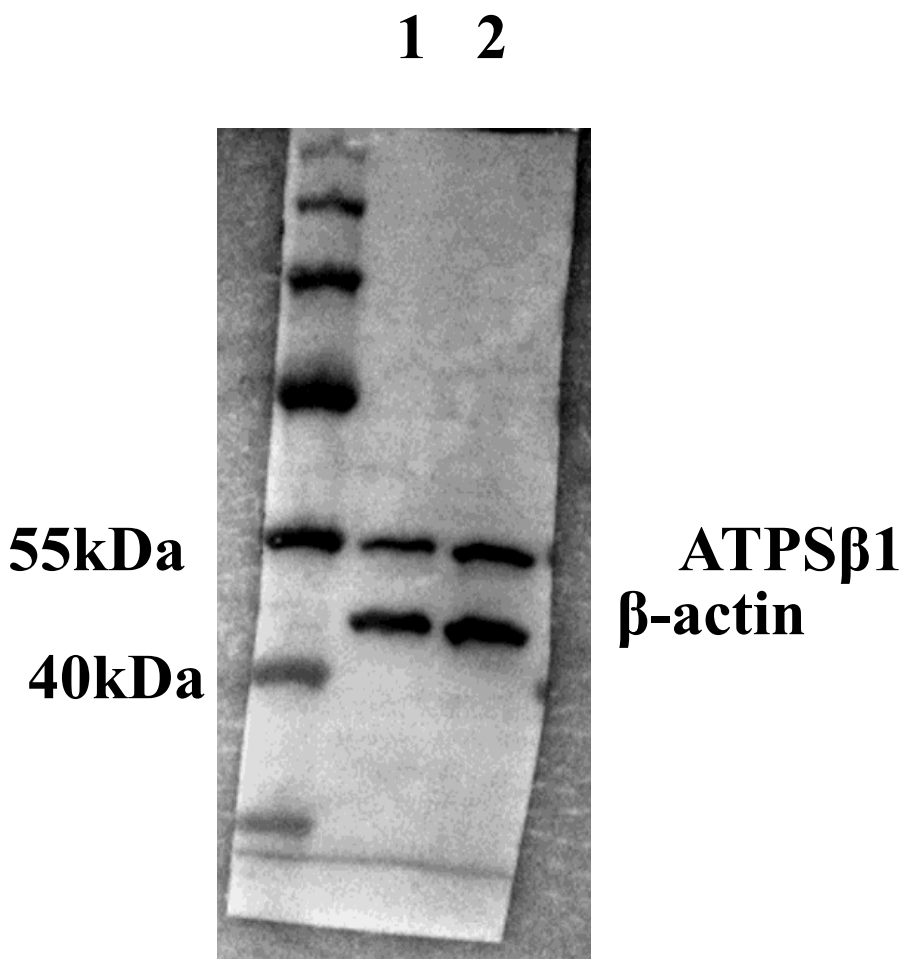

Note: Lanes 1 and 2 of the above images represent the W-(DENV+) and W+(DENV+) groups, respectively (From left to right are marker, 1, and 2 lanes).

Fig 3B

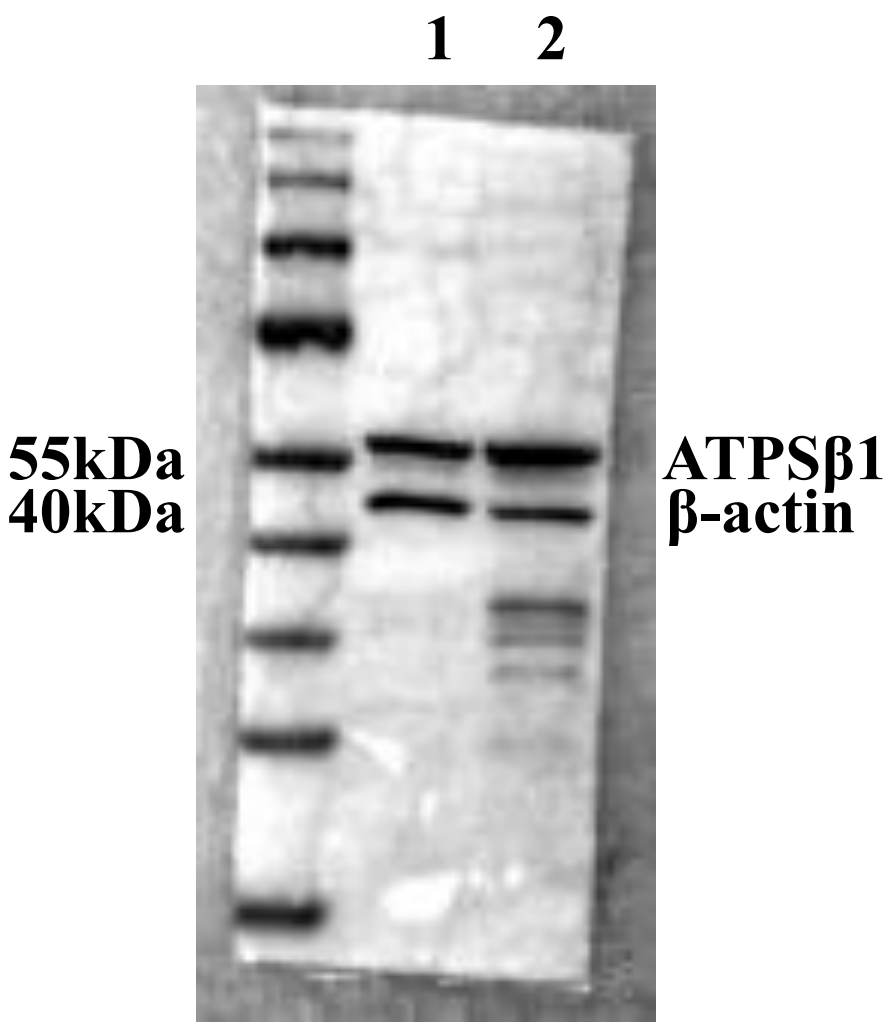

Note: Representative western blot of ATPSβ1 protein in W+ cells transfected with pIB/V5-GFP (control) or pIB/V5-ATPSβ1 at 48hpt. Lanes 1 and 2 of the above images represent the pIB/V5-GFP and pIB/V5-ATPSβ1 groups, respectively (From left to right are marker, lanes 1 and 2).

Fig 3C

S1

1 2

55kDa ATPSβ1
40kDa β-actin

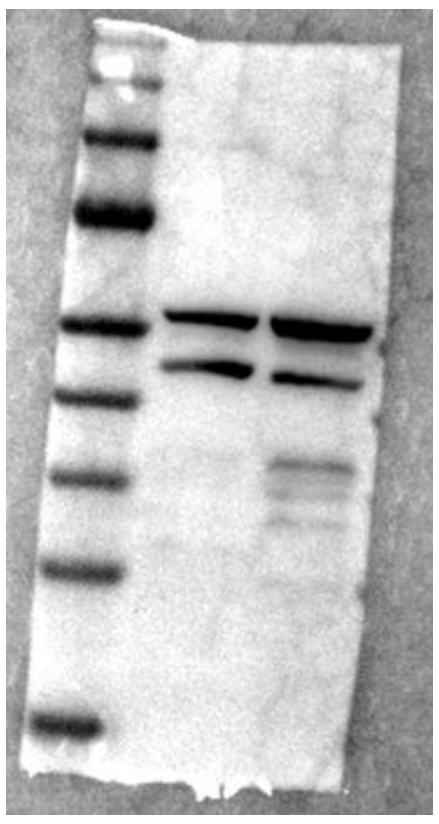

S2

1 2

55kDa ATPSβ1
40kDa β-actin

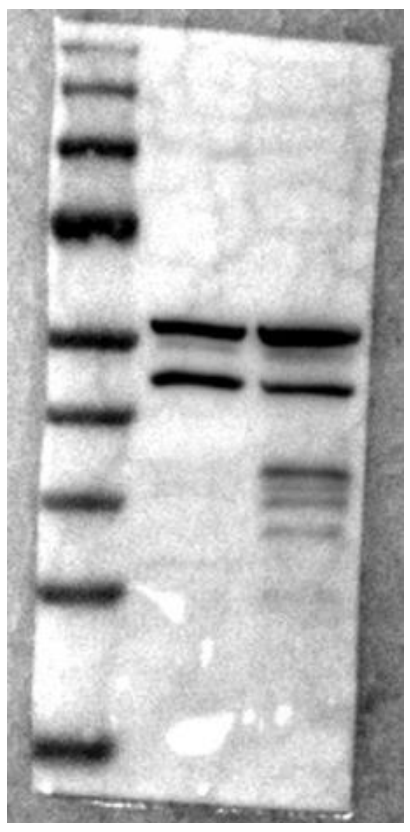

S3

1 2

55kDa ATPSβ1
40kDa β-actin

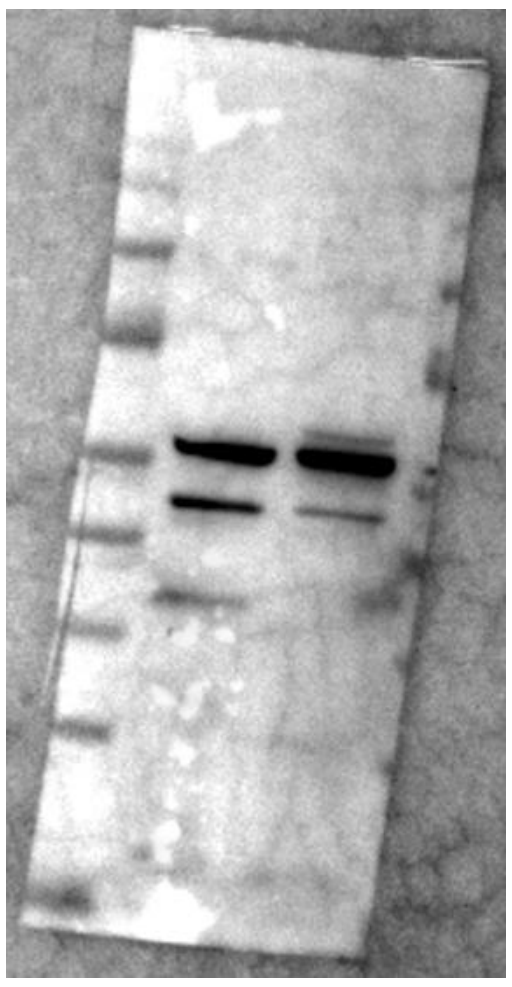

Note: Lanes 1 and 2 of the above images represent the pIB/V5-GFP and pIB/V5-ATPSβ1 groups in W+ cell, respectively (From left to right are marker, lanes 1 and 2).

Fig 3F

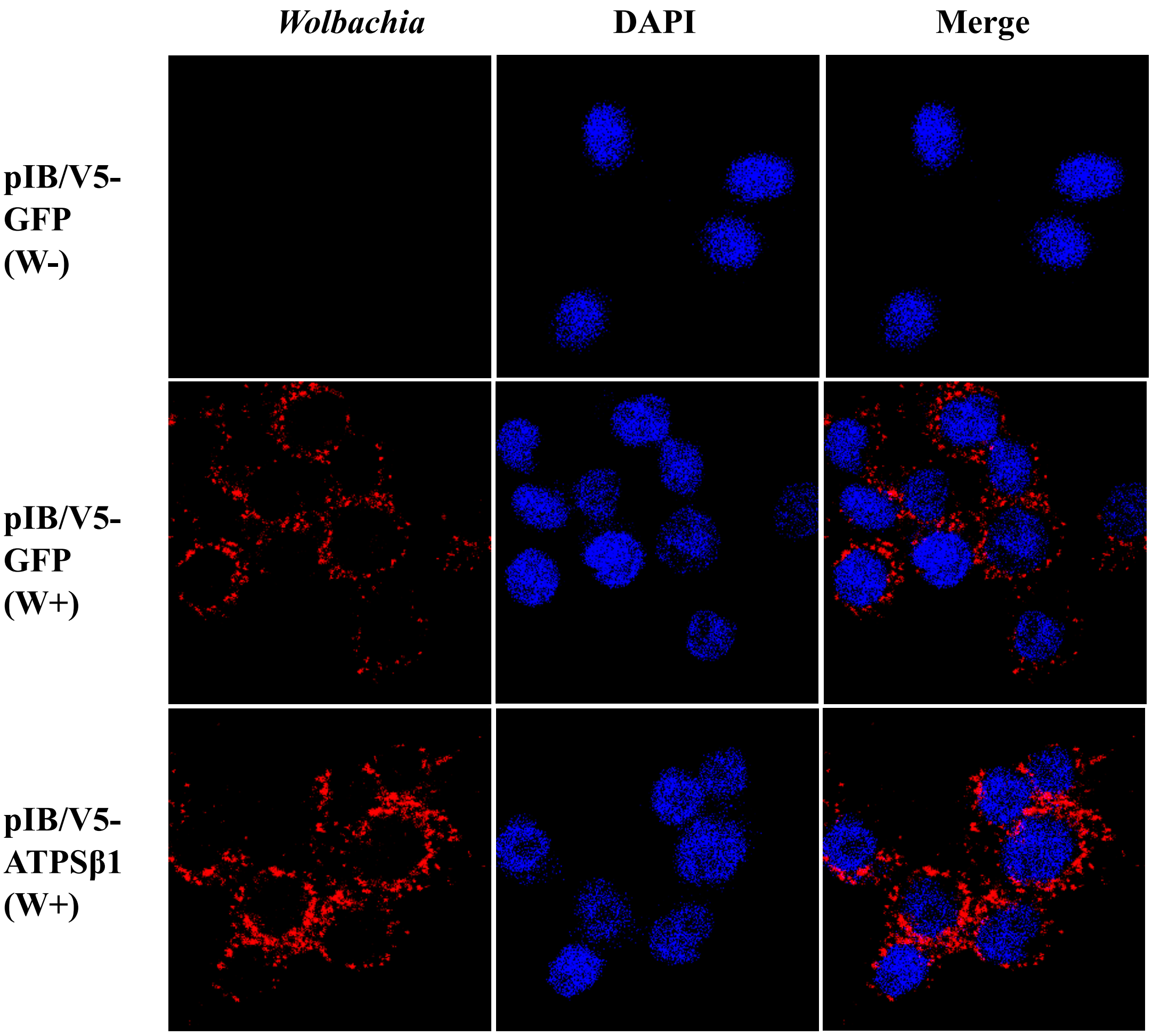

Note: pIB/V5-GFP(W-) means *GFP* plasmid-transfected W- cells as a negative control for the *Wolbachia* probe, pIB/V5-GFP(W+) means *GFP* plasmid-transfected W+ cells as the control group, and pIB/V5-ATPSβ1(W+) means *ATPSβ1* plasmid-transfected W+ cells as the *ATPSβ1* overexpression group.

Fig 3I

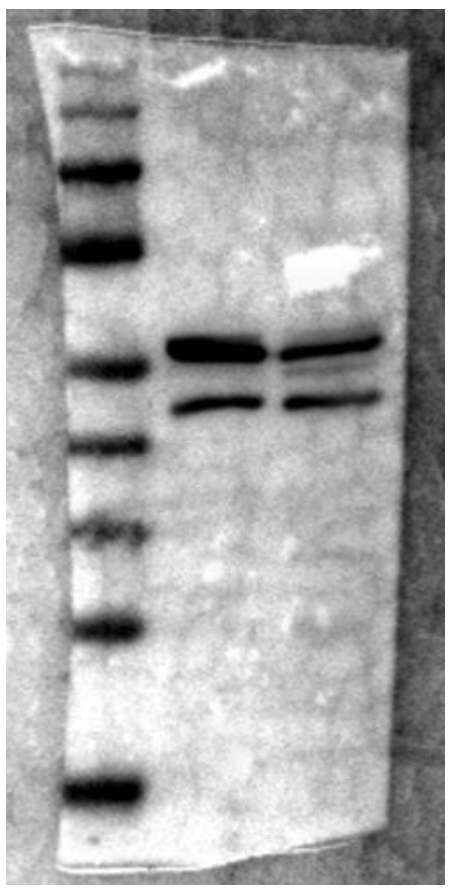

Note: Representative western blot of ATPSβ1 protein in W+ cells transfected with dsGFP (control) or dsATPSβ1 at 48hpt. Lanes 1 and 2 of the above images represent the dsGFP and dsATPSβ1 groups, respectively (From left to right are marker, lanes 1 and 2).

Fig 3J

S1

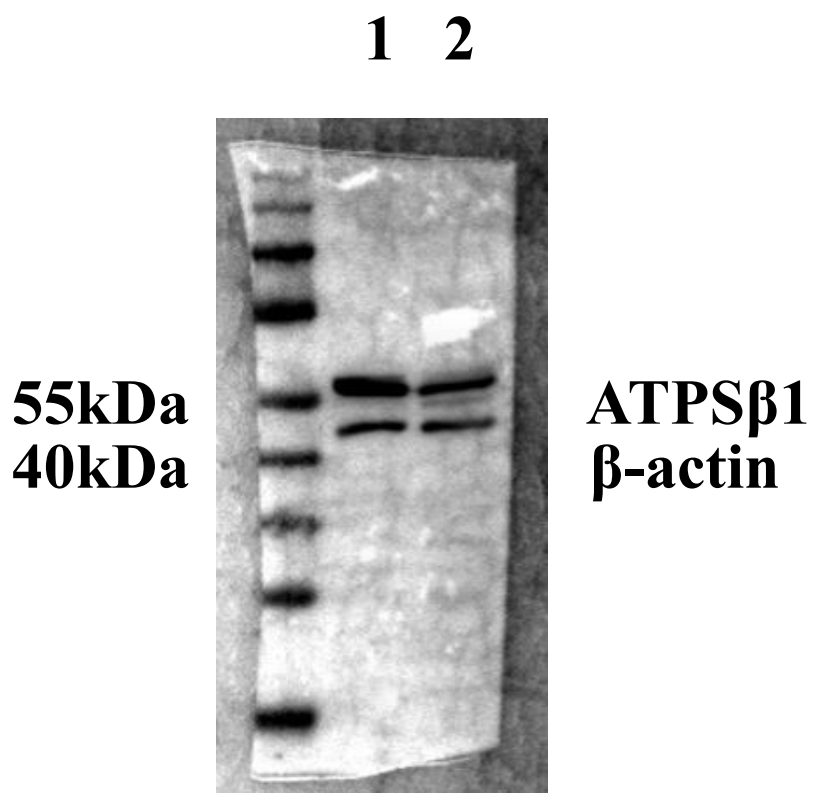

S2

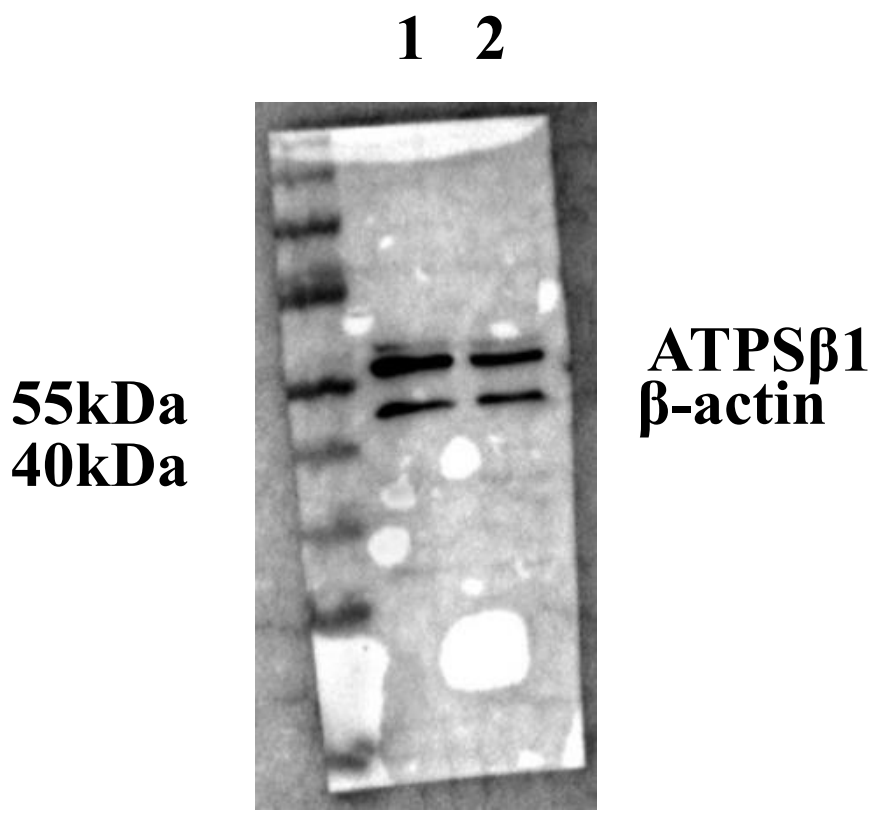

S3

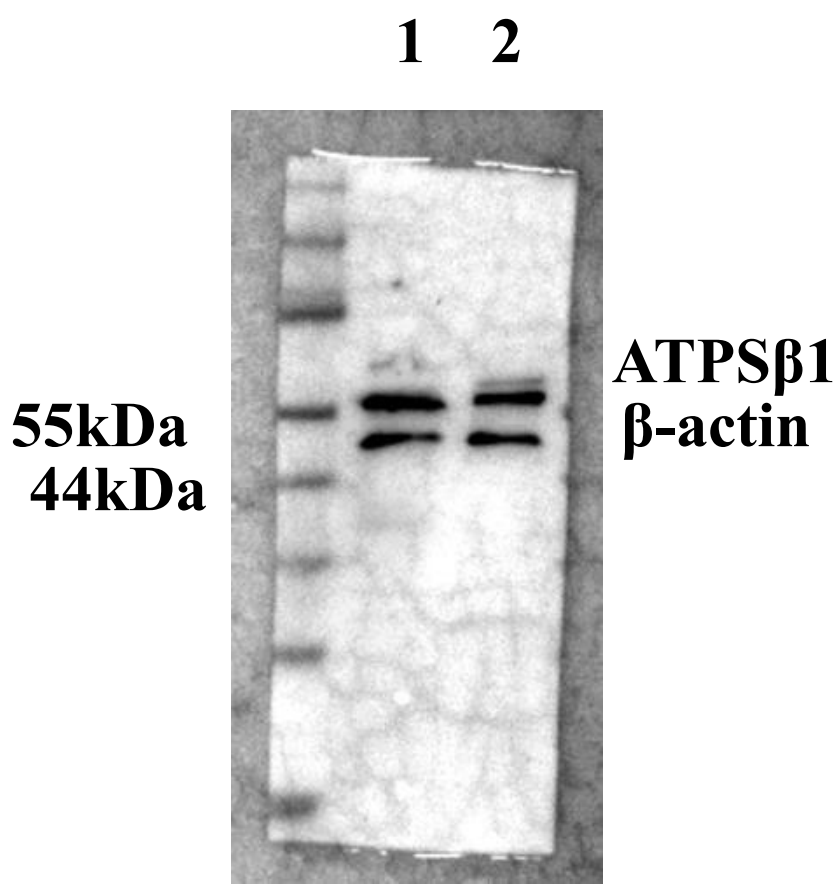

Note: Lanes 1 and 2 of the above images represent the dsGFP and dsATPSβ1 groups groups in W+ cell, respectively (From left to right are marker, lanes 1 and 2).

Fig 3M

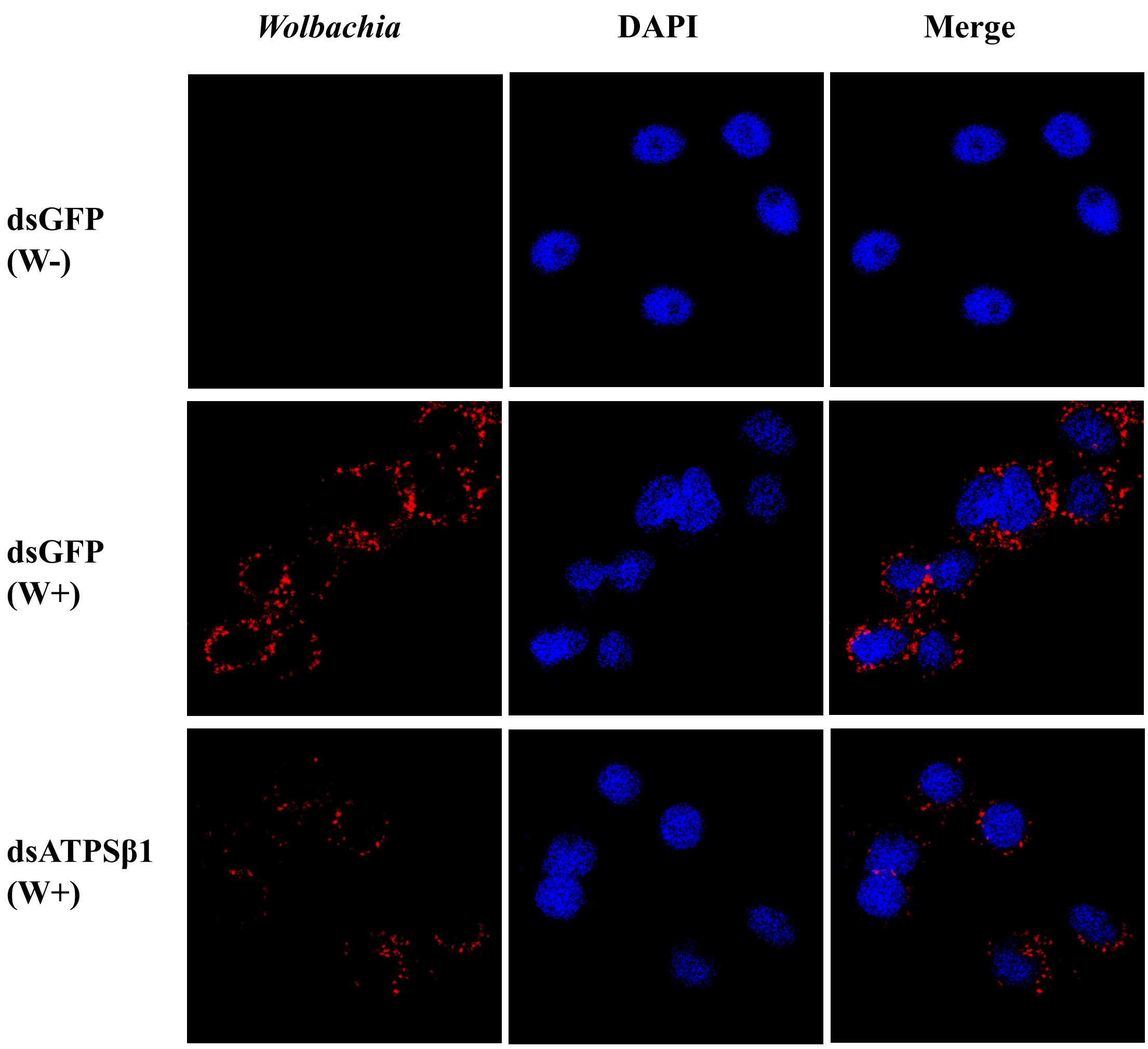

Note: dsGFP(W-) means *GFP* plasmid-transfected W- cells as a negative control for the *Wolbachia* probe, dsGFP(W+) indicates double-stranded RNA of *GFP*-transfected W+ cells as the control group, and dsATPSβ1(W+) indicates double-stranded RNA of *ATPSβ1*-transfected W+ cells as the *ATPSβ1* knockdown group.

Fig 5H

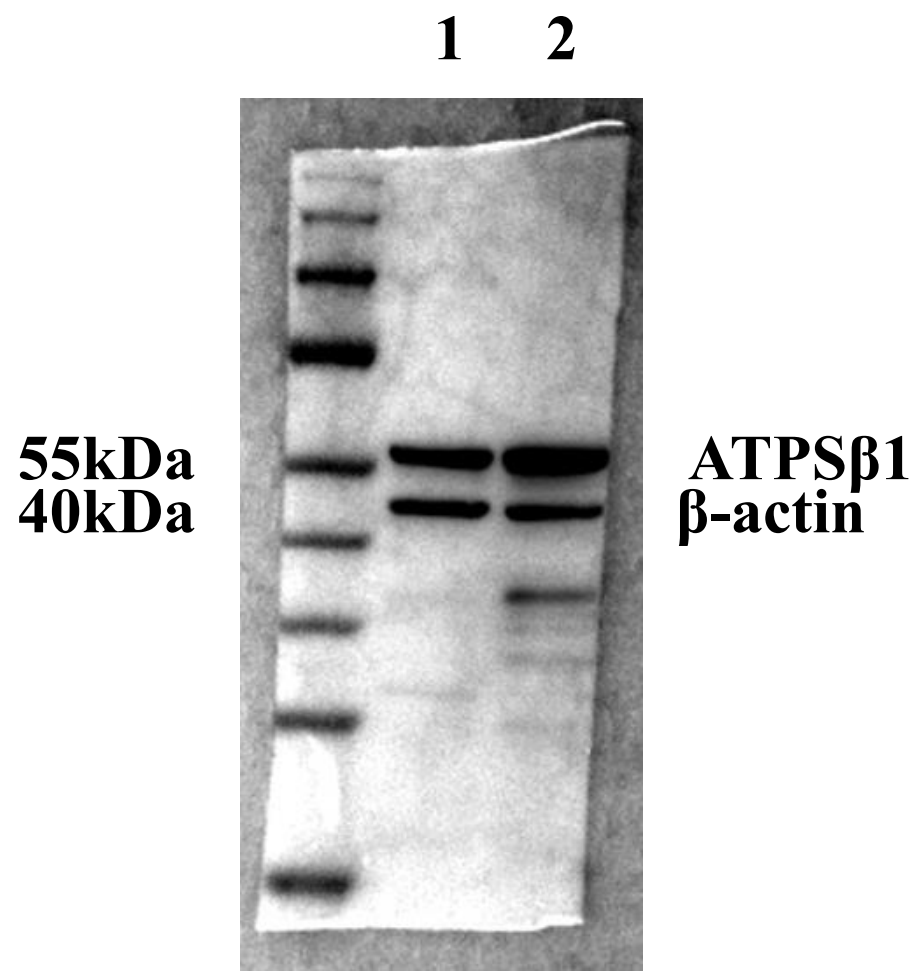

Note: Representative western blot of ATPSβ1 protein in W- cells transfected with pIB/V5-GFP (control) or pIB/V5-ATPSβ1 at 48hpt. Lanes 1 and 2 of the above images represent the pIB/V5-GFP and pIB/V5-ATPSβ1 groups, respectively (From left to right are marker, lanes 1 and 2).

Fig 5I

S1

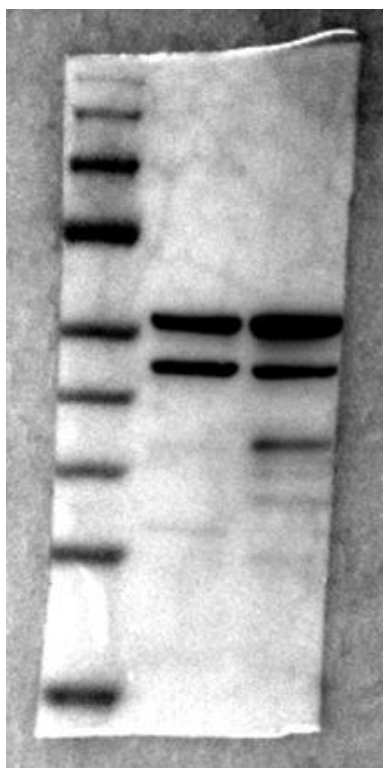

S2

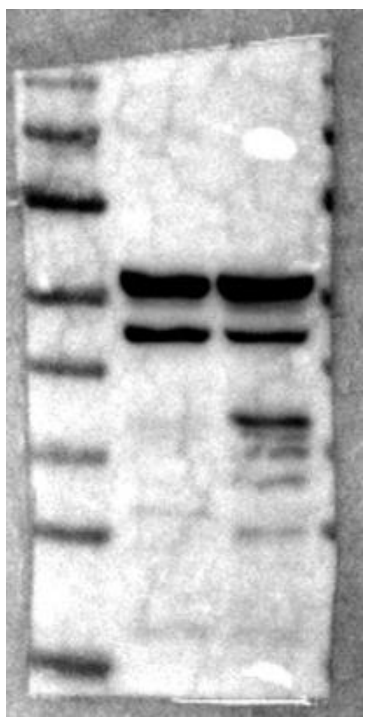

S3

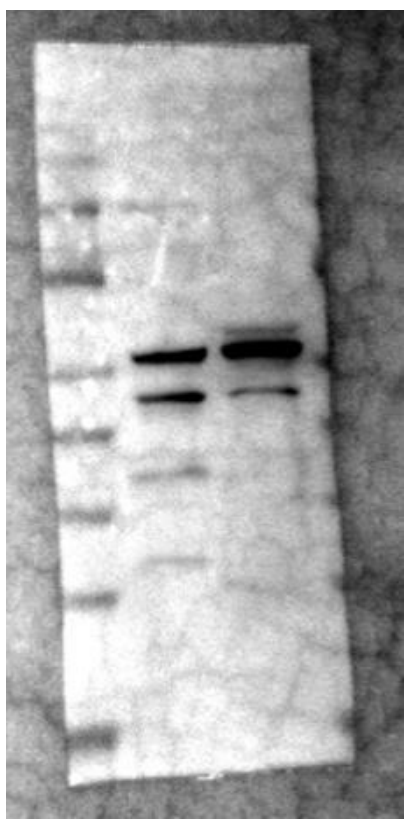

Note: Lanes 1 and 2 of the above images represent the pIB/V5-GFP and pIB/V5-ATPSβ1 groups in W- cell, respectively (From left to right are marker, lanes 1 and 2).

Fig 5L

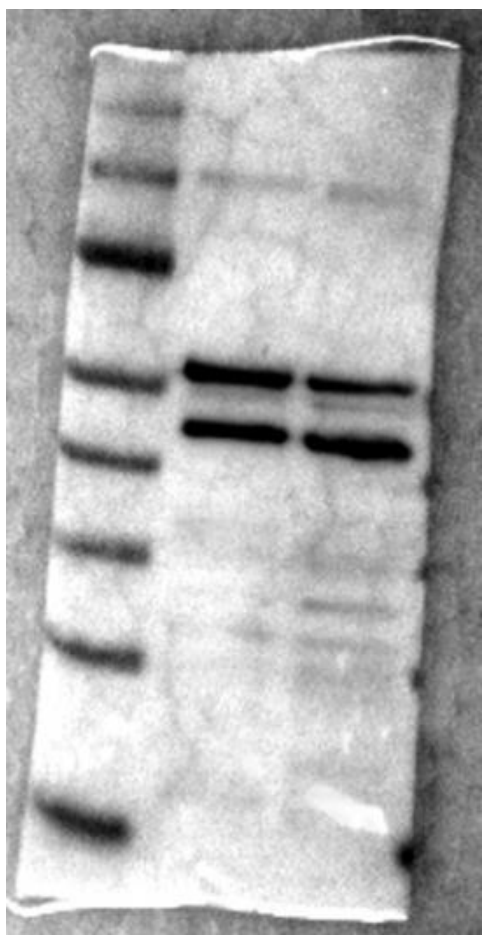

Note: Representative western blot of ATPSβ1 protein in W- cells transfected with dsGFP (control) or dsATPSβ1 at 48hpt. Lanes 1 and 2 of the above images represent the dsGFP and dsATPSβ1 groups, respectively (From left to right are marker, lanes 1 and 2).

Fig 5M

S1

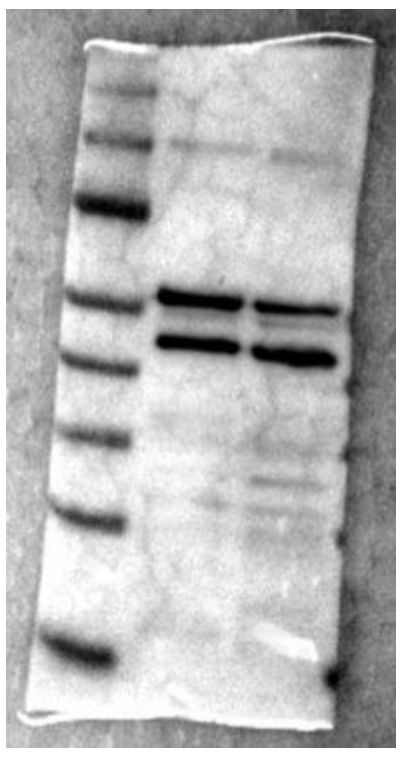

S2

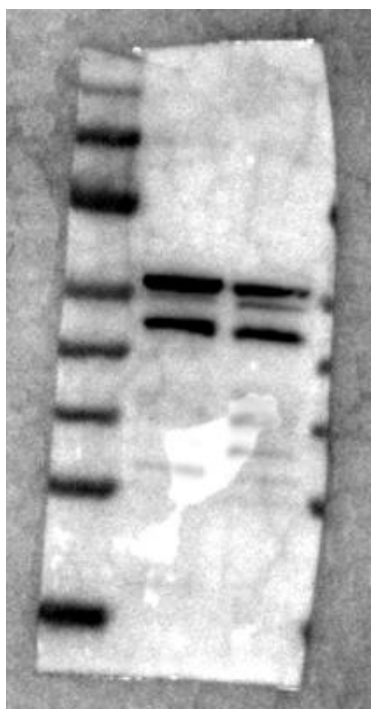

S3

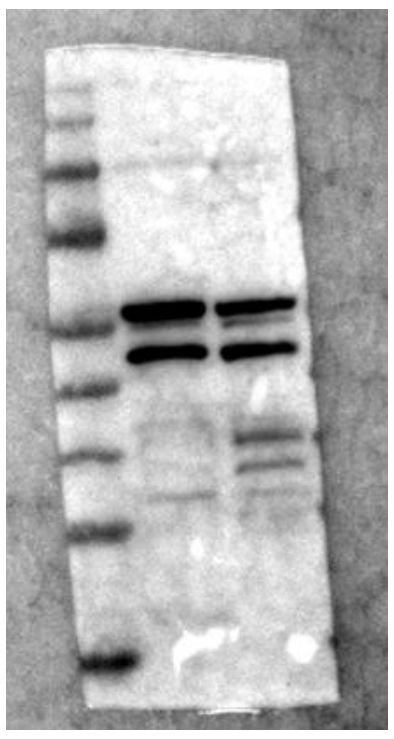

Note: Lanes 1 and 2 of the above images represent the dsGFP and dsATPSβ1 groups groups in W- cell, respectively (From left to right are marker, lanes 1 and 2).

Fig 5Q

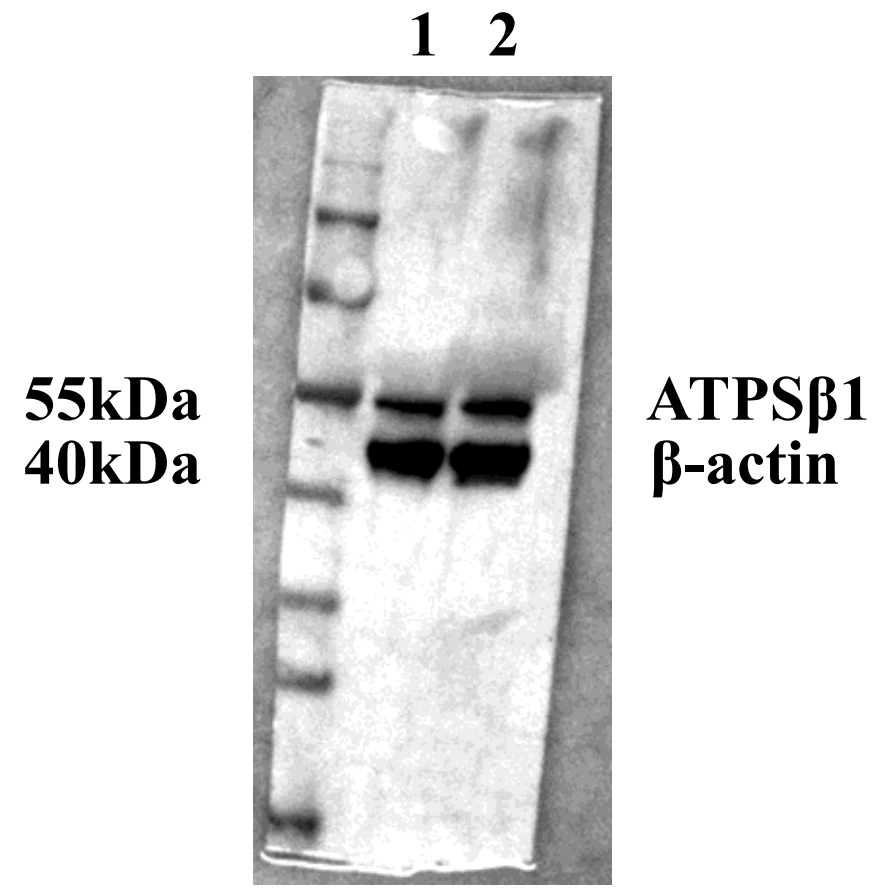

Note: Representative western blot image of ATPSβ1 protein in inactivated VEEV and VEEV infection W- cells at 24 hpi. Lanes 1 and 2 of the above images represent the W-(VEEV-) and W-(VEEV+) groups, respectively (From left to right are marker, 1 and 2 lanes). W-(VEEV-): W- cells infected with inactivated VEEV, W-(VEEV+): VEEV-infected W- cells.

Fig 5R

S1

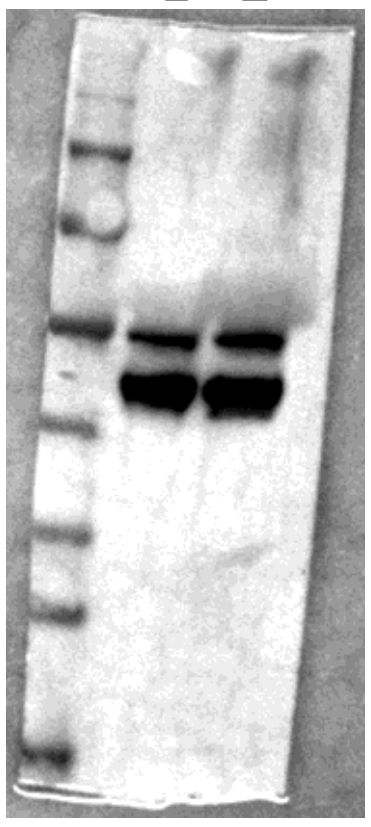

S2

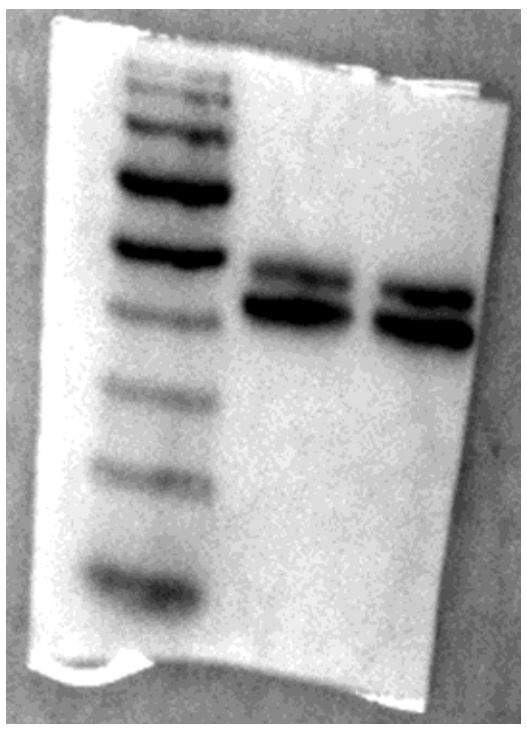

S3

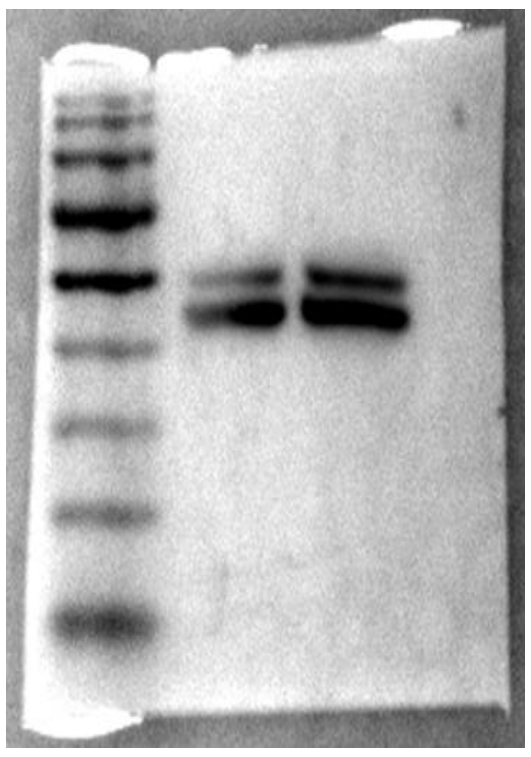

Note: Lanes 1 and 2 of the above images represent the W-(VEEV-) and W-(VEEV+) groups, respectively (From left to right are marker, 1, and 2 lanes).

Fig 5S

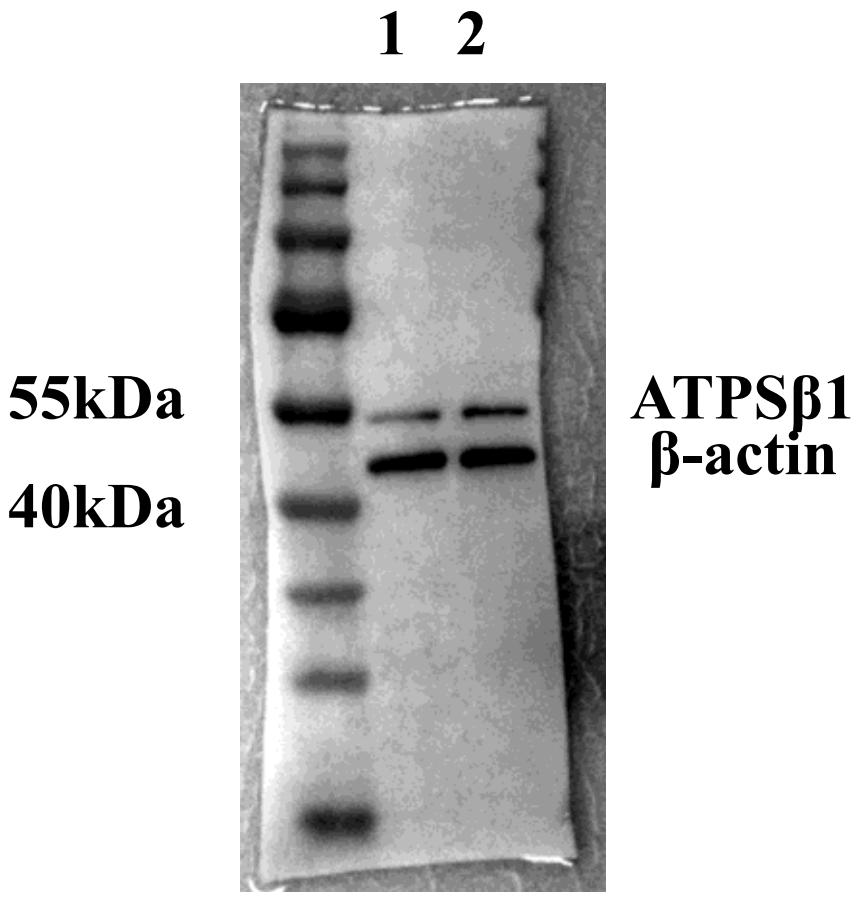

Note: Representative western blot image of ATPSβ1 protein in inactivated DENV and DENV infection W- cells at 48 hpi. Lanes 1 and 2 of the above images represent the W-(DENV-) and W-(DENV+) groups, respectively (From left to right are marker, 1, and 2 lanes). W-(DENV-): W- cells infected with inactivated DENV, W-(DENV+): DENV-infected W- cells.

Fig 5T

S1

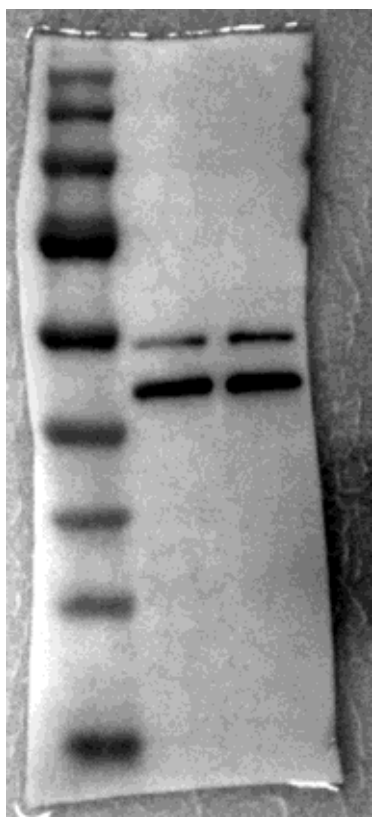

S2

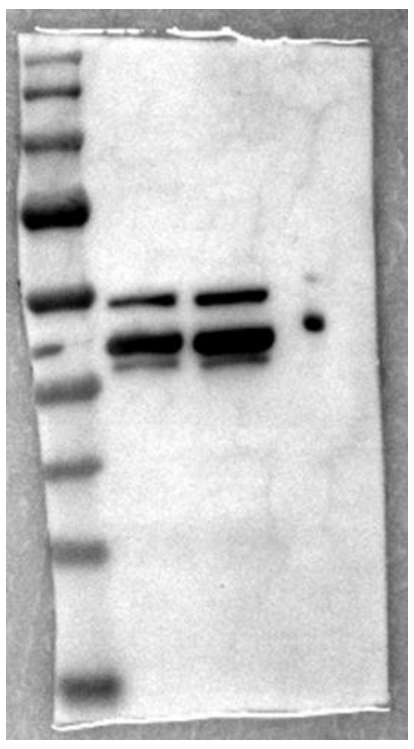

S3

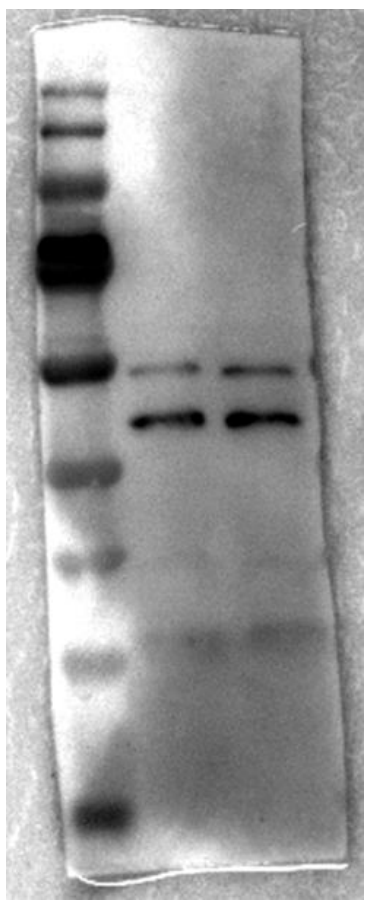

Note: Lanes 1 and 2 of the above images represent the W-(DENV-) and W-(DENV+) groups, respectively (From left to right are marker, 1, and 2 lanes).

Fig 7D

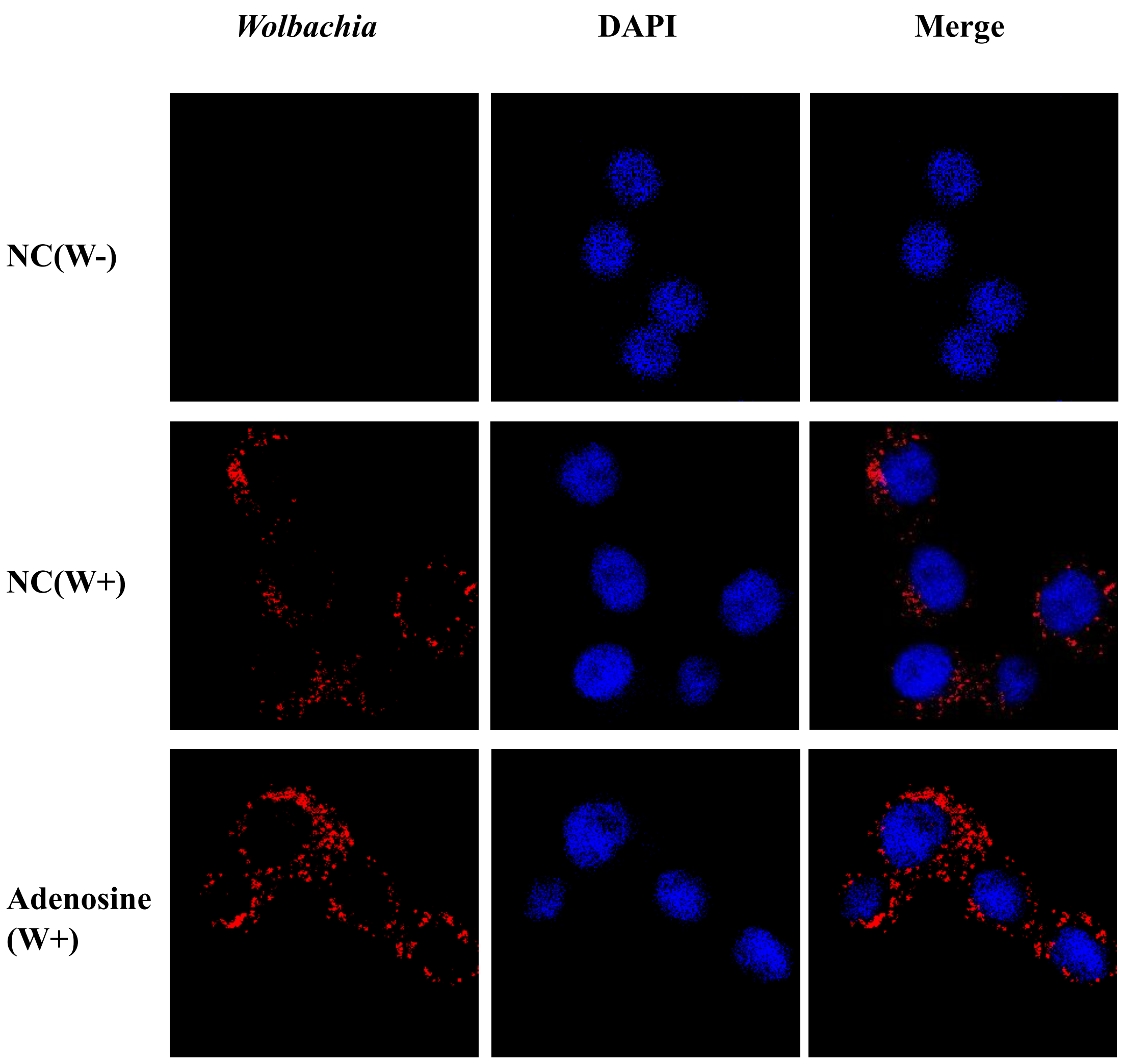

Note: NC(W-) means DMSO treated W- cells as a negative control for the *Wolbachia* probe. Adenosine(W+) indicates 10 μM adenosine treated W+ cells, and NC(W+) indicates DMSO treated W+ cells as the control group.

Fig 9D

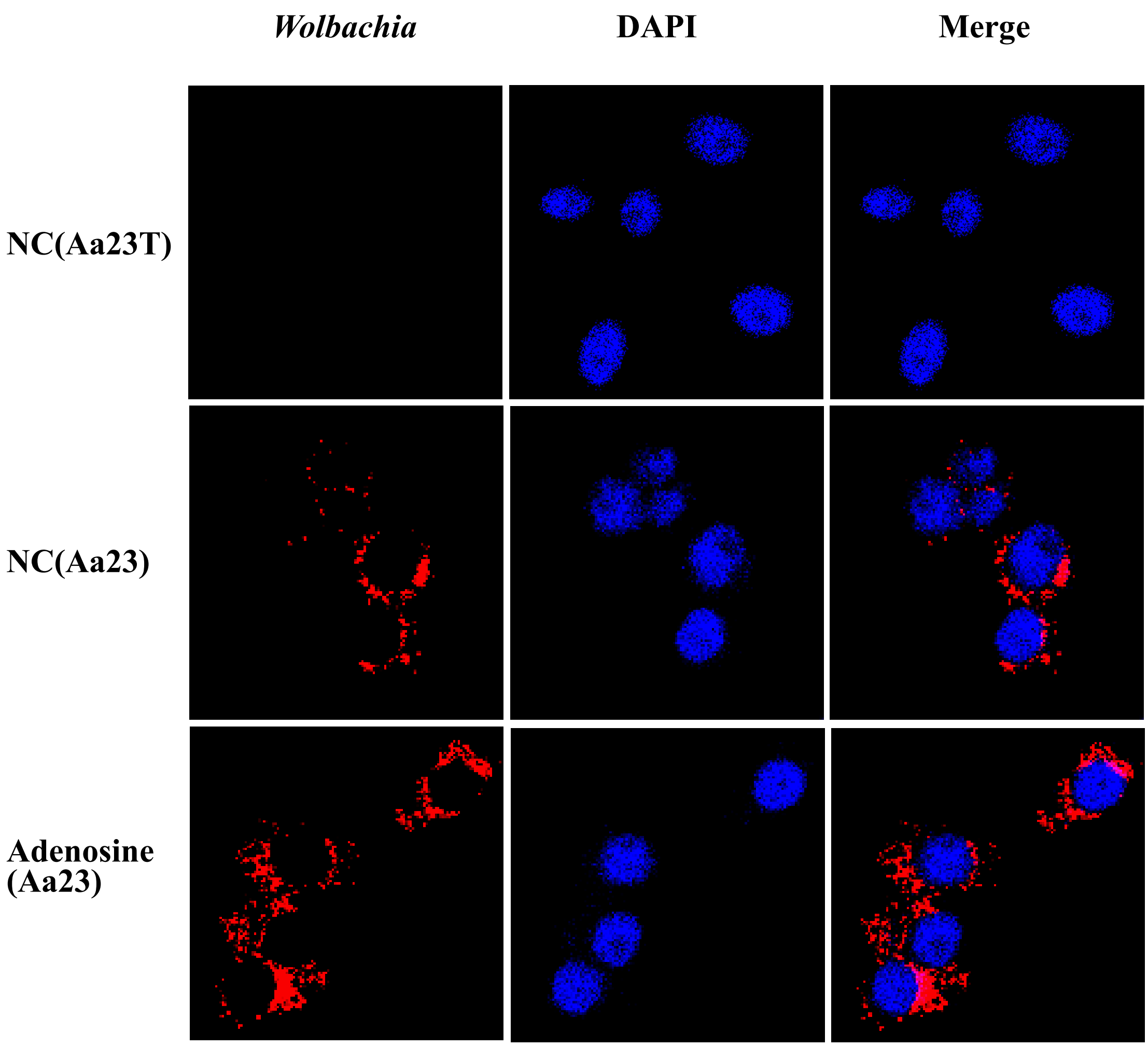

Note: NC(Aa23T) means DMSO treated Aa23T cells as a negative control for the *Wolbachia* probe. Adenosine(Aa23) indicates 10 μM adenosine treated Aa23 cells, and NC(Aa23) indicates DMSO treated Aa23 cells as the control group. Aa23T means *Wolbachia*-free *Aedes albopictus* cells (tetracycline-treated), and Aa23 means *Wolbachia w*AlbA and *w*AlbB infected *Ae. albopictus* cell.

S1 Fig

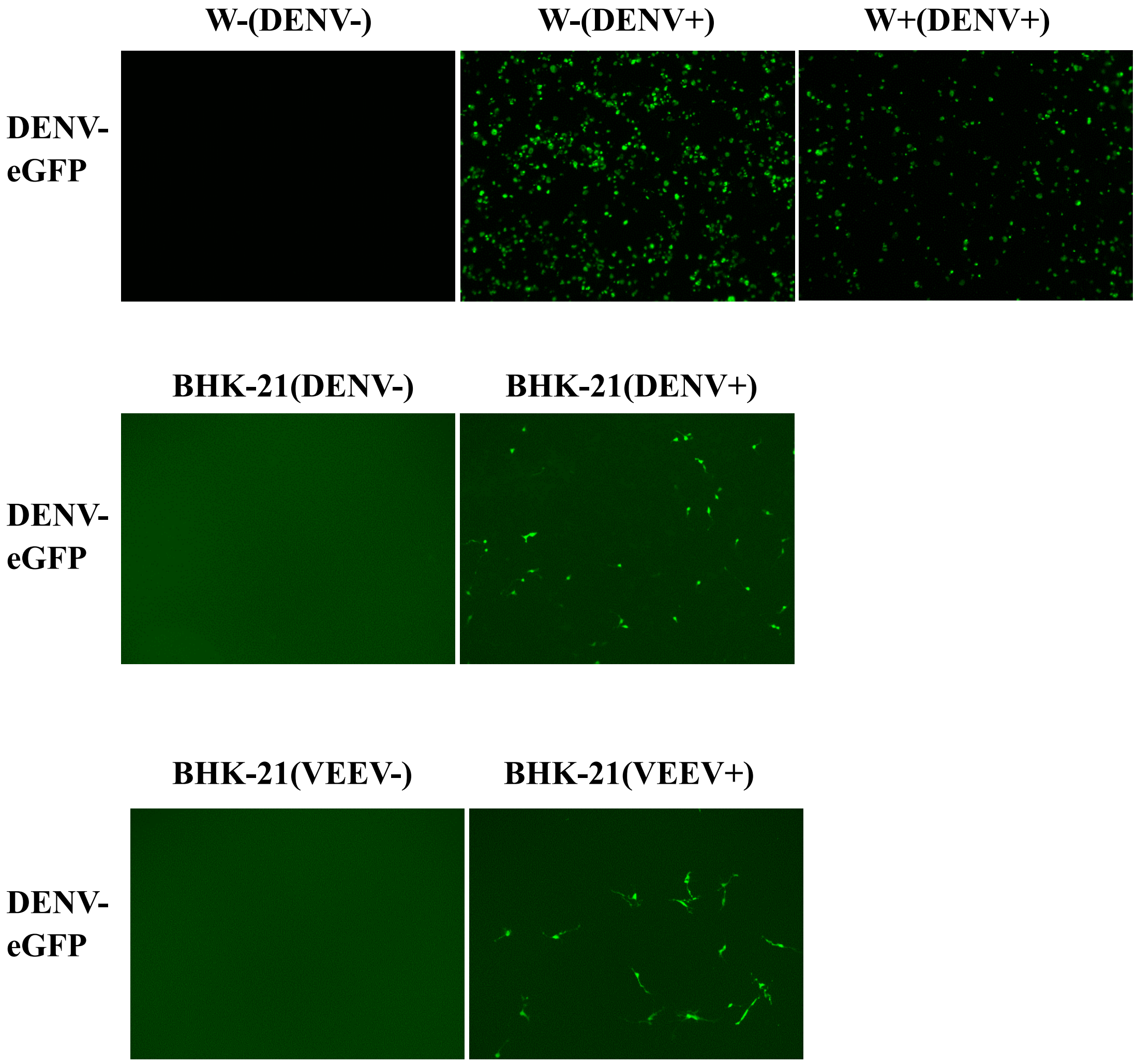

Note: W-(DENV-) refers to W- cells infected with inactivated DENV pseudovirus (negative control); W-(DENV+) and W+(DENV+) refer to W- and W+ cells infected with infectious pseudovirus, respectively; BHK-21(VEEV-) and BHK-21(VEEV+) refer to BHK-21 cells infected with inactivated and infectious VEEV pseudovirus, respectively; BHK-21(DENV-) and BHK-21(DENV+) refer to BHK-21 cells infected with inactivated and infectious DENV pseudovirus, respectively.

S4 Fig

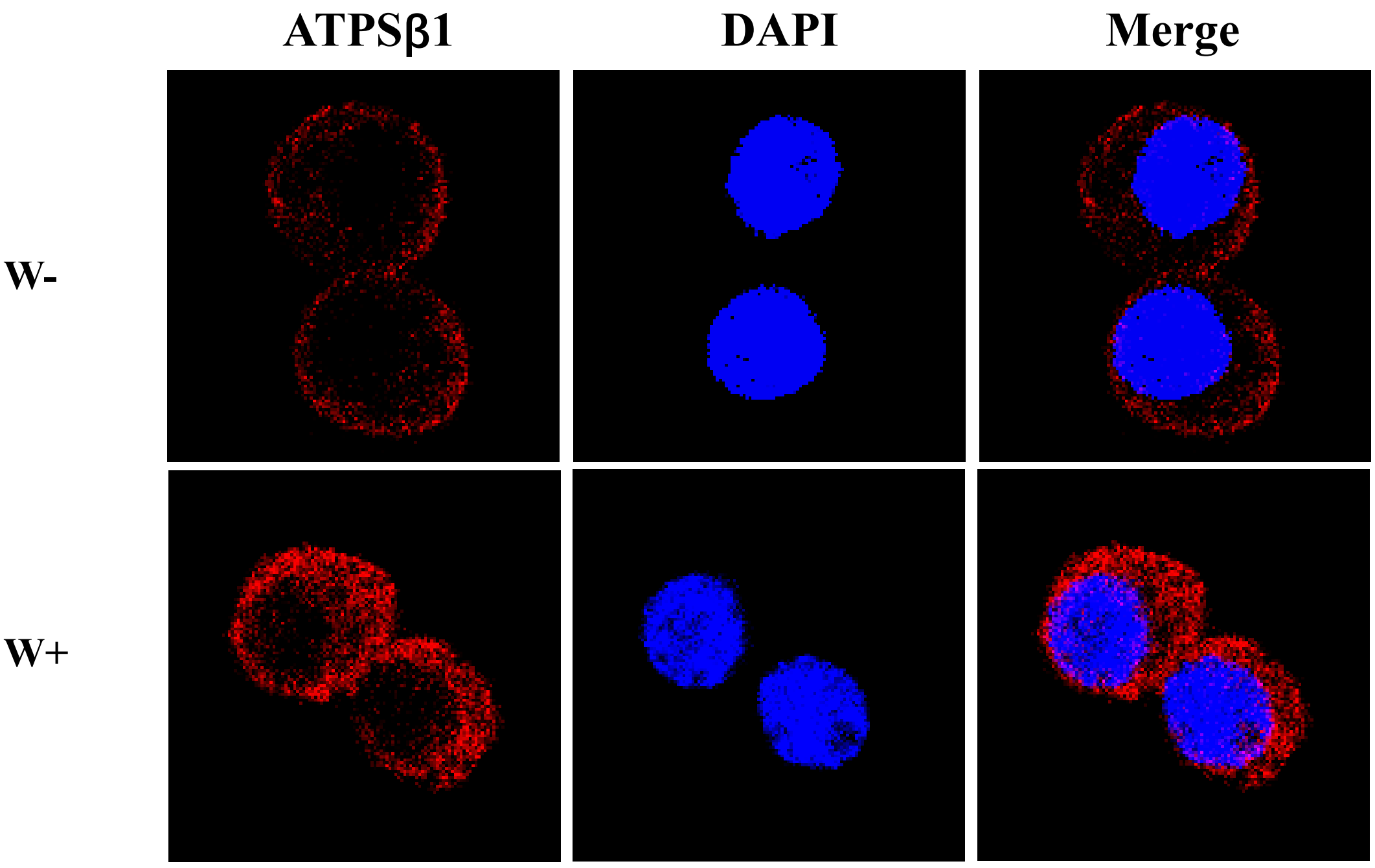

Note: W- means *Wolbachia* free *Ae. aegypti* cells, and W+ means *Wolbachia w*AlbB infected *Ae. aegypti* cells.
