# Supplementary material for: Wolbachia outcompetes mosquito-borne viruses by reprogramming host ATPSβ1-driven ATP metabolism toward symbiotic proliferation
Source: PLoS Pathog. 2026 Oct 21;22(10):e1014692. doi: 10.1371/journal.ppat.1014692 (PMC13712716; doi:10.1371/journal.ppat.1014692)
Supplement: Supplementary file 2 [file ppat.1014692.s010.docx]

**S2 Table. The primer information used in quantitative PCR.**

| **Gene Name** | **ID** | **Primer Name** | **Sequence (5'-3')** |
| --- | --- | --- | --- |
| *ATPSβ1* | AAEL002827 | ATPSβ1-F  ATPSβ1-R | GCAAGGAAAACAGATCGCCC  CGATTGCGAGATTTGGGCTG |
| *V-ATPase D1* | AAEL011025 | V-ATPase D1-F  V-ATPase D1-R | CGGAAACCTCCAGAGTGAAGT  GATTCCGCACTTGAAGCCAC |
| *Ndufs1* | AAEL012552 | Ndufs1-F  Ndufs1-R | ATTTTCTCGCTTTCGGTGCC  TATTACCCTGGTCAACGGCG |
| *Ndufa9* | AAEL009414 | Ndufa9-F  Ndufa9-R | GGCAGCAAGTTCCTCAAGAG  CCTCCTGTCCGTAGATGTCC |

Note: *ATPSβ1*: ATP synthase beta subunit 1; *V-ATPase D1*: V-type proton ATPase subunit d 1; *Ndufa9*: NADH dehydrogenase 1 alpha subcomplex subunit 9; *Ndufs1*: NADH-ubiquinone oxidoreductase 75 kDa subunit.
