# Supplementary material for: Wolbachia outcompetes mosquito-borne viruses by reprogramming host ATPSβ1-driven ATP metabolism toward symbiotic proliferation
Source: PLoS Pathog. 2026 Oct 21;22(10):e1014692. doi: 10.1371/journal.ppat.1014692 (PMC13712716; doi:10.1371/journal.ppat.1014692)
Supplement: Supplementary file 3 [file ppat.1014692.s011.docx]

**S3 Table. The sequences used for the prediction of complexes by AlphaFold3.**

| **Protein Name** | **Amino acid sequence** |
| --- | --- |
| ***Ae. aegypti* ATPSβ1** | MFSSMKTLMAVAARAEQVVARGYAAKAAAKAAAGAQGKVVAVIGAVVDVQFDDNLPPILNALEVQERGSRLVLEVAQHLGENTVRTIAMDGTEGLVRGQRVLDTGSPIRIPVGAETLGRIINVIGEPIDERGPIETNLSAPIHAEAPEFIDMSVEQEILVTGIKVVDLLAPYAKGGKIGLFGGAGVGKTVLIMELINNVAKAHGGYSVFAGVGERTREGNDLYNEMIEGGVISLKDKTSKVALVYGQMNEPPGARARVALTGLTVAEYFRDQEGQDVLLFIDNIFRFTQAGSEVSALLGRIPSAVGYQPTLATDMGSMQERITTTKKGSITSVQAIYVPADDLTDPAPATTFAHLDATTVLSRAIAELGIYPAVDPLDSTSRIMDPNIIGAEHYNIARGVQKILQDYKSLQDIIAILGMDELSEEDKLTVARARKIQRFLSQPFQVAEVFTGHAGKLVPLEETIKGFTKILNGELDHLPEVAFYMVGPIEEVVEKAERLAKEAA |
| **VEEV E2** | STEELFNEYKLTRPYMARCIRCAVGSCHSPIAIEAVKSDGHDGYVRLQTSSQYGLDSSGNLKGRTMRYDMHGTIKEIPLHQVSLYTSRPCHIVDGHGYFLLARCPAGDSITMEFKKDSVRHSCSVPYEVKFNPVGRELYTHPPEHGVEQACQVYAHDAQNRGAYVEMHLPGSEVDSSLVSLSGSSVTVTPPDGTSALVECECGGTKISETINKTKQFSQCTKKECQRAYRLQNDKWVYNSDKLPKAAGATLKGKLHVPFLLADGKCTVPLAPEPMITFGFRSVSLKLHPKNPTYLITRQLADEPHYTHELISEPAVRNFTVTEKGWEFVWGNHPPKRFWAQETAPGNPHGLPHEVITHYYHRYPMSTILGLSICAAIATVSVAASTWLFCRSRVACLTPYRLTPNARIPFCLAVLCCARTARA |
| **DENV E** | MRCIGMSNRDFVEGVSGGSWVDIVLEHGSCVTTMAKNKPTLDFELIKTEAKQPATLRKYCIEAKLTNTTTESRCPTQGEPSLNEEQDKRFVCKHSMVDRGWGNGCGLFGKGGIVTCAMFRCKKNMEGKVVQPENLEYTIVITPHSGEEHAVGNDTGKHGKEIKITPQSSITEAELTGYGTVTMECSPRTGLDFNEMVLLQMENKAWLVHRQWFLDLPLPWLPGADTQGSNWIQKETLVTFKNPHAKKQDVVVLGSQEGAMHTALTGATEIQMSSGNLLFTGHLKCRLRMDKLQLKGMSYSMCTGKFKVVKEIAETQHGTIVIRVQYEGDGSPCKIPFEIMDLEKRHVLGRLITVNPIVTEKDSPVNIEAEPPFGDSYIIIGVEPGQLKLNWFKKGSSIGQMFETTMRGAKRMAILGDTAWDFGSLGGVFTSIGKALHQVFGAIYGAAFSGVSWTMKILIGVIITWIGMNSRSTSLSVTLVLVGIVTLYLGVMVQA |
