# Supplementary material for: Wolbachia outcompetes mosquito-borne viruses by reprogramming host ATPSβ1-driven ATP metabolism toward symbiotic proliferation
Source: PLoS Pathog. 2026 Oct 21;22(10):e1014692. doi: 10.1371/journal.ppat.1014692 (PMC13712716; doi:10.1371/journal.ppat.1014692)
Supplement: Supplementary file 4 [file ppat.1014692.s012.docx]

**S4 Table. The primers used for double-stranded RNA synthesis.**

|  | **Gene Name** | **ID** | **Primer Name** | **Sequence (5'-3)** |
| --- | --- | --- | --- | --- |
|  | *ATPSβ1* | AAEL002827 | dsATPSβ-F  dsATPSβ-R | CATAAGGGCTGAGGATCCAA  CAATCTTACCTCCCTTGGCA |
| *GFP* | | MN517551.1 | dsGFP-F  dsGFP-R | CTGGAGTGGTCCCAGTTCTTG  GAAAGGGCAGATTGTGTGGAC |
|  | *RLUC* | MN030571.1 | dsRLUC-F  dsRLUC-R | GTAACGCTGCCTCCAGCTAC  GCCTGACATCGAGGAGGATA |

Note: The T7 promoter sequence TAATACGACTCACTATAGGG was added to the 5' end of the above sequence for primer synthesis.
